# Supplementary material for: Pd-catalyzed fluoro-carbonylation of aryl, vinyl, and heteroaryl iodides using 2-(difluoromethoxy)-5-nitropyridine
Source: Commun Chem. 2020 May 11;3:59. doi: 10.1038/s42004-020-0304-3 (PMC9814140; doi:10.1038/s42004-020-0304-3)
Supplement: Supplementary file 1 — Supplementary Information [file 42004_2020_304_MOESM1_ESM.pdf]

**Supplementary Information**

# Pd-catalyzed fluoro-carbonylation of aryl, vinyl, and heteroaryl iodides using 2-(difluoromethoxy)-5-nitropyridine

Yumeng Liang[1], Zhengyu Zhao[1], and Norio Shibata*[,1,2]

[1]*Department of Nanopharmaceutical Sciences & Department of Life Science and Applied Chemistry, Nagoya Institute of Technology, Gokiso, Showa-ku, Nagoya, 466-8555, Japan.*

[2]*Institute of Advanced Fluorine-Containing Materials, Zhejiang Normal University, 688 Yingbin Avenue, 321004 Jinhua, China.*

*Correspondence and requests for materials should be addressed to N.S. (e-mail: nozshiba@nitech.ac.jp.)*

**Supplementary Methods**

**General Information.**

All reagents were used as received from commercial sources, unless specified otherwise. All reactions were performed in oven-dried glassware under a positive pressure of nitrogen. All reactions were monitored by thin-layer chromatography (TLC) carried out on 0.25 mm Merck silica-gel (60-F254). The TLC plates were visualized with UV light (254 nm) and potassium permanganate. Purification of reaction products was carried out by column chromatography with silica-gel 60N spherical neutral size 63-210 μm or 40-63 μm. Unless otherwise specified, the $^{1}$H-NMR (300 MHz), $^{1}$H-NMR (500 MHz), $^{19}$F-NMR (282 MHz), $^{13}$C-NMR (126 MHz) spectra for solution in $CDCl_3$ were recorded on a Buruker Avance 500, Varian Mercury 300. Chemical shifts (δ) are expressed in ppm downfield from tetramethylsilane (δH = 0.00 ppm) or tetramethylsilane (δC = 77.0 ppm) or hexafluorobenzene (δF = −162.20 ppm). Mass spectra were recorded on a SHIMADZU LCMS-2020EV (ESI-MS) system and a JEOL JMS-Q1050GC Master-Quad GC/MS (EI) system. High resolution mass spectrometry (HRMS) was recorded on a Waters Synapt G2 HDMS (ESI-MS) and a SHIMADZU GCMS-QP5050A (EI-MS). The wave numbers (*v*) of recorded IR-signals are quoted in $cm^{-1}$ on a JASCO FT/IR-4100 spectrometer. Melting point was recorded on a BUCHI M-565. All solvents were dried and distilled before use.

**Optimization of Reaction Conditions (Supplementary Tables 1-4).**

**Supplementary Table 1.** Optimization of reaction condition.

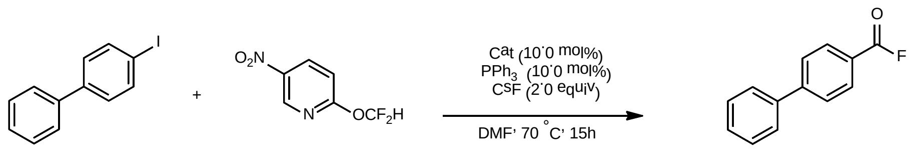


| Entry | Cat. | Yield (%)[a] |
|---|---|---|
| 1 | CuI | NR |
| 2 | AgOAc | NR |
| 3 | $Ni(OTf)_2$ | NR |
| 4 | $Fe(OAc)_2$ | NR |
| 5 | $CoCl_2$ | NR |
| 6 | $RuCl_2(Ph_3P)_3$ | NR |
| 7 | $Pd(OAc)_2$ | 73 |

Reaction conditions: **1a** (0.1 mmol), **2** (0.2mmol 2.0 equiv), base (0.2 mmol, 2.0 equiv), **Cat.** (10.0 mol%) and $PPh_3$ (10.0 mol%) were stirred in 1.5 mL of anhydrous DMF at 70 °C 15 hours. [a] Determined by $^{19}$F NMR spectroscopy.

**Supplementary Table 2.** Optimization of reaction condition.

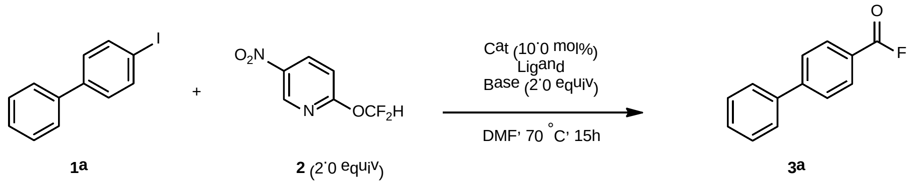


| Entry | Cat. | Ligand (Pd:P = 1:x) | Base | Yield (%)[a] |
|---|---|---|---|---|
| 1 | $Pd(OAc)_2$ | -- | CsF | 18 |
| 2 | $Pd(OAc)_2$ | $PPh_3$ (1:1) | CsF | 73 |
| 3 | $Pd(OAc)_2$ | $PPh_3$ (1:2) | CsF | 77 |
| 4 | $Pd(OAc)_2$ | $PPh_3$ (1:3) | CsF | 84 |
| 5 | $Pd(OAc)_2$ | $PPh_3$ (1:4) | CsF | 77 |
| 6 | $Pd(OAc)_2$ | $PPh_3$ (1:3) | KF | 45 |
| 7 | $Pd(OAc)_2$ | $PPh_3$ (1:3) | NaF | NR |
| 8 | $Pd(OAc)_2$ | $PPh_3$ (1:3) | LiF | NR |
| 9 | $PdCl_2\cdot(CH_3CN)_2$ | $PPh_3$ (1:3) | CsF | 89 |
| 10 | $Pd(0)(dba)_2$ | $PPh_3$ (1:3) | CsF | 83 |
| 11 | $[PdCl(allyl)]_2$ | $PPh_3$ (1:3) | CsF | 75 |
| 12 | $Pd(0)(PPh_3)_4$ | -- | CsF | 83 |
| 13 | $Pd(C_5HF_6O_2)_2$ | $PPh_3$ (1:3) | CsF | 83 |
| 14 | $Pd(TFA)_2$ | $PPh_3$ (1:3) | CsF | 95 |
| 15 | $Pd_2(0)(dba)_3$ | $PPh_3$ (1:3) | CsF | 88 |
| 16 | $PdCl_2$ | $PPh_3$ (1:3) | CsF | 86 |
| 17 | $PdBr_2$ | $PPh_3$ (1:3) | CsF | 90 |
| 18 | $Pd(TFA)_2$ | P(*o*-Tol)$_3$ (1:3) | CsF | 40 |
| 19 | $Pd(TFA)_2$ | TFP (1:3) | CsF | 83 |
| 20 | $Pd(TFA)_2$ | DPPE (1:3) | CsF | 74 |
| 21 | $Pd(TFA)_2$ | PCy3 (1:3) | CsF | 43 |
| 22 | $Pd(TFA)_2$ | *rac*-BINAP (1:3) | CsF | 84 |
| 23 | $Pd(TFA)_2$ | DPEphos (1:3) | CsF | 86 |
| 24 | $Pd(TFA)_2$ | Xantphos (1:3) | CsF | >99 (92) |
| 25 | $Pd(TFA)_2$ | Xphos (1:3) | CsF | 49 |

Reaction conditions: **1a** (0.1 mmol), **2** (0.2mmol 2.0 equiv), base (0.2 mmol, 2.0 equiv), **cat.** (10.0 mol%) and ligand were stirred in 1.5 mL of anhydrous DMF at 70 °C 15 hours. [a]Determined by $^{19}F$ NMR spectroscopy. In parentheses isolated yields.

**Supplementary Table 3.** Optimization of reaction condition.

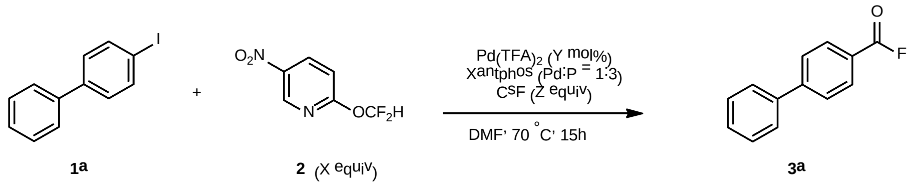

| Entry | **2** (X equiv) | $Pd(TFA)_2$ (Y equiv) | CsF (Z equiv) | Yield (%)[a] |
|---|---|---|---|---|
| 1 | 2 | 10 | 1.5 | 98 (92%) |
| 2 | 2 | 10 | 1.0 | 78 |
| 3 | 2 | 10 | 0.5 | 21 |
| 4 | 1.5 | 10 | 1.5 | 85 |
| 5 | 1.2 | 10 | 1.2 | 84 |
| 6 | 1.5 | 10 | 2.0 | >99 |
| 7[b] | 1.5 | 10 | 2.0 | >99 |
| 8[b] | 1.5 | 5 | 2.0 | >99 |
| 9[b] | 1.5 | 2 | 2.0 | >99 |
| 10[b] | 1.5 | 1 | 2.0 | >99 |
| 11[b] | 1.2 | 1 | 2.0 | >99 |
| 12[b] | 1.2 | 1 | 1.5 | >99 (92) |
| 13[b] | 1.1 | 1 | 1.5 | 94 |
| 14[b] | 1.1 | 1 | 1.2 | 90 |

Reaction conditions: **1a** (0.1 mmol), **2**, CsF, $Pd(TFA)_2$ and Xantphos were stirred in 1.5 mL of anhydrous DMF at 70 °C 15 hours.

[a]Determined by $^{19}F$ NMR spectroscopy. In parentheses isolated yields. [b]1a (0.3 mmol) was used.

**Supplementary Table 4.** Optimization of reaction condition.

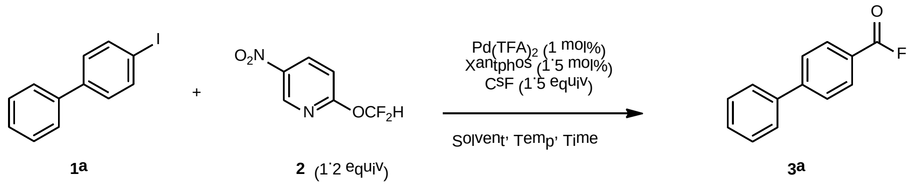


| Entry | Solvent | Temp. (°C) | Time (h) | Yield (%)[a] |
|---|---|---|---|---|
| 1 | DMF | 70 | 15 | >99 (92) |
| 2 | DMSO | 70 | 15 | 78 |
| 3 | MeCN | 70 | 15 | 59 |
| 4 | THF | 70 | 15 | trace |
| 5 | DCE | 70 | 15 | 3 |
| 6 | 1,4-dioxane | 70 | 15 | 3 |
| 7 | Toluene | 70 | 15 | NR |
| 8[b] | DMF | 70 | 15 | >99 (92) |
| 9[c] | DMF | 70 | 15 | >99 (92) |
| 10[d] | DMF | 70 | 15 | 88 |
| 11[c] | DMF | 50 | 15 | 62 |
| 12[c] | DMF | rt | 15 | 52 |
| 13[c] | DMF | 100 | 15 | 92 |
| 14[c] | DMF | 70 | 8 | 88 |

Reaction conditions: **1a** (0.3 mmol), **2** (0.36 mmol, 1.2 equiv), CsF (0.45 mmol, 1.5 equiv), Pd(TFA)$_2$ (1.0 mol%) and Xantphos (1.5 mol%)were stirred in 4.5 mL of anhydrous solvent. [a]. Determined by $^{19}$F NMR spectroscopy. In parentheses isolated yields. [b]3.0 mL of anhydrous DMF was used. [c]2.0 mL of anhydrous DMF was used. [d]1.0 mL of anhydrous DMF was used.

**Preparation of Iodide Compounds and Characterization Data for 1w, 1z − 1zc and 1zi (Supplementary Figures 1-6).**

Iodide compounds **1a** − **1n**, **1q** and **1t** were known compounds and obtained from commercial sources. Iodide compounds **1o**[1], **1p**[1], **1r**[2], **1s**[3] **1u**[4], **1x**[5], **1y**[6] **and 1zj**[7] were known compounds and were synthesized according to the reported procedures.

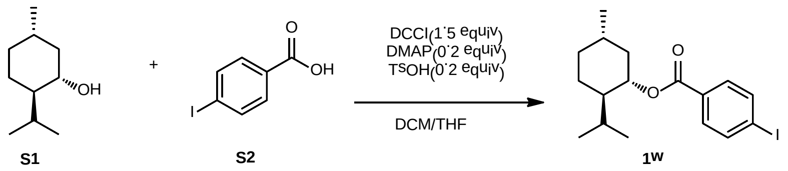


**Supplementary Figure 1.** Preparation of (1*S*,2*R*,5*S*) -2-isopropyl-5-methylcyclohexyl 4-iodobenzoate (**1w**).

**(1*S*,2*R*,5*S*) -2-isopropyl-5-methylcyclohexyl 4-iodobenzoate (1w).** An oven-dried round-flask with a rubber septum and a magnetic stir bar was charged with (+)-Menthol (**S1**) (4.0 mmol, 1.0 equiv), *N*,*N'*-Dicyclohexylcarbodiimide (DCCI, 6.0 mmol, 1.5 equiv), 4-Dimethylaminopyridine (DMAP, 0.8 mmol, 0.2 equiv) and anhydrous DCM/THF (20 mL 4:1 v/v) at room temperature under a positive pressure of nitrogen gas. Then, the mixtures were cooled down to 0 °C with an ice bath 10 min, 4-iodobenzoic acid (**S2**, 4.0 mmol, 1.0 equiv) dissolved in DCM/THF (10 mL, 4:1 v/v) was added dropwise to the mixture under stirring via dropping funnel. When the additions were complete, the reaction mixture was allowed to stir at room temperature overnight. After the reaction was complete (monitored by TLC), the *N*,*N'*-dicyclohexylurea was filtered off and the filtrate was washed with $H_2O$ (3 × 30 mL), dried over anhydrous $Na_2SO_4$, filtered and concentrated under reduced pressure. The residue was purified by silica gel flash chromatography (*n*-Hexane: Chloroform = 4:1) to afford the title product (1.42 g, 92% yield) as a colorless viscous oil. **$^1$H NMR** (300 MHz, $CDCl_3$) δ: 7.81 –7.73 (m, 4H), 4.92 (ddd, *J* = 10.9, 4.1 Hz, 1H), 2.11 (d, *J* = 12.3 Hz, 1H), 1.92 (p, *J* = 7.2 Hz, 1H), 1.72 (d, *J* = 11.9 Hz, 2H), 1.54 (t, *J* = 11.6 Hz, 2H), 1.26 – 0.98 (m, 3H), 0.94 – 0.90 (m, 6H), 0.78 (d, *J* = 7.0 Hz, 3H) ppm. **$^{13}$C NMR** (126 MHz, $CDCl_3$) δ: 165.5, 137.6, 131.0, 130.2, 100.4, 75.1, 47.2, 40.9, 34.2, 31.4, 26.5, 23.6, 22.0, 20.7, 16.5 ppm. **HRMS** (TOF/EI$^+$): Calculated for $C_{17}H_{23}IO_3^+$: 386.0743, found: 386.0740. **IR** (NaCl): 2954, 2927, 2867, 1714, 1585, 1456, 1390, 1369, 1272, 1176, 1008, 845, 548 cm$^{-1}$.

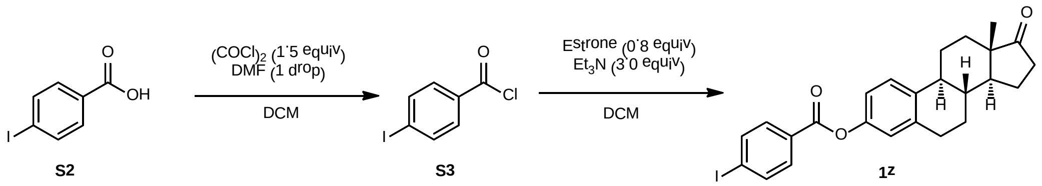


**Supplementary Figure 2.** Preparation of (8*R*,9*S*,13*S*,14*S*)-13-methyl-17-oxo-7,8,9,11,12,13,14,15,16,17-decahydro-6*H*-cyclopenta[*a*]phenanthren-3-yl 4-iodobenzoate (**1z**).

**(8*R*,9*S*,13*S*,14*S*)-13-methyl-17-oxo-7,8,9,11,12,13,14,15,16,17-decahydro-6*H*-cyclopenta[*a*]phenanthren-3-yl 4-iodobenzoate (1z).** An oven-dried round-flask with a rubber septum and a magnetic stir bar was charged with 4-Iodobenzoic acid (**34**) (5.0 mmol) and oxalyl chloride ($(COCl)_2$, 7.5 mmol) in anhydrous DCM (6.0 mL) under a positive pressure of nitrogen gas. After addition of one drop of DMF, the mixture was refluxed at 50 °C for 3 hours. Then, the mixture was cooled down to room temperature, concentrated under reduced pressure to give the crude acyl chloride, which was used in the next step without further purification. The residue was dissolved in DCM (5.0 mL) again. Then, iodobenzoyl chloride solution was added dropwise to a solution of Estrone (1.0 g, 4.0 mmol) and $Et_3N$ (1.1 mL, 15.0 mmol) in anhydrous DCM (10.0 mL) placed in another oven-dried round-flask under a positive pressure of nitrogen gas at ice bath. The mixture was allowed to room temperature and stirred at room temperature for 2.5 hours. The solution was

diluted with 10% HCl aq. (4.0 mL) and stirred until separation. After separation, the aqueous layer was extracted with DCM (2 × 20 mL). The combined organic layer was washed with $Na_2CO_3$ aq. (10 mL) and brine (10 mL), dried over anhydrous $Na_2SO_4$, filtered and concentrated under reduced pressure. The residue was purified by silica gel flash chromatography (*n*-Hexane: AcOEt = 9:1) to afford the title product (1.6 g, 80% yield) as a white solid. **$^1$H NMR** (300 MHz, $CDCl_3$) δ: 7.91 – 7.83 (m, 4H), 7.33 (d, *J* = 8.4 Hz, 1H), 6.99 – 6.92 (m, 2H), 2.96 – 2.91 (m, 2H), 2.56 – 2.40 (m, 2H), 2.35 – 2.27 (m, 1H), 2.21 – 1.95 (m, 4H), 1.70 – 1.43 (m, 6H), 0.92 (s, 3H) ppm. **$^{13}$C NMR** (126 MHz, $CDCl_3$) δ: 165.0, 148.6, 138.1, 137.9, 137.6, 131.5, 129.1, 126.5, 121.6, 118.7, 101.5, 50.4, 47.9, 44.1, 38.0, 35.8, 31.5, 29.4, 26.3, 25.8, 21.6, 13.8 ppm. **HRMS** (TOF/EI$^+$): Calculated for $C_{25}H_{25}IO_3^+$: 500.0848, found: 500.0861. **IR** (NaCl): 2931, 2859, 1726, 1583, 1452, 1390, 1371, 1261, 1068, 1006, 897, 843, 783, 577, cm$^{-1}$. **m.p.**: 244.8 – 245.7 °C.

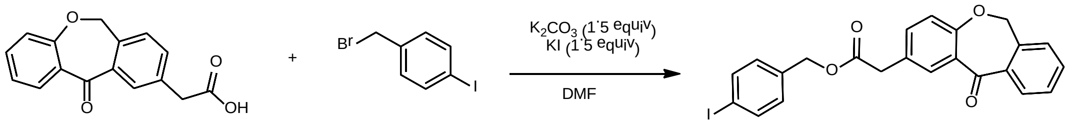


S4 S5 1za

**Supplementary Figure 3.** Preparation of 4-iodobenzyl 2-(11-oxo-6,11-dihydrodibenzo[*b*,*e*]oxepin-2-yl)acetate (**1za**).

**4-iodobenzyl 2-(11-oxo-6,11-dihydrodibenzo[*b*,*e*]oxepin-2-yl)acetate (1za).** An oven-dried round-flask with a rubber septum and a magnetic stir bar was charged with Isoxepac (**S4**) (537 mg, 2.0 mmol), potassium carbonate ($K_2CO_3$) (415 mg, 3.0 mmol, 1.5 equiv), potassium iodide (KI) (498 mg, 3.0 mmol, 1.5 equiv) and anhydrous DMF (20.0 mL) at under a positive pressure of nitrogen gas. A solution of 4-iodobenzyl bromide (**S5**) (653mg, 2.2 mmol, 1.1 equiv) dissolved in anhydrous DMF (10.0 mL) was added dropwise to the mixture and stirred for 24 hours at room temperature. Then reaction mixture was diluted with water and extracted with diethyl ether (20 mL ×3). The organic layers were washed with water (10 mL×3) and brine (10 mL), dried over anhydrous $Na_2SO_4$, filtered and concentrated under reduced pressure. The residue was purified by silica gel flash chromatography (*n*-Hexane: AcOEt = 5:1) and recrystallization (*n*-Hexane: AcOEt) to afford the title product (875 mg, 90% yield) as a white solid. **$^1$H NMR** (300 MHz, $CDCl_3$) δ: 8.12 (d, *J* = 2.4 Hz, 1H), 7.89 (dd, *J* = 7.6, 1.5 Hz, 1H), 7.70 – 7.66 (m, 2H), 7.57 (td, *J* = 7.4, 1.5 Hz, 1H), 7.48 (td, *J* = 7.5, 1.4 Hz, 1H), 7.43 – 7.36 (m, 2H), 7.10 – 7.01 (m, 3H), 5.20 (d, *J* = 2.7 Hz, 2H), 5.07 (s, 2H), 3.68 (s, 2H) ppm. **$^{13}$C NMR** (126 MHz, $CDCl_3$) δ: 190.8, 171.1, 160.5, 140.4, 137.6, 136.3, 135.4, 135.3, 132.8, 132.4, 130.0, 129.4, 129.3, 127.8, 127.4, 125.1, 121.1, 94.0, 73.6, 66.0, 40.1 ppm. **HRMS** (ESI): Calcd. for $C_{23}H_{17}INaO_4$ [M+Na]$^+$ : 507.0064. Found: 507.0067. **IR** (NaCl): 2359, 2341, 1736, 1647, 1610, 1487, 1454, 1300, 1203, 1140, 1007, 831, 798, 760, 561 cm$^{-1}$. **m.p.**: 88.8 – 89.8 °C.

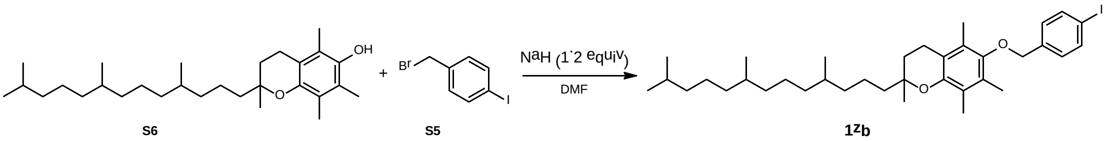


**Supplementary Figure 4.** Preparation of

(all-*rac*)-6-((4-Iodobenzyl)oxy)-2,5,7,8-tetramethyl-2-(4,8,12-trimethyltridecyl)chroman (**1zb**).

**(all-*rac*)-6-((4-Iodobenzyl)oxy)-2,5,7,8-tetramethyl-2-(4,8,12-trimethyltridecyl)chroman (21zb).** An oven-dried round-flask with a rubber septum and a magnetic stir bar was charged with (±)-α-tocopherol (**S6**) (1.36 g, 3.0 mmol) and anhydrous DMF (5.0mL) at under a positive pressure of nitrogen gas. Sodium hydride (NaH) (60% in oil, 144 mg, 3.6 mmol, 1.2 equiv) was added to the mixture, and the mixture was stirred for 10 min. Then, 4-iodobenzyl bromide (**S5**) (980 mg, 3.3 mmol, 1.1 equiv) was added to the mixture, and the mixture was heated to 80 °C and stirring was continued for 24 h. After the reaction mixture was cooled down to room temperature, AcOEt (10 mL) was added to the mixture and the mixture was filtered through a Celite pad and washed with AcOEt (5 mL). The filtrate was washed with brine (10 mL×3), dried over anhydrous $Na_2SO_4$, filtered and concentrated under reduced pressure. The residue was purified by silica gel flash chromatography (*n*-Hexane: AcOEt = 30:1) to afford the title product (1.8 g, 93% yield) as a colorless viscous oil. **$^1$H NMR** (300 MHz, $CDCl_3$) δ: 7.72 – 7.69 (m, 2H), 7.23 – 7.22 (m, 2H), 4.63 (s, 2H), 2.58 (t, *J* = 6.8 Hz, 2H), 2.18 (s, 3H), 2.13 (s, 3H), 2.10 (s, 3H), 1.82 (dt, *J* = 13.9, 7.1 Hz, 1H), 1.76 (dt, *J* = 13.3, 6.5 Hz, 1H), 1.60 – 1.05 (m, 24H), 0.87 – 0.84 (m, 12H) ppm. **$^{13}$C NMR** (126 MHz, $CDCl_3$) δ: 148.0, 147.9, 137.7, 137.5, 129.4, 127.8, 125.8, 123.0, 117.6, 93.1, 74.8, 73.8, 40.01, 39.96, 39.4, 37.6, 37.54, 37.47, 37.44, 37.39, 37.37, 37.34, 37.32, 37.27, 32.78, 32.78, 32.67, 32.65, 31.3, 31.2, 28.0, 24.81, 24.80, 24.4, 23.9, 22.7, 22.6, 21.03, 21.01, 21.00, 20.7, 19.8, 19.69, 19.66, 19.63, 19.60, 12.9, 12.0, 11.8 ppm. **HRMS** (TOF/EI$^+$): Calculated for $C_{36}H_{55}IO_2^+$: 646.3247, found: 646.3254. **IR** (NaCl): 2925, 2865, 1482, 1460, 1415, 1389, 1375, 1253, 1159, 1088, 1061, 1006, 798, 731, 476 cm$^{-1}$.

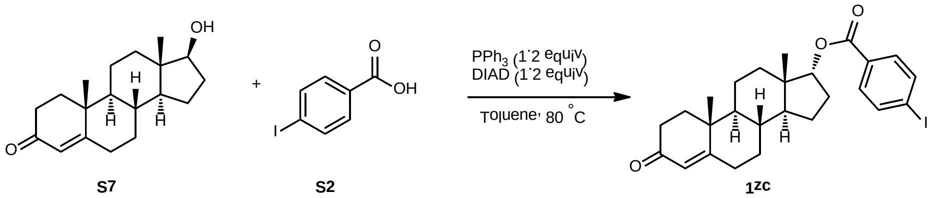


**Supplementary Figure 5.** Preparation of

(8*R*,9*S*,10*R*,13*S*,14*S*,17*R*)-10,13-dimethyl-3-oxo-2,3,6,7,8,9,10,11,12,13,14,15,16,17-tetradecahydro-1*H*-cyclopenta[*a*]phenanthren-17-yl 4-iodobenzoate (**1zc**).

**(8*R*,9*S*,10*R*,13*S*,14*S*,17*R*)-10,13-dimethyl-3-oxo-2,3,6,7,8,9,10,11,12,13,14,15,16,17-tetradecahydro-1*H*-cyclopenta[*a*]phenanthren-17-yl 4-iodobenzoate (1zc).** An oven-dried round-flask with a rubber septum and a magnetic stir bar was charged with testosterone (**S7**) (1.0 g, 3.5 mmol), 4-iodobenzoic acid (**S2**) (1.2 g,

4.9 mmol, 1.4 equiv), triphenylphosphine ($PPh_3$, 1.3 g, 4.9 mmol, 1.4 equiv), Diisopropyl azodicarboxylate (DIAD, 1.0 g 4.9 mmol, 1.4 equiv) and anhydrous toluene (30 mL) at under a positive pressure of nitrogen gas. The mixture was heated stirred at 50 °C for 4 hours. After the reaction mixture was cooled down to room temperature, then the mixture was concentrated under reduced pressure. The residue was purified by silica gel flash chromatography (*n*-Hexane: AcOEt: DCM: Toluene = 7:1:1:2) to afford the title product (1.55g 86% yield) as a white solid. **$^1$H NMR** (300 MHz, $CDCl_3$) δ: 7.81 – 7.70 (m, 4H), 5.75 (d, *J* = 1.7 Hz, 1H), 5.06 (d, *J* = 6.2 Hz, 1H), 2.44 – 2.27 (m, 5H), 2.03 (ddd, *J* = 13.4, 5.0, 3.2 Hz, 1H), 1.96 – 1.85 (m, 2H), 1.83 – 1.44 (m, 9H), 1.39 – 1.31 (m, 1H), 1.20 (s, 3H), 1.04 – 0.97 (m, 1H), 0.87 (s, 3H) ppm. **$^{13}$C NMR** (126 MHz, $CDCl_3$) δ: 199.4, 170.9, 165.5, 137.6, 130.9, 130.0, 123.9, 100.5, 82.3, 53.4, 49.9, 44.9, 38.6, 35.7, 35.6, 33.9, 32.8, 32.1, 31.8, 30.0, 24.6, 20.4, 17.4, 16.5 ppm. **HRMS** (ESI): Calcd. for $C_{26}H_{31}INaO_3$ $[M+Na]^+$ : 541.1210. Found: 541.1205. **IR** (NaCl): 2943, 2879, 1815, 1720, 1674, 1238, 1182, 1109, 779, 723, 505 cm$^{-1}$. **m.p.**: 142.6 – 143.6 °C.

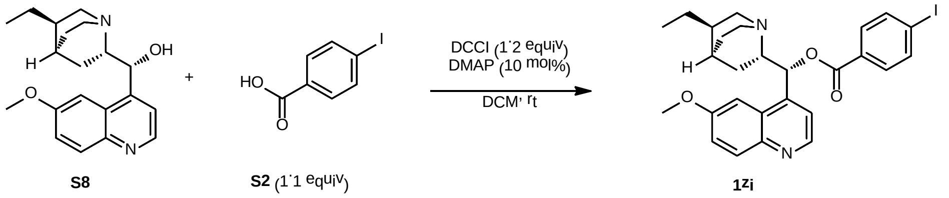


**Supplementary Figure 6.** Preparation of (1*R*)-((2*S*,4*S*,5*R*)-5-ethylquinuclidin-2-yl)(6-methoxyquinolin-4-yl)methyl 4-iodobenzoate (1zi).

**(1*R*)-((2*S*,4*S*,5*R*)-5-ethylquinuclidin-2-yl)(6-methoxyquinolin-4-yl)methyl 4-iodobenzoate (1zi).** An oven-dried round-flask with a rubber septum and a magnetic stir bar was charged with hydroquinine (**S8**, 1.63g, 5.0 mmol), *N*,*N'*-dicyclohexylcarbodiimide (DCCI, 6.0 mmol, 1.2 equiv), 4-Dimethylaminopyridine (DMAP, 0.5 mmol, 10 mol%) and anhydrous DCM (25 ml) at under a positive pressure of nitrogen gas. Then, the mixtures were cooled down to 0 °C with an ice bath 10 min, 4-iodobenzoic acid (**S2**, 5.5 mmol, 1.1 equiv) was added to the mixture. When the additions were completed, the reaction mixture was allowed to stir at room temperature for 24 hours. After the reaction was complete (monitored by TLC), the *N*,*N'*-dicyclohexylurea was filtered off and the filtrate was washed with $H_2O$ (3 × 30 mL), dried over anhydrous $Na_2SO_4$, filtered and concentrated under reduced pressure. The residue was purified by silica gel flash chromatography (AcOEt) to afford the title product (2.03g, 73% yield) as a white solid. **$^1$H NMR** (300 MHz, $CDCl_3$) δ: 8.72 (d, *J* = 4.4 Hz, 1H), 8.02 (d, *J* = 9.2 Hz, 1H), 7.86 – 7.78 (m, 4H), 7.49 (t, *J* = 2.2 Hz, 1H), 7.42 – 7.36 (m, 2H), 6.72 (d, *J* = 6.5 Hz, 1H), 3.98 (s, 3H), 3.50 – 3.44 (m, 1H), 3.17 – 3.02 (m, 2H), 2.74 – 2.60 (m, 1H), 2.40 –2.34 (m, 1H), 1.94 – 1.62 (m, 4H), 1.51 – 1.30 (m, 4H), 0.86 (t, *J* = 7.2, 3H) ppm. **$^{13}$C NMR** (126 MHz, $CDCl_3$) δ: 165.1, 158.0, 147.4, 144.8, 143.5, 138.0, 131.9, 131.0, 129.2, 126.9, 121.9,

118.5, 101.4, 101.3, 74.9, 59.2, 58.5, 55.7, 42.7, 37.4, 28.6, 27.8, 25.3, 23.9, 12.1 ppm. **HRMS** (ESI): Calcd. for $C_{27}H_{30}IN_2O_3$ $[M+H]^+$ : 557.1301. Found: 557.1287. **IR** (NaCl): 2933, 2870, 1724, 1658, 1619, 1601, 1537, 1508, 1441, 1321, 1267, 1103, 1032, 1018, 910, 754, 729, 692, 586 $cm^{-1}$. **m.p.**: 191.4 – 192.2 °C.

**Typical Procedure Preparation of 2-(Difluoromethoxy)-5-Nitropyridine (2).**

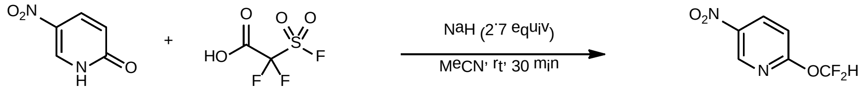


**Supplementary Figure 7.** Preparation of 2-(Difluoromethoxy)-5-Nitropyridine (**2**).

**2-(Difluoromethoxy)-5-Nitropyridine (2).** **2** is prepared in accordance with a reported procedure.[8] An oven-dried round-flask with a rubber septum and a magnetic stir bar was charged with 2-hydroxy-5-nitro-pyridine (**S9**, 0.7 g, 5 mmol) and anhydrous acetonitrile (50 mL) under a positive pressure of nitrogen gas. Then NaH (540 mg, 13.5 mmol, 60% in mineral oil) was added to the mixture and stirring for 15 min at room temperature. 2,2-difluoro-2-(fluorosulfonyl)acetic acid (**S10**, 0.88 mL, 8.5 mmol) was added dropwise over several minutes. After stirring the reaction mixture at room temperature for 15 min, the reaction was quenched by the slow addition of water (10 mL). The acetonitrile was removed *in vacuo* and the residue was extracted with AcOEt, washed with brine, dried over anhydrous $Na_2SO_4$, filtered and concentrated under reduced pressure. The residue was purified by silica gel flash chromatography (*n*-Hexane: AcOEt = 9:1) to afford **22** (83-90% yield) as a white solid. **$^1$H NMR** (300 MHz, $CDCl_3$) δ: 9.10 (d, *J* = 2.8 Hz, 1H), 8.54 (dd, *J* = 9.0, 2.8 Hz, 1H), 7.53 (t, *J* = 71.6 Hz, 1H), 7.05 (d, *J* = 9.0 Hz, 1H) ppm. **$^{19}$F NMR** (282 MHz, $CDCl_3$) δ: -90.12 (d, *J* = 72.8 Hz, 2F) ppm. **$^{13}$C NMR** (126 MHz, $CDCl_3$) δ: 162.0 (t, *J* = 4.0 Hz), 144.2, 141.6, 135.5, 113.7 (t, *J* = 258.9 Hz), 111.8 ppm. **HRMS** (TOF/EI$^+$): Calculated for $C_6H_4F_2N_2O_3^+$: 190.0190, found: 190.0189. **IR** (NaCl): 1527, 1346, 1346, 1268, 1134, 1076 $cm^{-1}$. **m.p.**: 31.8 – 32.7 °C.

**General Procedure and Product Characterization data for acyl fluorides 3 and 4.**

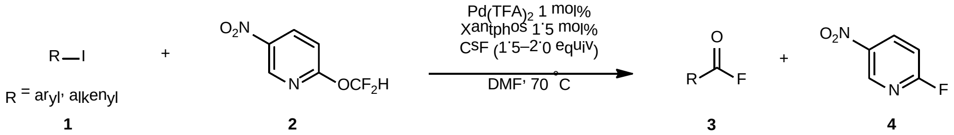


**Supplementary Figure 8.** General Procedure for acyl fluorides **3** and **4.**

**General procedure A**:

An oven-dried vessel containing a magnetic stir bar was charged with $Pd(TFA)_2$ (1.0 mg, 0.003 mmol, 1.0 mol %), Xantphos (2.6 mg, 0.0045 mmol, 1.5 mol %), CsF (68.4 mg, 0.45 mmol, 1.5 equiv) and anhydrous *N*,*N*-dimethylformamide (DMF, 2.0 mL, 0.15 M) in a nitrogen-filled glovebox. After stirring the reaction mixture at room temperature for 10 min, then **2** (0.36 mmol, 1.2 equiv) and aryl or alkenyl iodides **1** (0.3 mmol, 1.0 equiv) were added to the reaction mixture. The vessel was capped with a rubber septum and

moved from the glovebox then stirred at 70 °C for 15 hours. Then, the mixtures were cooled down to room temperature, yields were determined by $^{19}F$ NMR analysis of the crude products using $C_6H_5F$ (28.5 μL, 0.3 mmol, 1.0 equiv) as an internal standard. The crude mixture was directly purified by silica gel flash chromatography (10 cm thick × 2 cm diameter), to afford the desired compound **3**.

**General procedure B**:

An oven-dried vessel containing a magnetic stir bar was charged with $Pd(TFA)_2$ (1.0 mg, 0.003 mmol, 1.0 mol %), Xantphos (2.6 mg, 0.0045 mmol, 1.5 mol %), CsF (91.1 mg, 0.6 mmol, 1.5 equiv) and anhydrous *N*,*N*-dimethylformamide (DMF, 2.0 mL, 0.15 M) in a nitrogen-filled glovebox. After stirring the reaction mixture at room temperature for 10 min, then **2** (0.45 mmol, 1.5 equiv) and aryl or alkenyl iodides **1** (0.3 mmol, 1.0 equiv) were added to the reaction mixture. The vessel was capped with a rubber septum and moved from the glovebox then stirred at 70 °C for 15 hours. Then, the mixtures were cooled down to room temperature, yields were determined by $^{19}F$ NMR analysis of the crude products using $C_6H_5F$ (28.5 μL, 0.3 mmol, 1.0 equiv) as an internal standard. The crude mixture was directly purified by silica gel flash chromatography (10 cm thick × 2 cm diameter), to afford the desired compound **3**.

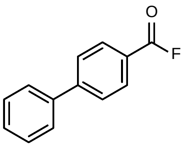


**3a**

**[1,1'-Biphenyl]-4-carbonyl fluoride (3a).** Following the general procedure **A**, using substrate **1a** (0.3 mmol, 1.0 equiv) and **2** (0.36 mmol, 1.2 equiv) in DMF (2.0 mL), the reaction mixture was stirred at 70 °C for 15 hours. The yield (>99%) was determined by $^{19}F$ NMR of the crude reaction mixture. The crude mixture was purified by column chromatography (*n*-Hexane: AcOEt = 50:1) to afford the title compound **3a** (55.3 mg, 92% yield) as a white solid. **$^1$H NMR** (300 MHz, $CDCl_3$) δ: 8.13 – 8.10 (m, 2H), 7.76 – 7.73 (m, 2H), 7.65 – 7.63 (m, 2H), 7.53 – 7.41 (m, 3H) ppm.**$^{19}$F NMR** (282 MHz, $CDCl_3$) δ: 17.65 (s, 1F) ppm. **MS** (EI): *m/z* 200$[M]^+$. Spectroscopic data was agreement with the literature. [9]

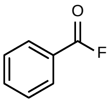


**3b**

**Benzoyl fluoride (3b).** Following the general procedure **B**, using substrate **1b** (0.3 mmol, 1.0 equiv) and **2** (0.45 mmol, 1.5 equiv) in DMF (2.0 mL), the reaction mixture was stirred at 70 °C for 15 hours. The yield (92%) was determined by $^{19}F$ NMR of the crude reaction mixture. The crude mixture was purified by column chromatography (pentane: $Et_2O$ = 50:1) to afford the title compound **3b** (10.1 mg, 27% yield) as a colorless

oil. **$^1$H NMR** (300 MHz, $CDCl_3$) δ: 8.05 (d, *J* = 7.7 Hz, 2H), 7.71 (t, *J* = 8.0 Hz, 1H), 7.58 – 7.46 (m, 2H) ppm. **$^{19}$F NMR** (282 MHz, $CDCl_3$) δ: 17.63 (s, 1F) ppm. **MS** (EI): *m/z* 124 $[M]^+$. Spectroscopic data was agreement with the literature. [10]

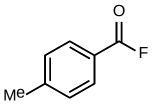

**3c**

**4-Methylbenzoyl fluoride (3c).** Following the general procedure **A**, using substrate **1c** (0.3 mmol, 1.0 equiv) and **2** (0.36 mmol, 1.2 equiv) in DMF (2.0 mL), the reaction mixture was stirred at 70 °C for 15 hours. The yield (92%) was determined by $^{19}$F NMR of the crude reaction mixture. The crude mixture was purified by column chromatography (*n*-Hexane: AcOEt = 50:1) to afford the title compound **3c** (31.9 mg, 77% yield) as a colorless oil. **$^1$H NMR** (300 MHz, $CDCl_3$) δ: 7.93 (d, *J* = 7.8 Hz, 2H), 7.32 (d, *J* = 7.8 Hz, 2H), 2.45 (s, 3H) ppm.**$^{19}$F NMR** (282 MHz, $CDCl_3$) δ: 16.96 (s, 1F) ppm. **MS** (EI): *m/z* 138 $[M]^+$. Spectroscopic data was agreement with the literature. [11]

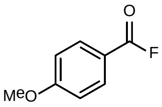

**3d**

**4-Methoxybenzoyl fluoride (3d).** Following the general procedure **A**, using substrate **1d** (0.3 mmol, 1.0 equiv) and **2** (0.36 mmol, 1.2 equiv) in DMF (2.0 mL), the reaction mixture was stirred at 70 °C for 15 hours. The yield (92%) was determined by $^{19}$F NMR of the crude reaction mixture. The crude mixture was purified by column chromatography (*n*-Hexane: AcOEt = 50:1) to afford the title compound **3d** (37.4 mg, 81% yield) as a colorless oil. **$^1$H NMR** (300 MHz, $CDCl_3$) δ: 7.99 (d, *J* = 6.9 Hz, 2H), 6.98 (d, *J* = 8.3 Hz, 2H), 3.89 (s, 3H) ppm.**$^{19}$F NMR** (282 MHz, $CDCl_3$) δ: 15.49 (s, 1F) ppm. **MS** (EI): *m/z* 154 $[M]^+$. Spectroscopic data was agreement with the literature. [12]

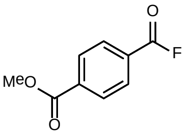

**3e**

**Methyl 4-(fluorocarbonyl)benzoate (3e).** Following the general procedure **A**, using substrate **1e** (0.3 mmol, 1.0 equiv) and **2** (0.36 mmol, 1.2 equiv) in DMF (2.0 mL), the reaction mixture was stirred at 70 °C for 15 hours. The yield (93%) was determined by $^{19}$F NMR of the crude reaction mixture. The crude mixture was purified by column chromatography (*n*-Hexane: AcOEt = 25:1) to afford the title compound **3e** (43.7 mg,

80% yield) as a white solid. **$^1$H NMR** (300 MHz, $CDCl_3$) δ: 8.20 – 8.11 (m, 4H), 3.98 (s, 3H) ppm. **$^{19}$F NMR** (282 MHz, $CDCl_3$) δ: 19.61 (s, 1F) ppm. **MS** (EI): *m/z* 182 $[M]^+$. Spectroscopic data was agreement with the literature. [9]

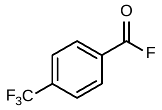

**3f**

**4-(Trifluoromethyl)benzoyl fluoride (3f).** Following the general procedure **A**, using substrate **1f** (0.3 mmol, 1.0 equiv) and **2** (0.36 mmol, 1.2 equiv) in DMF (2.0 mL), the reaction mixture was stirred at 70 °C for 15 hours. The yield (92%) was determined by $^{19}$F NMR of the crude reaction mixture. The crude mixture was purified by column chromatography (Pentane: $Et_2O$ = 25:1) to afford the title compound **3f** (14.4 mg, 25% yield) as a colorless oil. **$^1$H NMR** (300 MHz, $CDCl_3$) δ: 8.19 (d, $J$ = 8.1 Hz, 2H), 7.82 (d, $J$ = 8.1 Hz, 2H) ppm. **$^{19}$F NMR** (282 MHz, $CDCl_3$) δ: 19.51 (s, 1F), -63.97 (s, 3F) ppm. **MS** (EI): *m/z* 192 $[M]^+$. Spectroscopic data was agreement with the literature.[13]

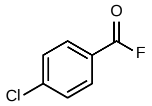

**3g**

**4-Chlorobenzoyl fluoride (3g).** Following the general procedure **B**, using substrate **1g** (0.3 mmol, 1.0 equiv) and **2** (0.45 mmol, 1.5 equiv) in DMF (2.0 mL), the reaction mixture was stirred at 70 °C for 15 hours. The yield (94%) was determined by $^{19}$F NMR of the crude reaction mixture. The crude mixture was purified by column chromatography (*n*-Hexane: AcOEt = 25:1) to afford the title compound **3g** (32.3 mg, 68% yield) as a pale yellow solid. **$^1$H NMR** (300 MHz, $CDCl_3$) δ: 8.01 – 7.97 (m, 2H), 7.53 – 7.50 (m, 2H) ppm.**$^{19}$F NMR** (282 MHz, $CDCl_3$) δ: 17.95 (s, 1F) ppm. **MS** (EI): *m/z* 157 $[M]^+$. Spectroscopic data was agreement with the literature. [9]

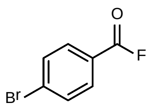

**3h**

**4-Bromobenzoyl fluoride (3h).** Following the general procedure **B**, using substrate **1h** (0.3 mmol, 1.0 equiv) and **2** (0.45 mmol, 1.5 equiv) in DMF (2.0 mL), the reaction mixture was stirred at 70 °C for 15 hours. The yield (73%) was determined by $^{19}$F NMR of the crude reaction mixture. The crude mixture was purified

by column chromatography ($n$-Hexane: AcOEt = 50:1) to afford the title compound **3h** (41.3 mg, 68% yield) as a white solid. **$^1$H NMR** (300 MHz, $CDCl_3$) δ: 7.91 (d, $J$ = 7.8 Hz, 1H), 7.69 (d, $J$ = 8.1 Hz, 1H) ppm. **$^{19}$F NMR** (282 MHz, $CDCl_3$) δ: 17.93 (s, 1F) ppm. **MS** (EI): $m/z$ 202 $[M]^+$. Spectroscopic data was agreement with the literature. [12]

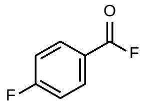

**3i**

**4-Fluorobenzoyl fluoride (3i).** Following the general procedure **A**, using substrate **1i** (0.3 mmol, 1.0 equiv) and **2** (0.36 mmol, 1.2 equiv) in DMF (2.0 mL), the reaction mixture was stirred at 70 °C for 15 hours. The yield (>99%) was determined by $^{19}$F NMR of the crude reaction mixture. The crude mixture was purified by column chromatography (pentane: $Et_2O$ = 25:1) to afford the title compound **3i** (21.3 mg, 50% yield) as a colorless oil. **$^1$H NMR** (300 MHz, $CDCl_3$) δ: 8.11 – 8.07 (m, 1H), 7.25 – 7.19 (m, 1H) ppm. **$^{19}$F NMR** (282 MHz, $CDCl_3$) δ: 17.54 (s, 1F), -100.99 – -101.05 (m, 1F) ppm. **MS** (EI): $m/z$ 142 $[M]^+$. Spectroscopic data was agreement with the literature. [11]

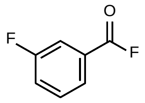

**3j**

**3-Fluorobenzoyl fluoride (3j).** Following the general procedure **A**, using substrate **1j** (0.3 mmol, 1.0 equiv) and **2** (0.36 mmol, 1.2 equiv) in DMF (2.0 mL), the reaction mixture was stirred at 70 °C for 15 hours. The yield (86%) was determined by $^{19}$F NMR of the crude reaction mixture. The crude mixture was purified by column chromatography (pentane: $Et_2O$ = 50:1) to afford the title compound **3j** (14.1 mg, 33% yield) as a colorless oil. **$^1$H NMR** (300 MHz, $CDCl_3$) δ: 7.87 (d, $J$ = 7.7 Hz, 1H), 7.75 – 7.71 (m, 1H), 7.57 – 7.50 (m, 1H), 7.46 – 7.39 (m, 1H) ppm. **$^{19}$F NMR** (282 MHz, $CDCl_3$) δ: 18.90 (d, $J$ = 4.5 Hz, 1F), -110.00 – -111.06 (m, 1F) ppm. **MS** (EI): $m/z$ 142 $[M]^+$. Spectroscopic data was agreement with the literature. [11]

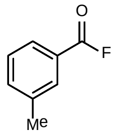

**3k**

**3-Methylbenzoyl fluoride (3k).** Following the general procedure **B**, using substrate **1k** (0.3 mmol, 1.0 equiv) and **2** (0.45 mmol, 1.5 equiv) in DMF (2.0 mL), the reaction mixture was stirred at 70 °C for 15 hours.

The yield (74%) was determined by $^{19}$F NMR of the crude reaction mixture. The crude mixture was purified by column chromatography (*n*-Hexane: AcOEt = 50:1) to afford the title compound **3k** (26.1 mg, 63% yield) as a colorless oil. **$^1$H NMR** (300 MHz, $CDCl_3$) δ: 7.87 – 7.85 (m, 2H), 7.51 (d, 1H), 7.42 (t, 1H) ppm. **$^{19}$F NMR** (282 MHz, $CDCl_3$) δ: 17.77 (s, 1F) ppm. **MS** (EI): *m/z* 138 $[M]^+$. Spectroscopic data was agreement with the literature. [11]

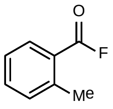


**3l**

**2-Methylbenzoyl fluoride (3l).** Following the general procedure **A**, using substrate **1l** (0.3 mmol, 1.0 equiv) and **2** (0.36 mmol, 1.2 equiv) in DMF (2.0 mL), the reaction mixture was stirred at 70 °C for 15 hours. The yield (50%) was determined by $^{19}$F NMR of the crude reaction mixture. The crude mixture was purified by column chromatography (*n*-Hexane: AcOEt = 50:1) to afford the title compound **3l** (14.5 mg, 35% yield) as a colorless oil. **$^1$H NMR** (300 MHz, $CDCl_3$) δ: 8.00 (d, *J* = 7.7 Hz, 1H), 7.55 (t, *J* = 7.1 Hz, 1H), 7.34 (d, *J* = 6.3 Hz, 2H), 2.65 (s, 3H) ppm. **$^{19}$F NMR** (282 MHz, $CDCl_3$) δ: 28.77 (s, 1F) ppm. **MS** (EI): *m/z* 138 $[M]^+$. Spectroscopic data was agreement with the literature. [13]

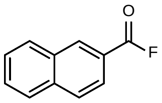


**3m**

**2-Naphthoyl fluoride (3m).** Following the general procedure **A**, using substrate **1m** (0.3 mmol, 1.0 equiv) and **2** (0.36 mmol, 1.2 equiv) in DMF (2.0 mL), the reaction mixture was stirred at 70 °C for 15 hours. The yield (>99%) was determined by $^{19}$F NMR of the crude reaction mixture. The crude mixture was purified by column chromatography (*n*-Hexane: AcOEt = 50:1) to afford the title compound **3m** (46.5 mg, 89% yield) as a white solid. **$^1$H NMR** (300 MHz, $CDCl_3$) δ: 8.62 (s, 1H), 8.01 – 7.90 (m, 4H), 7.70 – 7.58 (m, 2H) ppm. **$^{19}$F NMR** (282 MHz, $CDCl_3$) δ: 17.56 (s, 1F) ppm. **MS** (EI): *m/z* 174 $[M]^+$. Spectroscopic data was agreement with the literature. [9]

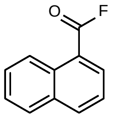


**3n**

**1-Naphthoyl fluoride (3n).** Following the general procedure **A**, using substrate **1n** (0.3 mmol, 1.0 equiv) and **2** (0.36 mmol, 1.2 equiv) in DMF (2.0 mL), the reaction mixture was stirred at 70 °C for 15 hours. The

yield (95%) was determined by $^{19}$F NMR of the crude reaction mixture. The crude mixture was purified by column chromatography (*n*-Hexane: AcOEt = 50:1) to afford the title compound **3n** (45.5 mg, 87% yield) as a white solid. **$^1$H NMR** (300 MHz, $CDCl_3$) δ: 9.01 (d, J = 8.7 Hz, 1H), 8.34 (d, J = 7.4 Hz, 1H), 8.16 (d, J = 8.2 Hz, 1H), 7.93 (d, J = 8.2 Hz, 1H), 7.70 (t, *J* = 7.4 Hz, 1H), 7.62 – 7.52 (m, 2H) ppm. **$^{19}$F NMR** (282 MHz, $CDCl_3$) δ: 29.38 (s, 1F) ppm. **MS** (EI): *m/z* 174 $[M]^+$. Spectroscopic data was agreement with the literature. [14]

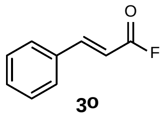

3o

**Cinnamoyl fluoride (3o).** Following the general procedure **A**, using substrate **1o** (0.3 mmol, 1.0 equiv) and **2** (0.36 mmol, 1.2 equiv) in DMF (2.0 mL), the reaction mixture was stirred at 70 °C for 15 hours. The yield (93%) was determined by $^{19}$F NMR of the crude reaction mixture. The crude mixture was purified by column chromatography (*n*-Hexane: AcOEt = 50:1) to afford the title compound **3o** (38.6 mg, 86% yield) as a colorless oil. **$^1$H NMR** (300 MHz, $CDCl_3$) δ: 7.84 (d, *J* = 16.0 Hz, 1H), 7.58 – 7.55 (m, 2H), 7.51 – 7.41 (m, 3H), 6.37 (dd, *J* = 16.0, 7.4 Hz, 1H) ppm. **$^{19}$F NMR** (282 MHz, $CDCl_3$) δ: 25.10 (d, *J* = 7.4 Hz, 1F) ppm. **MS** (EI): *m/z* 150 $[M]^+$. Spectroscopic data was agreement with the literature.[12]

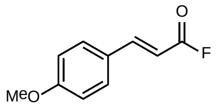

3p

***(E)*-3-(4-Methoxyphenyl)acryloyl fluoride (3p).** Following the general procedure **A**, using substrate **1p** (0.3 mmol, 1.0 equiv) and **2** (0.36 mmol, 1.2 equiv) in DMF (2.0 mL), the reaction mixture was stirred at 70 °C for 15 hours. The yield (97%) was determined by $^{19}$F NMR of the crude reaction mixture. The crude mixture was purified by column chromatography (*n*-Hexane: AcOEt = 25:1) to afford the title compound **3p** (49.2 mg, 91% yield) as a white solid. **$^1$H NMR** (300 MHz, $CDCl_3$) δ: 7.78 (d, *J* = 15.9 Hz, 1H), 7.52 (d, *J* = 8.3 Hz, 2H), 6.94 (d, *J* = 8.7 Hz, 2H), 6.21 (dd, *J* = 15.9, 7.4 Hz, 1H), 3.86 (s, 3H) ppm. **$^{19}$F NMR** (282 MHz, $CDCl_3$) δ: 23.91 (d, *J* = 7.6 Hz, 1F) ppm. **MS** (EI): *m/z* 180 $[M]^+$. Spectroscopic data was agreement with the literature. [15]

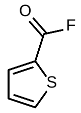

3q

**Thiophene-2-carbonyl fluoride (3q).** Following the general procedure **B**, using substrate **1q** (0.3 mmol, 1.0

equiv) and **2** (0.45 mmol, 1.5 equiv) in DMF (2.0 mL), the reaction mixture was stirred at 70 °C for 15 hours. The yield (87%) was determined by $^{19}$F NMR of the crude reaction mixture. The crude mixture was purified by column chromatography (pentane: $Et_2O$ = 25:1) to afford the title compound **3q** (25.8 mg, 66% yield) as a colorless oil. **$^1$H NMR** (300 MHz, $CDCl_3$) δ: 7.95 – 7.92 (m, 1H), 7.82 – 7.79 (m, 1H), 7.23 – 7.19 (m, 1H) ppm. **$^{19}$F NMR** (282 MHz, $CDCl_3$) δ: 23.88 (s, 1F) ppm. **MS** (EI): *m/z* 130 $[M]^+$. Spectroscopic data was agreement with the literature.[16]

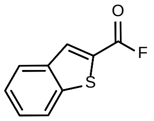

**3r**

**Benzo[*b*]thiophene-2-carbonyl fluoride (3r).** Following the general procedure **B**, using substrate **1r** (0.3 mmol, 1.0 equiv) and **2** (0.45 mmol, 1.5 equiv) in DMF (2.0 mL), the reaction mixture was stirred at 70 °C for 15 hours. The yield (70%) was determined by $^{19}$F NMR of the crude reaction mixture. The crude mixture was purified by column chromatography (*n*-Hexane: AcOEt = 50:1) to afford the title compound **3r** (33.5 mg, 62% yield) as a colorless oil. **$^1$H NMR** (300 MHz, $CDCl_3$) δ: 8.21 (s, 1H), 7.96 – 7.89 (m, 2H), 7.58 – 7.45 (m, 2H) ppm. **$^{19}$F NMR** (282 MHz, $CDCl_3$) δ: 24.58 (s, 1F) ppm. **MS** (EI): *m/z* 180 $[M]^+$. Spectroscopic data was agreement with the literature.[17]

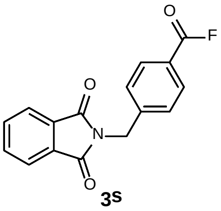

**3s**

**4-((1,3-Dioxoisoindolin-2-yl)methyl)benzoyl fluoride (3s).** Following the general procedure **A**, using substrate **1s** (0.3 mmol, 1.0 equiv) and **2** (0.36 mmol, 1.2 equiv) in DMF (2.0 mL), the reaction mixture was stirred at 70 °C for 15 hours. The yield (92%) was determined by $^{19}$F NMR of the crude reaction mixture. The crude mixture was purified by column chromatography (*n*-Hexane: AcOEt = 3:1) to afford the title compound **3s** (63.4 mg, 75% yield) as a pale yellow solid. **$^1$H NMR** (300 MHz, $CDCl_3$) δ: 8.03 – 7.99 (m, 2H), 7.90 – 7.86 (m, 2H), 7.78 – 7.74 (m, 2H), 7.59 – 7.55 (m, 2H), 4.93 (s, 2H) ppm. **$^{19}$F NMR** (282 MHz, $CDCl_3$) δ: 17.63 (s, 1F) ppm. **$^{13}$C NMR** (126 MHz, $CDCl_3$) δ: 167.7, 156.9 (d, $J$ = 344.1 Hz), 143.9, 134.2, 131.83, 131.79, 128.9, 124.3 (d, $J$ = 61.4 Hz), 123.5, 41.1 ppm. **HRMS** (TOF/EI$^+$): Calculated for $C_{16}H_{10}FNO_3^+$: 283.0645, found: 283.0638. **IR** (NaCl): 1805, 1718, 1610, 1429, 1392, 1333, 1292, 1242, 1186, 1105, 1088, 1028, 1007, 943, 781, 723, 586 cm$^{-1}$. **m.p.**: 152.5 – 153.5 °C.

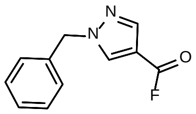

**3t**

**1-Benzyl-1*H*-pyrazole-4-carbonyl fluoride (3t).** Following the general procedure **A**, using substrate **1t** (0.3 mmol, 1.0 equiv) and **2** (0.36 mmol, 1.2 equiv) in DMF (2.0 mL), the reaction mixture was stirred at 70 °C for 15 hours. The yield (59%) was determined by $^{19}$F NMR of the crude reaction mixture. The crude mixture was purified by column chromatography (*n*-Hexane: AcOEt = 10:1) to afford the title compound **3t** (35.0 mg, 57% yield) as a white solid. **$^1$H NMR** (300 MHz, $CDCl_3$) δ: 8.01 (s, 1H), 7.94 (s, 1H), 7.40 – 7.37 (m, 3H), 7.29 – 7.27 (m, 2H), 5.33 (s, 2H) ppm. **$^{19}$F NMR** (282 MHz, $CDCl_3$) δ: 26.09 (s, 1F) ppm. **$^{13}$C NMR** (126 MHz, $CDCl_3$) δ: 152.8 (d, *J* = 329.4 Hz), 142.7 (d, *J* = 3.6 Hz), 134.6 (d, *J* = 3.8 Hz), 134.3, 129.2, 128.9, 128.2, 109.3 (d, *J* = 73.8 Hz), 56.8 ppm. **HRMS** (TOF/EI$^+$): Calculated for $C_{11}H_9FN_2O^+$: 204.0699, found: 204.0692. **IR** (NaCl): 1807, 1547, 1496, 1455, 1392, 1360, 1205, 1173, 1063, 989, 955, 879, 748, 729, 704, 609, 517, 496 cm$^{-1}$. **m.p.**: 43.9 – 45.1 °C.

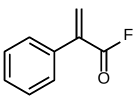

**3u**

**2-Phenylacryloyl fluoride (3u).** Following the general procedure **A**, using substrate **1u** (0.3 mmol, 1.0 equiv) and **2** (0.36 mmol, 1.2 equiv) in DMF (2.0 mL), the reaction mixture was stirred at 70 °C for 15 hours. The yield (38%) was determined by $^{19}$F NMR of the crude reaction mixture. The crude mixture was purified by column chromatography (pentane: $Et_2O$ = 25:1) to afford the title compound **3u** (13.5 mg, 30% yield) as a colorless oil. (Note: unstable to be stored for long period and have high volatility) **$^1$H NMR** (500 MHz, $CDCl_3$) δ: 7.47 – 7.45 (m, 2H), 7.41 – 7.39 (m, 2H), 6.60 (s, 1H), 6.28 (d, *J* = 1.5 Hz, 1H) ppm. **$^{19}$F NMR** (282 MHz, $CDCl_3$) δ: 22.47 (s, 1F) ppm. **$^{13}$C NMR** (126 MHz, $CDCl_3$) δ: 156.3 (d, *J* = 350.6 Hz), 136.7 (d, *J* = 56.3 Hz), 134.4 (d, *J* = 3.3 Hz), 133.0, 129.1, 128.5, 128.1 (d, *J* = 1.8 Hz) ppm. **HRMS** (TOF/EI$^+$): Calculated for $C_9H_7FO^+$: 150.0481, found: 150.0482. **IR** (NaCl): 1811, 1275, 1261, 750, 480, 463, 440, 428, 413, 401 cm$^{-1}$.

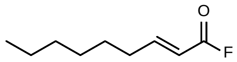

**3v**

***(E)*-Non-2-enoyl fluoride (3v).** Following the general procedure **A**, using substrate **1v** (0.3 mmol, 1.0 equiv) and **2** (0.36 mmol, 1.2 equiv) in DMF (2.0 mL), the reaction mixture was stirred at 70 °C for 15 hours. The

yield (20%) was determined by $^{19}$F NMR of the crude reaction mixture. The crude mixture was purified by column chromatography (pentane: $Et_2O$ = 50:1) to afford the title compound **3v** (6.2 mg, 13% yield) as a colorless oil. (Note: unstable to be stored for long period and have high volatility) **$^1$H NMR** (300 MHz, $CDCl_3$) δ: 7.19 (dd, *J* = 15.6, 7.0 Hz, 1H), 5.80 (ddd, *J* = 15.7, 8.4, 1.7 Hz, 1H), 2.28 (qd, *J* = 7.2, 1.6 Hz, 2H), 1.49 (dd, *J* = 10.2, 4.5 Hz, 2H), 1.33 – 1.28 (m, 6H), 0.89 (t, *J* = 6.0 Hz, 3H) ppm. **$^{19}$F NMR** (282 MHz, $CDCl_3$) δ: 24.48 (d, *J* = 8.5 Hz, 1F) ppm. **$^{13}$C NMR** (126 MHz, $CDCl_3$) δ: 157.8 (d, *J* = 5.7 Hz), 156.5 (d, *J* = 340.7 Hz), 116.0 (d, *J* = 64.9 Hz), 32.7, 31.5, 28.8, 27.5, 22.5, 14.0 ppm. **HRMS** (TOF/EI$^+$): Calculated for $C_9H_{15}FO^+$: 158.1107, found: 158.1104. **IR** (NaCl): 3901, 3869, 3820, 3749, 3647, 2925, 2854, 1811, 1649, 1541, 669, 577, 490, 442 cm$^{-1}$**.**

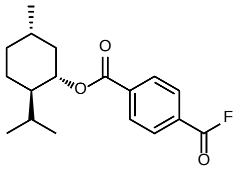

3w

**(*1S,2R,5S*)-2-Isopropyl-5-methylcyclohexyl 4-(fluorocarbonyl)benzoate (3w).** Following the general procedure **B**, using substrate **1w** (0.3 mmol, 1.0 equiv) and **2** (0.45 mmol, 1.5 equiv) in DMF (2.0 mL), the reaction mixture was stirred at 70 °C for 15 hours. The yield (84%) was determined by $^{19}$F NMR of the crude reaction mixture. The crude mixture was purified by column chromatography (*n*-Hexane: $Et_2O$ = 19:1) to afford the title compound **3w** (32.5 mg, 35% yield) as a colorless viscous oil. **$^1$H NMR** (300 MHz, $CDCl_3$) δ: 8.20 – 8.10 (m, 4H), 4.98 (ddd, *J* = 10.9, 4.3 Hz, 1H), 2.16 – 2.08 (m, 1H), 1.97 – 1.88 (m, 1H), 1.78 – 1.69 (m, 2H), 1.63 – 1.51 (m, 2H), 1.20 – 1.10 (m, 3H), 0.98 – 0.90 (m, 6H), 0.80 (d, *J* = 7.0 Hz, 3H) ppm. **$^{19}$F NMR** (282 MHz, $CDCl_3$) δ: 19.53 (s, 1F) ppm. **$^{13}$C NMR** (126 MHz, $CDCl_3$) δ: 164.6, 156.6 (d, *J* = 345.7 Hz), 136.7, 131.3 (d, *J* = 3.5 Hz), 130.0, 128.3 (d, *J* = 61.5 Hz), 75.9, 47.2, 40.8, 34.2, 31.4, 26.5, 23.6, 22.0, 20.7, 16.5 ppm. **HRMS** (TOF/EI$^+$): Calculated for $C_{18}H_{23}FO_3^+$: 306.1631, found: 306.1624. **IR** (NaCl): 2956, 2871, 1819, 1720, 1238, 1107, 1034, 1011, 982, 779, 721, 519, 503, 455 cm$^{-1}$.

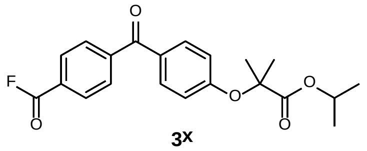

3x

**Isopropyl 2-(4-(4-(fluorocarbonyl)benzoyl)phenoxy)-2-methylpropanoate (3x).** Following the general procedure **B**, using substrate **1x** (0.3 mmol, 1.0 equiv) and **2** (0.45 mmol, 1.5 equiv) in DMF (2.0 mL), the reaction mixture was stirred at 70 °C for 15 hours. The yield (88%) was determined by $^{19}$F NMR of the crude

reaction mixture. The crude mixture was purified by column chromatography (*n*-Hexane: AcOEt = 10:1) and recrystallization (*n*-Hexane: DCM = 19:1) to afford the title compound **3x** (44.7 mg, 40% yield) as a white solid. **$^1$H NMR** (300 MHz, $CDCl_3$) δ: 8.16 (d, *J* = 8.3 Hz, 2H), 7.85 (d, *J* = 8.0 Hz, 2H), 7.76 (d, *J* = 8.6 Hz, 2H), 6.89 – 6.86 (m, 2H), 5.09 (septet, *J* = 6.3 Hz, 1H), 1.67 (s, 6H), 1.21 (d, *J* = 6.3 Hz, 6H) ppm. **$^{19}$F NMR** (282 MHz, $CDCl_3$) δ: 19.28 (s, 1F) ppm. **$^{13}$C NMR** (126 MHz, $CDCl_3$) δ: 194.0, 172.9, 160.3, 156.6 (d, *J* = 345.2 Hz), 144.2, 132.1, 131.3 (d, *J* = 3.5 Hz), 129.8, 129.3, 127.3 (d, *J* = 61.7 Hz), 79.5, 69.4, 25.3, 21.5 ppm. **HRMS** (TOF/EI$^+$): Calculated for $C_{21}H_{21}FO_5^+$: 372.1373, found: 372.1373. **IR** (NaCl): 2986, 2939, 1815, 1730, 1650, 1597, 1284, 1250, 1178, 1146, 1103, 1031, 1009, 930, 704, 488, 461, 449, 440 cm$^{-1}$. **m.p.**: 107.3 – 108.4 °C.

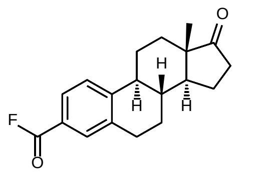


**3y**

**(8*R*,9*S*,13*S*,14*S*)-13-Methyl-17-oxo-7,8,9,11,12,13,14,15,16,17-decahydro-6*H*-cyclopenta[*a*]phenanthrene-3-carbonyl fluoride (3y).** Following the general procedure **A**, using substrate **1y** (0.3 mmol, 1.0 equiv) and **2** (0.36 mmol, 1.2 equiv) in DMF (2.0 mL), the reaction mixture was stirred at 70 °C for 15 hours. The yield (65%) was determined by $^{19}$F NMR of the crude reaction mixture. The crude mixture was purified by column chromatography (*n*-Hexane: AcOEt = 3:1) to afford the title compound **3y** (51.2 mg, 57% yield) as a white solid. **$^1$H NMR** (300 MHz, $CDCl_3$) δ: 7.82 – 7.77 (m, 2H), 7.44 (d, J = 8.1 Hz, 1H), 3.01 – 2.95 (m, 2H), 2.58 – 2.43 (m, 2H), 2.41 – 2.33 (m, 1H), 2.23 – 2.14 (m, 1H), 2.12 – 1.98 (m, 3H), 1.70 – 1.45 (m, 6H), 0.93 (s, 3H) ppm. **$^{19}$F NMR** (282 MHz, $CDCl_3$) δ: 17.30 (s, 1F) ppm. **$^{13}$C NMR** (126 MHz, $CDCl_3$) δ: 157.5 (d, *J* = 343.2 Hz), 148.0, 137.6, 132.0 (d, *J* = 3.8 Hz), 128.6 (d, *J* = 3.5 Hz), 126.1, 122.1 (d, *J* = 60.5 Hz), 50.4, 47.8, 44.7, 37.5, 35.7, 31.4, 29.0, 26.0, 25.4, 21.5, 13.7 ppm. **HRMS** (TOF/EI$^+$): Calculated for $C_{19}H_{21}FO_2^+$: 300.1526, found: 300.1532. **IR** (NaCl): 2927, 2860, 1801, 1735, 1606, 1241, 1086, 1032, 1009, 787, 739, 490, 480, 465, 449 cm$^{-1}$. **m.p.**: 206.5 – 207.6 °C.

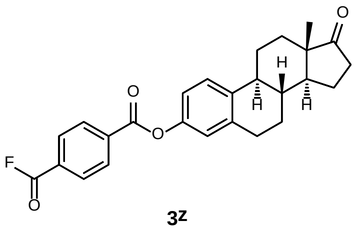


**3z**

**(8*R*,9*S*,13*S*,14*S*)-13-Methyl-17-oxo-7,8,9,11,12,13,14,15,16,17-decahydro-6*H*-cyclopenta[*a*]phenanthren-3-yl 4-(fluorocarbonyl)benzoate (3z).** Following the general procedure **A**, using substrate **1z** (0.3 mmol,

1.0 equiv) and **2** (0.36 mmol, 1.2 equiv) in DMF (2.0 mL), the reaction mixture was stirred at 70 °C for 15 hours. The yield (67%) was determined by $^{19}$F NMR of the crude reaction mixture. The crude mixture was purified by column chromatography (*n*-Hexane: AcOEt = 3:1) to afford the title compound **3z** (36.6 mg, 29% yield) as a white solid. **$^{1}$H NMR** (300 MHz, $CDCl_3$) δ: 8.34 (d, *J* = 8.7 Hz, 2H), 8.20 – 8.17 (m, 2H), 7.36 (d, *J* = 8.4 Hz, 1H), 7.02 – 6.96 (m, 2H), 2.97 – 2.93 (m, 2H), 2.57 – 2.41 (m, 2H), 2.33 (td, *J* = 10.4, 4.0 Hz, 1H), 2.19 – 2.16 (m, 1H), 2.11 – 1.96 (m, 3H), 1.70 – 1.44 (m, 6H), 0.93 (s, 3H) ppm. **$^{19}$F NMR** (282 MHz, $CDCl_3$) δ: 19.86 (s, 1F) ppm. **$^{13}$C NMR** (126 MHz, $CDCl_3$) δ: 164.0, 156.4 (d, *J* = 345.8 Hz), 148.4, 138.3, 138.0, 135.5, 131.5 (d, *J* = 3.1 Hz), 130.6, 129.1 (d, *J* = 61.9 Hz), 126.6, 121.4, 118.6, 50.4, 47.9, 44.2, 38.0, 35.8, 31.5, 29.4, 26.3, 25.8, 21.6, 13.8 ppm. **HRMS** (TOF/EI$^+$): Calculated for $C_{26}H_{25}FO_4^+$: 420.1737 found: 420.1725. **IR** (NaCl): 2931, 2870, 1813, 1738, 1493, 1410, 1265, 1238, 1149, 1074, 1032, 1009, 897, 714, 540, 476 cm$^{-1}$. **m.p.**: 198.1 – 199.3 °C.

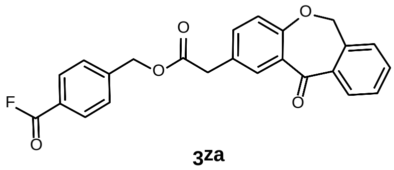


**3za**

**4-(Fluorocarbonyl)benzyl 2-(11-oxo-6,11-dihydrodibenzo[*b*,*e*]oxepin-2-yl)acetate (3za).** Following the general procedure **A**, using substrate **1za** (0.3 mmol, 1.0 equiv) and **2** (0.36 mmol, 1.2 equiv) in DMF (2.0 mL), the reaction mixture was stirred at 70 °C for 15 hours. The yield (87%) was determined by $^{19}$F NMR of the crude reaction mixture. The crude mixture was purified by column chromatography (*n*-Hexane: AcOEt = 3:1) to afford the title compound **3za** (76.2 mg, 63% yield) as a pale yellow solid. **$^{1}$H NMR** (300 MHz, $CDCl_3$) δ: 8.15 (d, *J* = 2.3 Hz, 1H), 8.02 (d, *J* = 8.0 Hz, 2H), 7.88 (dd, *J* = 7.6, 1.5 Hz, 1H), 7.57 (td, *J* = 7.4, 1.6 Hz, 1H), 7.50 – 7.41 (m, 4H), 7.37 (dd, *J* = 7.4, 1.4 Hz, 1H), 7.04 (d, *J* = 8.4 Hz, 1H), 5.22 (s, 2H), 5.19 (s, 2H), 3.73 (s, 2H) ppm. **$^{19}$F NMR** (282 MHz, $CDCl_3$) δ: 18.01 (s, 1F) ppm. **$^{13}$C NMR** (126 MHz, $CDCl_3$) δ: 190.7, 170.9, 160.5, 157.0 (d, *J* = 344.1 Hz), 143.5, 140.3, 136.2, 135.4, 132.8, 132.4, 131.6 (d, *J* = 3.9 Hz), 129.4, 129.3, 128.0, 127.8, 127.2, 124.5 (d, *J* = 61.4 Hz), 124.2, 121.1, 73.6, 65.5, 40.0 ppm. **HRMS** (TOF/EI$^+$): Calculated for $C_{24}H_{17}FO_5^+$: 404.1060, found: 404.1068. **IR** (NaCl): 1807, 1739, 1647, 1612, 1489, 1414, 1300, 1242, 1140, 1120, 1032, 1009, 827, 760, 741, 692, 640, 457 cm$^{-1}$. **m.p.**: 107.7 – 108.6 °C.

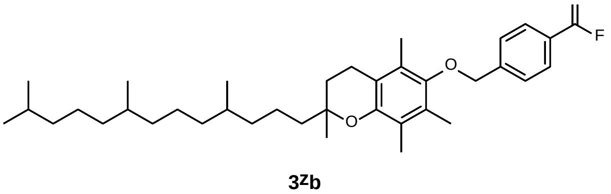


**3zb**

**(All-*rac*)-4-(((2,5,7,8-tetramethyl-2-(4,8,12-trimethyltridecyl)chroman-6-yl)oxy)methyl)benzoyl fluoride (3zb).** Following the general procedure **A**, using substrate **1zb** (0.3 mmol, 1.0 equiv) and **2** (0.36 mmol, 1.2 equiv) in DMF (2.0 mL), the reaction mixture was stirred at 70 °C for 15 hours. The yield (93%) was determined by $^{19}$F NMR of the crude reaction mixture. The crude mixture was purified by column chromatography (*n*-Hexane: AcOEt = 30:1) to afford the title compound **3zb** (126.8 mg, 75% yield) as a colorless viscous oil. **$^{1}$H NMR** (300 MHz, $CDCl_3$) δ: 8.07 (d, *J* = 7.7 Hz, 2H), 7.65 (d, *J* = 7.9 Hz, 2H), 4.80 (s, 2H), 2.59 (s, 2H), 2.19 (s, 3H), 2.14 (s, 3H), 2.11 (s, 3H), 1.80 (q, *J* = 7.4 Hz, 2H), 1.58 – 1.06 (m, 24H), 0.88 – 0.84 (m, 12H) ppm. **$^{19}$F NMR** (282 MHz, $CDCl_3$) δ: 17.68 (s, 1F) ppm. **$^{13}$C NMR** (126 MHz, $CDCl_3$) δ: 157.2 (d, *J* = 343.7 Hz), 148.1, 147.7, 146.2, 131.6 (d, *J* = 3.8 Hz), 127.7, 127.4, 127.3, 125.7, 124.0 (d, *J* = 60.9 Hz), 123.1, 117.7, 73.4, 40.0, 39.9, 39.4, 37.6, 37.6, 37.54, 37.47, 37.44, 37.40, 37.37, 37.31, 37.27, 32.78, 32.76, 32.67, 32.65, 31.24, 31.19, 28.0, 24.81, 24.80, 24.4, 23.9, 22.7, 22.62, 22.59, 21.03, 21.02, 21.02, 20.7, 19.74, 19.69, 19.67, 19.65, 19.62, 19.59, 12.8, 11.9, 11.8 ppm. **HRMS** (TOF/EI$^{+}$): Calculated for $C_{37}H_{55}FO_3^{+}$: 566.4135, found: 566.4158. **IR** (NaCl): 2927, 2868, 1813, 1612, 1460, 1409, 1375, 1254, 1174, 1090, 1032, 1011, 741, 507 cm$^{-1}$.

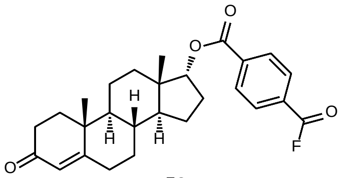


**3zc**

**(8*R*,9*S*,10*R*,13*S*,14*S*,17*R*)-10,13-Dimethyl-3-oxo-2,3,6,7,8,9,10,11,12,13,14,15,16,17-tetradecahydro-1*H*-cyclopenta[*a*]phenanthren-17-yl 4-(fluorocarbonyl)benzoate (3zc).** Following the general procedure **A**, using substrate **1zc** (0.3 mmol, 1.0 equiv) and **2** (0.36 mmol, 1.2 equiv) in DMF (2.0 mL), the reaction mixture was stirred at 70 °C for 15 hours. The yield (61%) was determined by $^{19}$F NMR of the crude reaction mixture. The crude mixture was purified by column chromatography (*n*-Hexane: AcOEt = 3:1) to afford the title compound **3zc** (19.7 mg, 15% yield) as a white solid. **$^{1}$H NMR** (300 MHz, $CDCl_3$) δ: 8.17 – 8.10 (m, 4H), 5.75 (d, *J* = 1.8 Hz, 1H), 5.11 (d, *J* = 6.2 Hz, 1H), 2.50 – 2.28 (m, 5H), 2.08 – 1.85 (m, 3H), 1.84 – 1.27 (m, 10H), 1.21 (s, 3H), 1.10 – 0.92 (m, 1H), 0.89 (s, 3H) ppm. **$^{19}$F NMR** (282 MHz, $CDCl_3$) δ: 19.61 (s, 1F) ppm. **$^{13}$C NMR** (126 MHz, $CDCl_3$) δ: 199.4, 170.8, 164.6, 156.5 (d, *J* = 345.6 Hz), 136.5, 131.4 (d, *J* = 3.4 Hz), 130.0, 128.5 (d, *J* = 61.5 Hz), 123.9, 83.2, 53.5, 50.0, 45.0, 38.6, 35.8, 35.7, 33.9, 32.8, 32.2, 31.9, 30.1, 24.7, 20.4, 17.4, 16.6 ppm. **HRMS** (TOF/EI$^{+}$): Calculated for $C_{27}H_{31}FO_4^{+}$: 438.2206 found: 438.2221. **IR** (NaCl): 2943, 2879, 1814, 1720, 1674, 1410, 1277, 1238, 1107, 1032, 1011, 870, 721, 511, 494 cm$^{-1}$. **m.p.**: 80.6 – 81.8 °C.

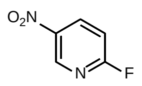

4

**2-Fluoro-5-nitropyridine (4)** **$^{1}$H NMR** (300 MHz, $CDCl_3$) δ: 9.13 (s, 1H), 8.65 – 8.59 (m, 1H), 7.15 – 7.11 (m, 1H) ppm. **$^{19}$F NMR** (282 MHz, $CDCl_3$) δ: 57.67 (s, 1F) ppm. Spectroscopic data was agreement with the literature. [18]

**Gram-Scale Synthesis of 3a.**

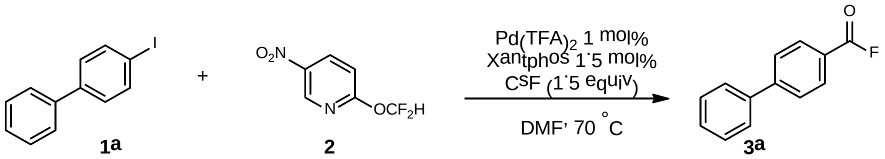


**Supplementary Figure 9.** Gram-scale synthesis of **3a**.

An oven-dried 100-mL round-bottomed flask with a rubber septum and magnetic stir bar was charged with $Pd(TFA)_2$ (15.0 mg, 0.045 mmol, 1.0 mol %), Xantphos (39.1 mg, 0.0675 mmol, 1.5 mol %) CsF (1.03 g, 6.75 mmol, 1.5 equiv) and anhydrous *N*,*N*-Dimethylformamide (30.0 mL, 0.15 M) in a nitrogen-filled glovebox. After stirring the reaction mixture at room temperature for 10 min, then **2** (1.03 g, 5.4 mmol, 1.2 equiv) and 4-iodo biphenyl **1a** (1.26 g, 4.5 mmol, 1.0 equiv) were added to the reaction mixture. The vessel was capped with a rubber septum and moved from the glovebox then stirred at 70 °C for 15 hours. Then, the mixtures were cooled down to room temperature, yields (>99%) were determined by $^{19}$F NMR analysis of the crude products using $C_6H_5F$ (0.43 mL, 4.5 mmol, 1.0 equiv) as an internal standard. After the solvent was removed by a rotary evaporator, the crude residue was purified by silica gel flash chromatography (10 cm thick × 4 cm diameter, *n*-Hexane: AcOEt = 50:1), to afford the titled compound **3a** (812.8 mg, 90% yield) as a white solid.

**Synthetic Application I (Supplementary Figure 10-17).**

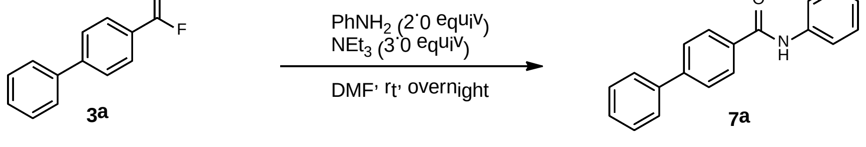


**Supplementary Figure 10.** Transformation of **3a** to amide **7a**.

***N*-Phenyl-[1,1'-biphenyl]-4-carboxamide(7a).** An oven-dried vessel containing a magnetic stir bar was charged with **3a** (20.0 mg, 0.1 mmol) and anhydrous DMF (1.0 mL, 0.1 M) under a positive pressure of nitrogen gas. Then, $NEt_3$ (42 μL, 0.3 mmol, 3.0 equiv) and $PhNH_2$ (18 μL, 0.2 mmol, 2.0 equiv) were added and the mixture was allowed stirred overnight at rt. After the reaction was complete (monitored by TLC),

$H_2O$ (10 mL) was added and the mixture extracted with AcOEt (3 × 10 mL). The combined organic phase was dried over anhydrous $Na_2SO_4$, filtered and concentrated under reduced pressure. The crude residue was purified via silica gel flash chromatography (*n*-Hexane: AcOEt = 1:1) to afford the title compound **7a** (25.9 mg, 95% yield) as a white solid. **$^1$H NMR** (300 MHz, DMSO-$d_6$) δ: 10.29 (s, 1H), 8.06 (d, *J* = 8.4 Hz, 2H), 7.85 – 7.74 (m, 6H), 7.54 – 7.33 (m, 5H), 7.11 (t, *J* = 7.3 Hz, 1H) ppm. **MS** (ESI): *m/z* 272 $[M-H]^-$. Spectroscopic data was agreement with the literature. [19]

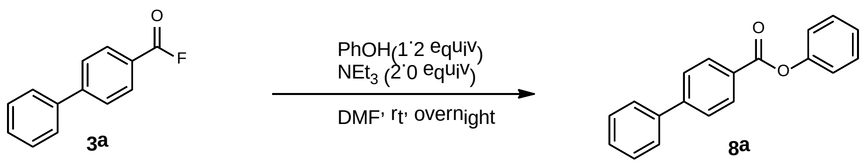


**Supplementary Figure 11.** Transformation of **3a** to ester **8a**.

**Phenyl [1,1'-biphenyl]-4-carboxylate (8a)**. An oven-dried vessel containing a magnetic stir bar was charged with **3a** (20.0 mg, 0.1 mmol), Phenol (PhOH) (11.3 mg, 0.12 mmol, 1.2 equiv) and anhydrous DMF (1.0 mL, 0.1 M) under a positive pressure of nitrogen gas. Then, $NEt_3$ (28 μL, 0.2 mmol, 2.0 equiv) were added and the mixture was allowed stirred overnight at rt. After the reaction was complete (monitored by TLC), $H_2O$ (10 mL) was added and the mixture extracted with AcOEt (3 × 10 mL). The combined organic phase was dried over anhydrous $Na_2SO_4$, filtered and concentrated under reduced pressure. The crude residue was purified via silica gel flash chromatography (*n*-Hexane: Chloroform = 1:5) to afford the title compound **8a** (23.4 mg, 85% yield) as a white solid. **$^1$H NMR** (300 MHz, $CDCl_3$) δ: 8.28 (d, *J* = 8.5 Hz, 2H), 7.74 (d, *J* = 8.5 Hz, 2H), 7.66 (dd, *J* = 7.1, 1.6 Hz, 2H), 7.52 – 7.39 (m, 5H), 7.31 – 7.22 (m, 3H) ppm. **MS** (ESI): *m/z* 275 $[M+H]^+$. Spectroscopic data was agreement with the literature. [20]

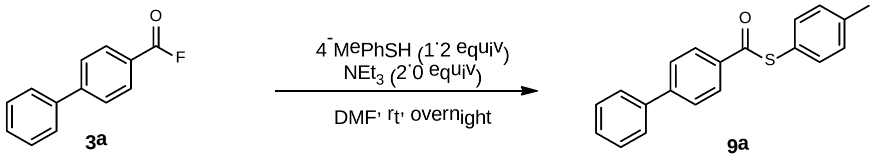


**Supplementary Figure 12.** Transformation of **3a** to thioester **9a**.

***S*-*p*-Tolyl [1,1'-biphenyl]-4-carbothioate (9a).** An oven-dried vessel containing a magnetic stir bar was charged with **3a** (20.0 mg, 0.1 mmol), *p*-Toluenethiol (4-MePhSH) (14.9 mg, 0.12 mmol, 1.2 equiv) and anhydrous DMF (1.0 mL, 0.1 M) under a positive pressure of nitrogen gas. Then, $NEt_3$ (28 μL, 0.2 mmol, 2.0 equiv) were added and the mixture was allowed stirred overnight at rt. After the reaction was complete (monitored by TLC), $H_2O$ (10 mL) was added and the mixture extracted with AcOEt (3 × 10 mL). The combined organic phase was dried over anhydrous $Na_2SO_4$, filtered and concentrated under reduced pressure.

The crude residue was purified via silica gel flash chromatography (*n*-Hexane: AcOEt = 1:10) to afford the title compound **9a** (23.1 mg, 76% yield) as a white solid. **$^1$H NMR** (300 MHz, $CDCl_3$) δ: 8.09 (d, *J* = 8.4 Hz, 2H), 7.69 (d, J = 8.4 Hz, 2H), 7.62 (d, J = 6.9 Hz, 2H), 7.52 – 7.36 (m, 5H), 7.27 (d, *J* = 7.9 Hz, 2H), 2.40 (s, 3H) ppm. **MS** (ESI): *m/z* 305 $[M+H]^+$. Spectroscopic data was agreement with the literature. [21]

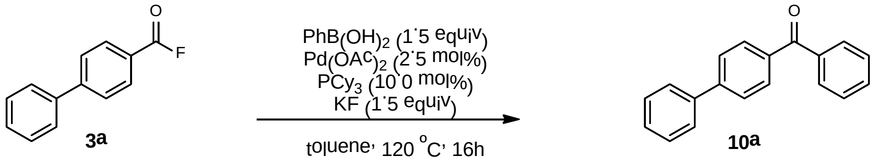


3a 10a

**Supplementary Figure 13.** Transformation of **3a** to ketone compound **10a**

**[1,1'-Biphenyl]-4-yl(phenyl)methanone (10a). 10a** is prepared in accordance with a reported procedure.[22] An oven-dried vessel containing a magnetic stir bar was charged with **3a** (40.0 mg, 0.2 mmol), $Pd(OAc)_2$ (1.1 mg, 0.005 mmol, 2.5 mol %), $PCy_3$ (5.6 mg, 0.02 mmol, 10.0 mol %), KF (17.4 mg, 0.3 mmol, 1.5 equiv) and anhydrous toluene (0.5 mL) in a nitrogen-filled glovebox. The mixture was stirred for 1 min then $PhB(OH)_2$ (0.3 mmol, 1.5 equiv) were added. The vessel was capped with a rubber septum and moved from the glovebox then stirred at 120 °C for 16 hour. Then, $H_2O$ (10 mL) was added and the mixture extracted with AcOEt (3 × 10 mL). The combined organic phase was dried over anhydrous $Na_2SO_4$, filtered and concentrated under reduced pressure. The crude residue was purified via silica gel flash chromatography (*n*-Hexane: $CHCl_3$ = 5:1) to afford the title compound **10a** (24.3 mg, 47% yield) as a white solid. **$^1$H NMR** (300 MHz, $CDCl_3$) δ: 7.90 (d, *J* = 8.0 Hz, 2H), 7.84 (d, *J* = 7.6 Hz, 2H), 7.71 (d, *J* = 7.9 Hz, 2H), 7.66 (d, *J* = 7.7 Hz, 2H), 7.60 (d, *J* = 7.1 Hz, 1H), 7.50 (q, *J* = 7.0 Hz, 4H), 7.43 – 7.39 (m, 1H) ppm. **MS** (ESI): *m/z* 259 $[M+H]^+$. Spectroscopic data was agreement with the literature. [22]

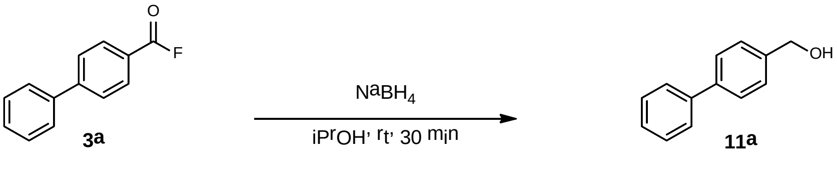


3a 11a

**Supplementary Figure 14.** Transformation of **3a** to alcohol **11a.**

**[1,1'-Biphenyl]-4-ylmethanol (11a).** An oven-dried vessel containing a magnetic stir bar was charged with **3a** (60.0 mg, 0.3 mmol), and *i*PrOH (1.0 mL, 0.1 M). Then, $NaBH_4$ (11.3 mg, 0.3 mmol, 1.0 equiv) were added and the mixture was allowed stirred at rt for 30 min. After the reaction was complete (monitored by TLC), $H_2O$ (10 mL) was added and the mixture extracted with AcOEt (3 × 10 mL). The combined organic phase was dried over anhydrous $Na_2SO_4$, filtered and concentrated under reduced pressure. The crude residue was purified via recrystallization to afford the title compound **11a** (51.4 mg, 93% yield) as a white

solid. **$^1$H NMR** (300 MHz, $CDCl_3$) δ: 7.59 (d, *J* = 7.8 Hz, 4H), 7.46 – 7.37 (m, 4H), 7.34 (q, *J* = 7.3 Hz, 1H), 4.73 (s, 2H), 1.79 (s, 1H) ppm. **MS** (ESI): *m/z* 185 $[M+H]^+$. Spectroscopic data was agreement with the literature.[23]

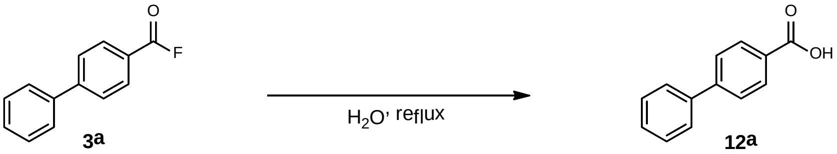


**Supplementary Figure 15.** Transformation of **3a** to Carboxylic Acid **12a**

**[1,1'-Biphenyl]-4-carboxylic acid (12a)**. A vessel containing a magnetic stir bar was charged with **3a** (40.0 mg, 0.2 mmol) and distilled $H_2O$ (1 mL), then the mixture was refluxed for 2 h. The mixture was cooled to room temperature and extracted with AcOEt (3 × 10 mL). The combined organic extracts were extracted with NaOH (1M, 2 × 10 mL), the combined aqueous extracts were acidified to pH2 with HCl (1M) and extracted with AcOEt (3 × 10 mL). The combined organic phase was dried over anhydrous $Na_2SO_4$, filtered and concentrated under reduced pressure. The crude residue was purified via recrystallization to afford the title compound **12a** (25.0 mg, 63% yield) as a white solid. **$^1$H NMR** (300 MHz, $CDCl_3$) δ: 8.21 – 8.17 (m, 2H), 7.72 – 7.63 (m, 4H), 7.49 – 7.41 (m, 3H) ppm. **MS** (ESI): *m/z* 197 $[M-H]^-$. Spectroscopic data was agreement with the literature.[24]

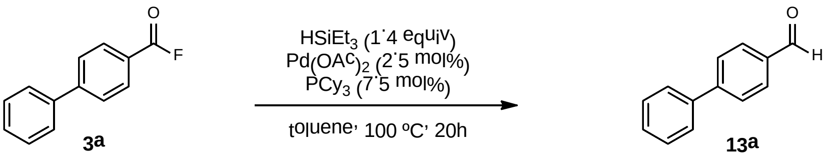


**Supplementary Figure 16.** Transformation of **3a** to aldehyde **13a**

**[1,1'-Biphenyl]-4-carbaldehyde (13a). 13a** is prepared in accordance with a reported procedure.[25] An oven-dried screw-capped vessel containing a magnetic stir bar was charged with **3a** (60.0 mg, 0.3 mmol), $Pd(OAc)_2$ (1.6 mg, 0.0075 mmol, 2.5 mol %), $PCy_3$ (6.3 mg, 0.0225 mmol, 7.5 mol %) and anhydrous toluene (0.3 mL) in a nitrogen-filled glovebox. The mixture was stirred for 1 min then $HSiEt_3$ (0.42 mmol, 1.4 equiv) were added. The vessel was capped and moved from the glovebox then stirred at 100 °C for 20 hour. The crude mixture was purified via silica gel flash chromatography (*n*-Hexane: AcOEt = 1:19) to afford the title compound **13a** (46.7 mg, 85% yield) as a white solid. **$^1$H NMR** (300 MHz, $CDCl_3$) δ: 10.05 (s, 1H), 7.95 (d, *J* = 8.3 Hz, 2H), 7.76 (d, *J* = 8.3 Hz, 2H), 7.65 – 7.62 (m, 2H), 7.51 – 7.39 (m, 3H) ppm. **MS** (ESI): *m/z* 183 $[M+H]^+$. Spectroscopic data was agreement with the literature.[25]

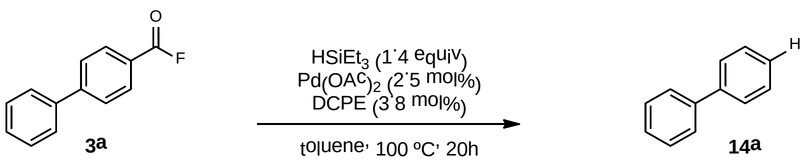


**Supplementary Figure 17.** Decarbonylation of **3a** to aromatic hydrocarbons **14a**

**1,1'-Biphenyl (14a). 14a** is prepared in accordance with a reported procedure.[27] An oven-dried screw-capped vessel containing a magnetic stir bar was charged with **3a** (60.0 mg, 0.3 mmol), $Pd(OAc)_2$ (1.6 mg, 0.0075 mmol, 2.5 mol %), DCPE (4.8 mg, 0.0114 mmol, 3.8 mol %) and anhydrous toluene (0.3 mL) in a nitrogen-filled glovebox. The mixture was stirred for 1 min then $HSiEt_3$ (0.42 mmol, 1.4 equiv) were added. The vessel was capped and moved from the glovebox then stirred at 100 °C for 20 hour. The crude residue was purified via silica gel flash chromatography (*n*-Hexane) to afford the title compound **14a** (30.5 mg, 66% yield) as a white solid. **$^1$H NMR** (300 MHz, $CDCl_3$) δ: 7.62 – 7.57 (m, 4H), 7.48 – 7.41 (m, 4H), 7.38 – 7.31 (m, 2H) ppm. **MS** (EI): *m/z* 154 $[M]^+$. Spectroscopic data was agreement with the literature.[25]

**Synthetic Application II (Supplementary Figure 18-24).**

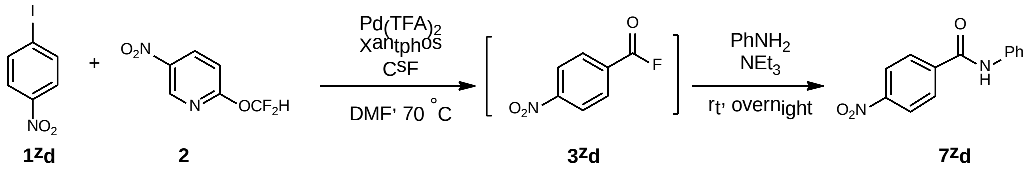


**Supplementary Figure 18.** One-pot amidations of **1zd** to **7zd**.

**4-Nitro-*N*-phenylbenzamide (7zd).** An oven-dried vessel containing a magnetic stir bar was charged with $Pd(TFA)_2$ (1.0 mg, 0.003 mmol, 1.0 mol %), Xantphos (2.6 mg, 0.0045 mmol, 1.5 mol %) CsF (68.4 mg, 0.45 mmol, 1.5 equiv) and anhydrous *N*,*N*-Dimethylformamide (2.0 mL, 0.15 M) in a nitrogen-filled glovebox. After stirring the reaction mixture at room temperature for 10 min, then **2** (0.36 mmol, 1.2 equiv) and aryl iodides **1zd** (0.3 mmol, 1.0 equiv) were added to the reaction mixture. The vessel was capped with a rubber septum and moved from glovebox then stirred at 70 °C for 15 hours. Then, the mixtures were cooled down to room temperature, $NEt_3$ (418 μL, 3.0 mmol, 10.0 equiv) and $PhNH_2$ (81 μL, 0.9 mmol, 3.0 equiv) were added to the reaction mixture and the mixture was stirred at room temperature overnight. After quenching with $H_2O$ (20 mL), the mixture was extracted with AcOEt (3 × 20 mL) and the combined organic layer was dried over anhydrous $Na_2SO_4$, filtered and concentrated under reduced pressure. The crude residue was purified by silica gel flash chromatography (*n*-Hexane: AcOEt = 1:1) to afford the title compound **7zd** (49.4 mg, 68% yield) as a pale yellow solid. **$^1$H NMR** (300 MHz, DMSO-$d_6$) δ: 10.57 (s, 1H), 8.40 – 8.36 (m, 2H), 8.20 – 8.17 (m, 2H), 7.78 (d, *J* = 7.3 Hz, 2H), 7.41 – 7.35 (m, 2H), 7.13 (t, *J* = 8.4 Hz, 1H) ppm. **MS** (ESI): *m/z* 241 $[M-H]^-$. Spectroscopic data was agreement with the literature.[26]

***3zd** NMR yields (76%) were directly determined by $^{19}F$ NMR analysis of the crude products using $C_6H_5F$ (28.5 μL, 0.3 mmol, 1.0 equiv) as an internal standard. **$^{19}$F NMR** (282 MHz, $CDCl_3$) δ: 20.96 (s, 1F) ppm.

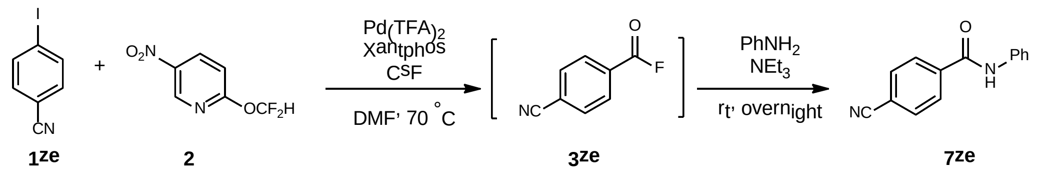


**Supplementary Figure 19.** One-pot amidations of **1ze** to **7ze**.

**4-Cyano-*N*-phenylbenzamide (7ze).** An oven-dried vessel containing a magnetic stir bar was charged with $Pd(TFA)_2$ (1.0 mg, 0.003 mmol, 1.0 mol %), Xantphos (2.6 mg, 0.0045 mmol, 1.5 mol %) CsF (68.4 mg, 0.45 mmol, 1.5 equiv) and anhydrous *N,N*-Dimethylformamide (2.0 mL, 0.15 M) in a nitrogen-filled glovebox. After stirring the reaction mixture at room temperature for 10 min, then **2** (0.36 mmol, 1.2 equiv) and aryl iodides **1ze** (0.3 mmol, 1.0 equiv) were added to the reaction mixture. The vessel was capped with a rubber septum and moved from the glovebox then stirred at 70 °C for 15 hours. Then, the mixtures were cooled down to room temperature, $NEt_3$ (418 μL, 3.0 mmol, 10.0 equiv) and $PhNH_2$ (81 μL, 0.9 mmol, 3.0 equiv) were added to the reaction mixture and the mixture was stirred at room temperature overnight. After quenching with $H_2O$ (20 mL), the mixture was extracted with AcOEt (3 × 20 mL) and the combined organic layer was dried over anhydrous $Na_2SO_4$, filtered and concentrated under reduced pressure. The crude residue was purified by silica gel flash chromatography (*n*-Hexane: AcOEt = 1:1) to afford the title compound **7ze** (61.2 mg, 92% yield) as a colorless oil. **$^1$H NMR** (300 MHz, $CDCl_3$) δ: 7.98 (d, *J* = 8.4 Hz, 2H), 7.90 (s, 1H), 7.78 (d, *J* = 8.3 Hz, 1H), 7.63 (d, *J* = 7.9 Hz, 2H), 7.40 (t, *J* = 8.0 Hz, 2H), 7.20 (t, *J* = 7.4 Hz, 1H) ppm. **MS** (ESI): *m/z* 221 $[M-H]^-$. Spectroscopic data was agreement with the literature.[19]

***3ze** NMR yields (96%) were directly determined by $^{19}F$ NMR analysis of the crude products using $C_6H_5F$ (28.5 μL, 0.3 mmol, 1.0 equiv) as an internal standard. **$^{19}$F NMR** (282 MHz, $CDCl_3$) δ: 19.87 (s, 1F) ppm.

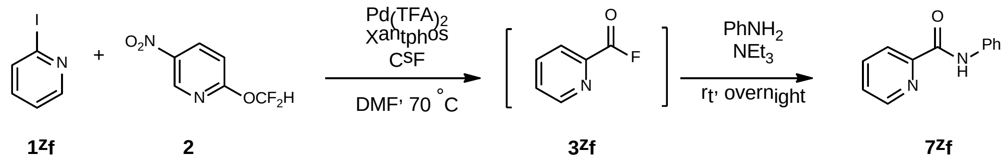


**Supplementary Figure 20.** One-pot amidations of **1zf** to **7zf**.

***N*-Phenylpicolinamide (7zf).** An oven-dried vessel containing a magnetic stir bar was charged with $Pd(TFA)_2$ (1.0 mg, 0.003 mmol, 1.0 mol %), Xantphos (2.6 mg, 0.0045 mmol, 1.5 mol %) CsF (68.4 mg, 0.45 mmol, 1.5 equiv) and anhydrous *N,N*-Dimethylformamide (2.0 mL, 0.15 M) in a nitrogen-filled glovebox. After stirring the reaction mixture at room temperature for 10 min, then **2** (0.36 mmol, 1.2 equiv)

and aryl iodides **1zf** (0.3 mmol, 1.0 equiv) were added to the reaction mixture. The vessel was capped with a rubber septum and moved from the glovebox then stirred at 70 °C for 15 hour. Then, the mixtures were cooled down to room temperature, $NEt_3$ (418 μL, 3.0 mmol, 10.0 equiv) and $PhNH_2$ (81 μL, 0.9 mmol, 3.0 equiv) were added to the reaction mixture and the mixture was stirred at room temperature overnight. After quenching with $H_2O$ (20 mL), the mixture was extracted with AcOEt (3 × 20 mL) and the combined organic layer was dried over anhydrous $Na_2SO_4$, filtered and concentrated under reduced pressure. The crude residue was purified by silica gel flash chromatography (*n*-Hexane: AcOEt = 1:1) to afford the title compound **7zf** (41.6 mg, 70% yield) as a white solid. **$^1$H NMR** (300 MHz, $CDCl_3$) δ: 10.03 (s, 1H), 8.63 – 8.61 (m, 1H), 8.33 – 8.29 (m, 1H), 7.95 – 7.88 (m, 1H), 7.81 – 7.77 (m, 2H), 7.51 – 7.46 (m, 1H), 7.44 – 7.36 (m, 2H), 7.18 – 7.12 (m, 1H) ppm. **MS** (ESI): *m/z* 199 $[M+H]^+$. Spectroscopic data was agreement with the literature.[19]

***3zf** NMR yields (76%) were directly determined by $^{19}$F NMR analysis of the crude products using $C_6H_5F$ (28.5 μL, 0.3 mmol, 1.0 equiv) as an internal standard. **$^{19}$F NMR** (282 MHz, $CDCl_3$) δ: 16.54 (s, 1F) ppm.

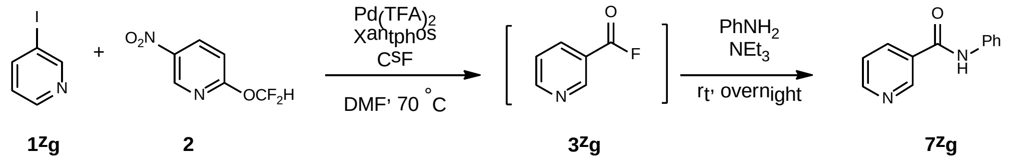


**Supplementary Figure 21.** One-pot amidations of **1zg** to **7zg**.

***N*-Phenylnicotinamide (7zg).** An oven-dried vessel containing a magnetic stir bar was charged with $Pd(TFA)_2$ (1.0 mg, 0.003 mmol, 1.0 mol %), Xantphos (2.6 mg, 0.0045 mmol, 1.5 mol %) CsF (68.4 mg, 0.45 mmol, 1.5 equiv) and anhydrous *N,N*-Dimethylformamide (2.0 mL, 0.15 M) in a nitrogen-filled glovebox. After stirring the reaction mixture at room temperature for 10 min, then **2** (0.36 mmol, 1.2 equiv) and aryl iodides **1zg** (0.3 mmol, 1.0 equiv) were added to the reaction mixture. The vessel was capped with a rubber septum and moved from the glovebox then stirred at 70 °C for 15 hour. Then, the mixtures were cooled down to room temperature, $NEt_3$ (418 μL, 3.0 mmol, 10.0 equiv) and $PhNH_2$ (81 μL, 0.9 mmol, 3.0 equiv) were added to the reaction mixture and the mixture was stirred at room temperature overnight. After quenching with $H_2O$ (20 mL), the mixture was extracted with AcOEt (3 × 20 mL) and the combined organic layer was dried over anhydrous $Na_2SO_4$, filtered and concentrated under reduced pressure. The crude residue was purified by silica gel flash chromatography (*n*-Hexane: AcOEt = 1:1) to afford the title compound **7zg** (36.8 mg, 62% yield) as a white solid. **$^1$H NMR** (300 MHz, $CDCl_3$) δ: 9.06 (s, 1H), 8.70 (d, *J* = 4.6 Hz, 1H), 8.51 (s, 1H), 8.18 (d, *J* = 8.1 Hz, 1H), 7.63 (d, *J* = 8.0 Hz, 2H), 7.40 – 7.33 (m, 3H), 7.17 (t, *J* = 7.4 Hz, 1H) ppm. **MS** (ESI): *m/z* 199 $[M+H]^+$. Spectroscopic data was agreement with the literature.[19]

***3zg** NMR yields (66%) were directly determined by $^{19}$F NMR analysis of the crude products using $C_6H_5F$

(28.5 μL, 0.3 mmol, 1.0 equiv) as an internal standard. **$^{19}$F NMR** (282 MHz, $CDCl_3$) δ: 20.31 (s, 1F) ppm.

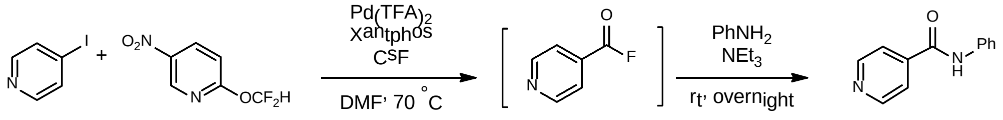


**Supplementary Figure 22.** One-pot amidations of **1zh** to **7zh**.

***N*-Phenylisonicotinamide (7zh).** An oven-dried vessel containing a magnetic stir bar was charged with $Pd(TFA)_2$ (1.0 mg, 0.003 mmol, 1.0 mol %), Xantphos (2.6 mg, 0.0045 mmol, 1.5 mol %) CsF (68.4 mg, 0.45 mmol, 1.5 equiv) and anhydrous *N,N*-Dimethylformamide (2.0 mL, 0.15 M) in a nitrogen-filled glovebox. After stirring the reaction mixture at room temperature for 10 min, then **2** (0.36 mmol, 1.2 equiv) and aryl iodides **1zh** (0.3 mmol, 1.0 equiv) were added to the reaction mixture. The vessel was capped with a rubber septum and moved from the glovebox then stirred at 70 °C for 15 hour. Then, the mixtures were cooled down to room temperature, $NEt_3$ (418 μL, 3.0 mmol, 10.0 equiv) and $PhNH_2$ (81 μL, 0.9 mmol, 3.0 equiv) were added to the reaction mixture and the mixture was stirred at room temperature overnight. After quenching with $H_2O$ (20 mL), the mixture was extracted with AcOEt (3 × 20 mL) and the combined organic layer was dried over anhydrous $Na_2SO_4$, filtered and concentrated under reduced pressure. The crude residue was purified by silica gel flash chromatography (*n*-Hexane: AcOEt = 1:1) to afford the title compound **7zh** (11.9 mg, 20% yield) as a white solid. **$^1$H NMR** (300 MHz, $CDCl_3$) δ: 10.49 (s, 1H), 8.79 (d, $J$ = 4.9 Hz, 2H), 7.86 (d, $J$ = 4.9 Hz, 2H), 7.77 (d, $J$ = 7.9 Hz, 2H), 7.38 (t, $J$ = 7.7 Hz, 2H), 7.14 (t, $J$ = 7.3 Hz, 1H) ppm. **MS** (ESI): *m/z* 199 $[M+H]^+$. Spectroscopic data was agreement with the literature.[19]

***3zh** NMR yields (27%) were directly determined by $^{19}$F NMR analysis of the crude products using $C_6H_5F$ (28.5 μL, 0.3 mmol, 1.0 equiv) as an internal standard. **$^{19}$F NMR** (282 MHz, $CDCl_3$) δ: 20.62 (s, 1F) ppm.

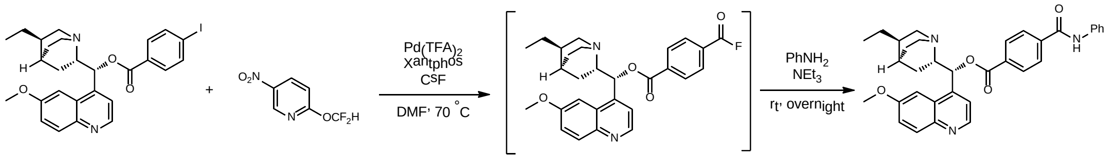


**Supplementary Figure 23.** One-pot amidations of **1zi** to **7zi**.

**(1*R*)-((2*S*,4*S*,5*R*)-5-Ethylquinuclidin-2-yl)(6-methoxyquinolin-4-yl)methyl 4-(phenylcarbamoyl)benzoate (7zi).** An oven-dried vessel containing a magnetic stir bar was charged with $Pd(TFA)_2$ (1.0 mg, 0.003 mmol, 1.0 mol %), Xantphos (2.6 mg, 0.0045 mmol, 1.5 mol %) CsF (68.4 mg, 0.45 mmol, 1.5 equiv) and anhydrous *N,N*-Dimethylformamide (2.0 mL, 0.15 M) in a nitrogen-filled glovebox. After stirring the reaction mixture at room temperature for 10 min, then **2** (0.36 mmol, 1.2 equiv)

and aryl iodides **1zi** (0.3 mmol, 1.0 equiv) were added to the reaction mixture. The vessel was capped with a rubber septum and moved from the glovebox then stirred at 70 °C for 15 hours. Then, the mixtures were cooled down to room temperature, $NEt_3$ (418 μL, 3.0 mmol, 10.0 equiv) and $PhNH_2$ (81 μL, 0.9 mmol, 3.0 equiv) were added to the reaction mixture and the mixture was stirred at room temperature overnight. After quenching with $H_2O$ (20 mL), the mixture was extracted with AcOEt (3 × 20 mL) and the combined organic layer was dried over anhydrous $Na_2SO_4$, filtered and concentrated under reduced pressure. The crude residue was purified by silica gel flash chromatography (DCM: MeOH = 19:1) to afford the title compound **7zi** (117.0 mg, 71% yield) as a white solid. **$^1$H NMR** (300 MHz, $CDCl_3$) δ: 8.67(d, *J* = 4.6 Hz, 1H), 8.63 (s, 1H), 8.11 (d, *J* = 7.9 Hz, 2H), 7.97 (dd, *J* = 17.1, 8.6 Hz, 3H), 7.64 (d, *J* = 8.0 Hz, 2H), 7.51 (s, 1H), 7.41 – 7.30 (m, 4H), 7.14 (t, *J* = 7.4 Hz, 1H), 6.73 (d, *J* = 6.5 Hz, 1H), 3.96 (s, 3H), 3.51 – 3.43 (m, 1H), 3.24 – 3.16 (m, 1H), 3.12 – 3.01 (m, 1H), 2.72 – 2.62 (m, 1H), 2.56 (s, 1H), 2.39 – 2.34 (m, 1H), 1.86 (s, 2H), 1.75 – 1.66 (m, 2H), 1.49 – 1.44 (m, 2H), 1.37 – 1.34 (m, 1H), 0.86 (t, *J* = 7.1 Hz, 3H) ppm. **$^{13}$C NMR** (126 MHz, $CDCl_3$) δ: 164.9, 164.7, 158.0, 147.2, 144.5, 143.6, 139.4, 137.7 132.2, 131.6 129.8, 129.0, 127.4, 126.8, 124.8, 121.9, 120.4, 118.5, 1013, 75.0, 59.2, 58.3, 55.6, 42.6, 37.3, 28.5, 27.7, 25.2, 23.9, 12.1 ppm. **HRMS** (ESI): Calcd. for $C_{34}H_{36}N_3O_4$ $[M+H]^+$ : 550.2706. Found: 550.2703. **IR** (NaCl): 3298, 3060, 2933, 2870, 1724, 1657, 1620, 1601, 1539, 1508, 1441, 1323, 1267, 1103, 1031, 1018, 754, 727, 692, 586 $cm^{-1}$. **m.p.**: 100.5 – 101.4 °C.

***3zi** NMR yields (71%) were directly determined by $^{19}$F NMR analysis of the crude products using $C_6H_5F$ (28.5 μL, 0.3 mmol, 1.0 equiv) as an internal standard. **$^{19}$F NMR** (282 MHz, $CDCl_3$) δ: 19.96 (s, 1F) ppm.

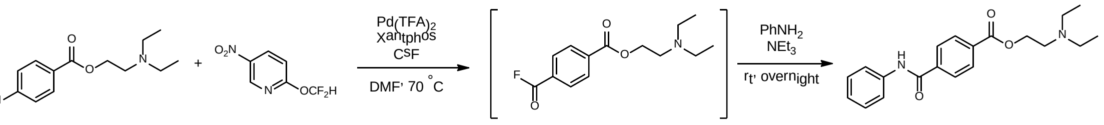


1zj 2 3zj 4zj

**Supplementary Figure 24.** One-pot amidations of **1zj** to **7zj**.

**2-(Diethylamino)ethyl 4-(phenylcarbamoyl)benzoate (7zj).** An oven-dried vessel containing a magnetic stir bar was charged with $Pd(TFA)_2$ (1.0 mg, 0.003 mmol, 1.0 mol %), Xantphos (2.6 mg, 0.0045 mmol, 1.5 mol %) CsF (68.4 mg, 0.45 mmol, 1.5 equiv) and anhydrous *N*,*N*-Dimethylformamide (2.0 mL, 0.15 M) in a nitrogen-filled glovebox. After stirring the reaction mixture at room temperature for 10 min, then **2** (0.36 mmol, 1.2 equiv) and aryl iodides **1zj** (0.3 mmol, 1.0 equiv) were added to the reaction mixture. The vessel was capped with a rubber septum and moved from the glovebox then stirred at 70 °C for 15 hours. Then, the mixtures were cooled down to room temperature, $NEt_3$ (418 μL, 3.0 mmol, 10.0 equiv) and $PhNH_2$ (81 μL, 0.9 mmol, 3.0 equiv) were added to the reaction mixture and the mixture was stirred at room temperature for overnight. After quenching with $H_2O$ (20 mL), the mixture was extracted with AcOEt (3 × 20 mL) and the

combined organic layer was dried over anhydrous $Na_2SO_4$, filtered and concentrated under reduced pressure. The crude residue was purified by silica gel flash chromatography (*n*-Hexane: AcOEt = 1:1) to afford the title compound **7zj** (43.9 mg, 43% yield) as a yellow semi-solid. **$^1$H NMR** (300 MHz, $CDCl_3$) δ: 8.14 (m, 2H), 7.93 – 7.91 (m, 3H), 7.65 (d, *J* = 7.9 Hz, 2H), 7.38 (t, *J* = 7.8 Hz, 2H), 7.18 (t, *J* = 7.5 Hz, 1H), 4.43 (t, *J* = 6.3 Hz, 2H), 2.87 (t, *J* = 6.2 Hz, 2H), 2.64 (q, *J* = 7.2 Hz, 4H), 1.08 (t, *J* = 7.2 Hz, 6H) ppm. **$^{13}$C NMR** (126 MHz, $CDCl_3$) δ: 165.7, 164.9, 138.8, 137.6, 133.1, 130.0, 129.1, 127.1, 124.9, 120.3, 63.8, 50.9, 47.8, 12.0 ppm. **HRMS** (ESI): Calcd. for $C_{20}H_{25}N_2O_3$ $[M+H]^+$ : 341.1865. Found: 341.1865. **IR** (NaCl): 3313, 2970, 2810, 1722, 1658, 1601, 1537, 1500, 1442, 1381, 1323, 1273, 1180, 1122, 1018, 870, 754, 727, 692, 511, 492 $cm^{-1}$.

***3zj** NMR yields (76%) were directly determined by $^{19}$F NMR analysis of the crude products using $C_6H_5F$ (28.5 μL, 0.3 mmol, 1.0 equiv) as an internal standard. **$^{19}$F NMR** (282 MHz, $CDCl_3$) δ: 19.83 (s, 1F) ppm.

**Preliminary Mechanistic Investigations (Supplementary Figure 24-27)**

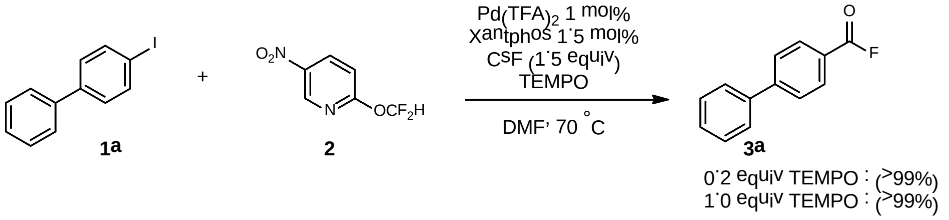


**Supplementary Figure 25.** TEMPO trapping experiment.

Following the general procedure **A**, adding TEMPO (0.06 mmol, 0.2 equiv) or (0.3 mmol, 1.0 equiv) in the glovebox and stirring the resulting mixture at 70 °C for 15 h. Yields were directly determined by $^{19}$F NMR analysis of the crude products using $C_6H_5F$ (28.5 μL, 0.3 mmol, 1.0 equiv) as an internal standard. The yield of **3a** was not decrease almost in these experiments, indicating the involvement of radical in this reaction is unlikely.

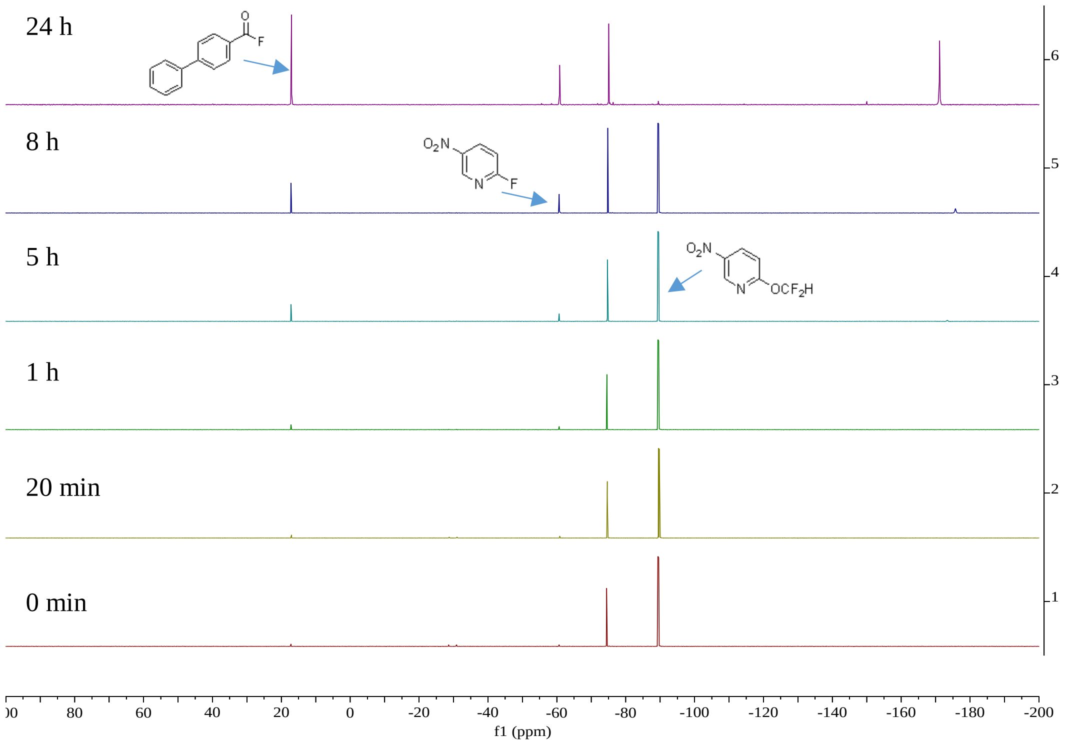


**Supplementary Figure 26.** $^{19}F$ NMR spectroscopic studies

An oven-dried NMR tube with a cap was charged with $Pd(TFA)_2$ (15.0 mg, 0.045 mmol, 5.0 mol %), Xantphos (39.1 mg, 0.0675 mmol, 7.5 mol %) CsF (1.03 g, 6.75 mmol, 1.5 equiv) and anhydrous *N*,*N*-Dimethylformamide-*d7* (0.4 mL) in a nitrogen-filled glovebox. The mixture with NMR tube was stirred for 10 min in test tube with a magnetic stir bar, then **2** (1.03 g, 5.4 mmol, 1.2 equiv) and aryl iodides **1a** (1.26 g, 0.1 mmol, 1.0 equiv) was dissolved with *N*,*N*-Dimethylformamide-*d7* (0.3 mL) and added. The NMR tube with the mixture was sealed and removed from the glovebox, then stirred at 70 °C. The reaction progress was monitored by $^{19}F$ NMR as shown in the chart.

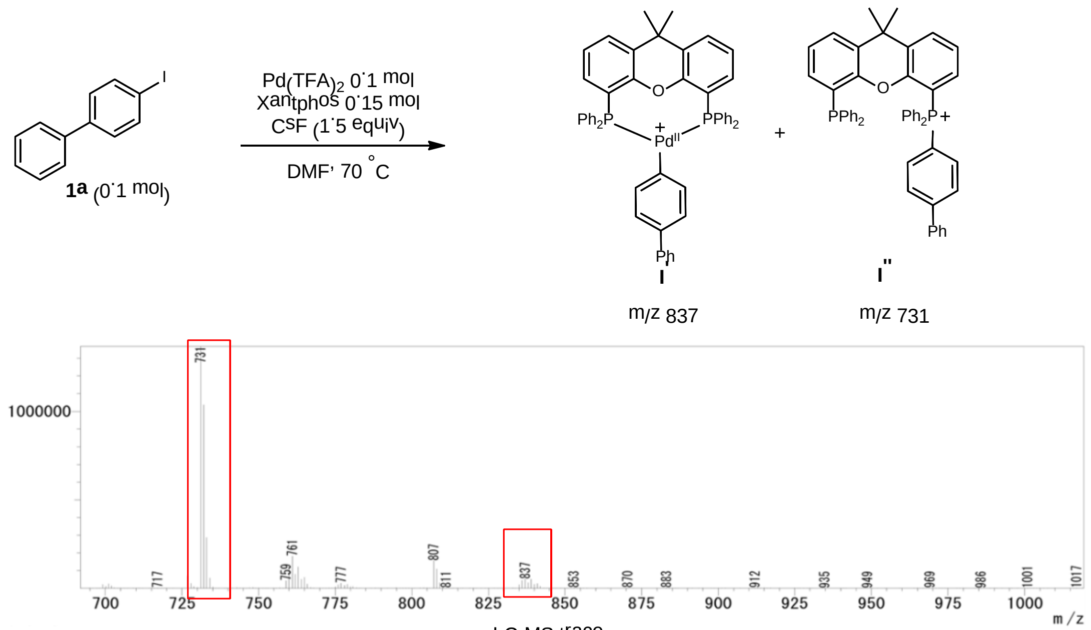

LC MS trace

**Supplementary Figure 27.** LCMS Experiments

An oven-dried tube with a rubber septum and magnetic stir bar was charged with Pd(TFA)$_2$ (33.2 mg, 0.1 mmol, 1.0 equiv), Xantphos (86.8 mg, 0.15 mmol, 1.5 equiv) anhydrous *N*,*N*-Dimethylformamide (0.7 mL) in a nitrogen-filled glovebox. The mixture was stirred for 10 min then aryl iodides **1a** (20.0 mg, 0.1 mmol, 1.0 equiv) was added. The vessel was capped with a rubber septum and moved from the glovebox then stirred at 70 °C for 2 hour. The reaction mixture was cooled to rt, the crude solution was sent to LC-MS analysis.

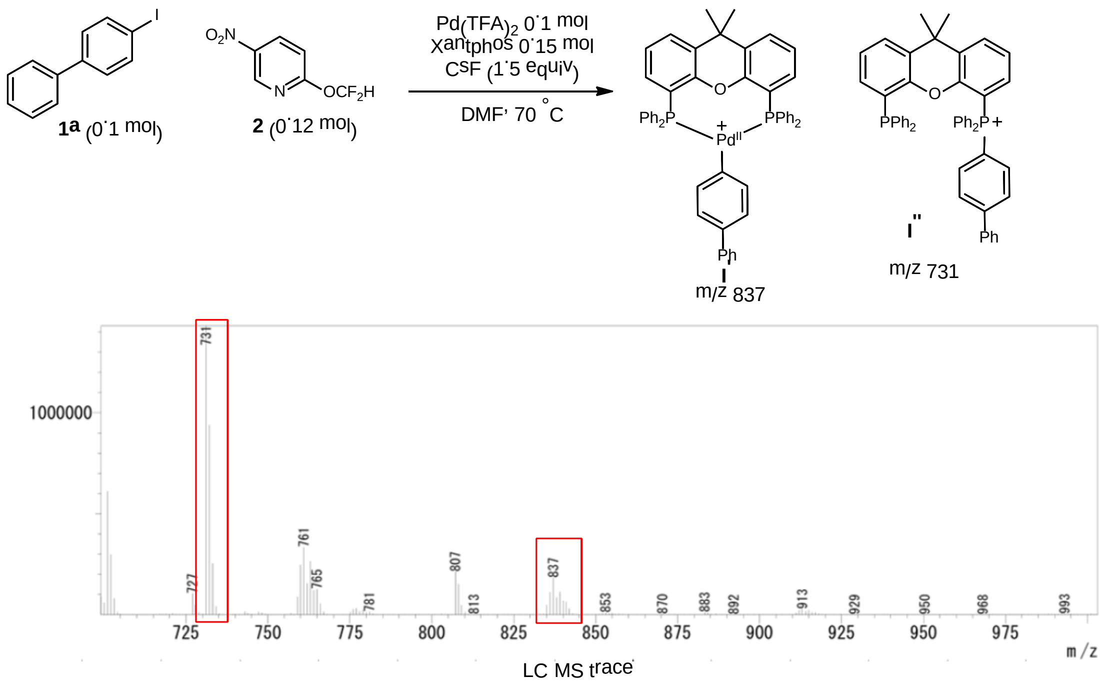


**Supplementary Figure 28.** LCMS Experiments

An oven-dried tube with a rubber septum and magnetic stir bar was charged with Pd(TFA)$_2$ (33.2 mg, 0.1 mmol, 1.0 equiv), Xantphos (86.8 mg, 0.15 mmol, 1.5 equiv) CsF (22.8 mg, 0.15 mmol, 1.5 equiv) and anhydrous *N,N*-Dimethylformamide (1.0 mL) in a nitrogen-filled glovebox. The mixture was stirred for 10 min then **2** (22.7 mg, 0.12 mmol, 1.2 equiv) and aryl iodides **1a** (20.0 mg, 0.1 mmol, 1.0 equiv) was dissolved were added. The vessel was capped with a rubber septum and moved from the glovebox then stirred at 70 °C for 2 hour. The reaction mixture was cooled to rt, then the crude solution was sent to LC-MS analysis.

## Supplementary References

1. Pawluc, P., Hreczycho, G., Szudkowska, J., Kubicki, M. & Marciniec, B. New one-pot synthesis of (E)-β-aryl vinyl halides from styrenes. Org. Lett. **11**, 3390-3393 (2009).
2. Zheng, J., Lin, J. H., Deng, X. Y. & Xiao, J. C. 1, 8-diazabicyclo [5.4. 0] undec-7-ene (DBU)-promoted decomposition of difluorocarbene and the subsequent trifluoromethylation. Org. Lett. **17**, 532-535 (2015).
3. Radeke, H., Hanson, K., Yalamanchili, P., Hayes, M., Zhang, Z.Q., Azure, M., Yu, M., Guaraldi, M., Kagan, M., Robinson, S. & Casebier, D. Synthesis and biological evaluation of the mitochondrial complex 1 inhibitor 2-[4-(4-fluorobutyl) benzylsulfanyl]-3-methylchromene-4-one as a potential cardiac positron emission tomography tracer. J. Med. Chem. **50**, 4304-4315 (2007).
4. Kamiya, N., Chikami, Y. & Ishii, Y. Stereoselective synthesis of internal alkenyl iodides from alkynes via addition of hydrogen iodide generated in situ from a chlorotrimethylsilane/sodium iodide/water system. Synlett **1990**, 675-676 (1990).

5. Breyer, S., Semmler, A., Miller, T., Hill, A., Geissler, S., Haberkorn, U. & Mier, W. Radioiodinated dechloro-4-iodofenofibrate: A hydrophobic model drug for molecular imaging studies. Int. J. Pharm. **431**, 78-83 (2012).
6. Boddy, A.J., Affron, D.P., Cordier, C.J., Rivers, E.L., Spivey, A.C. & Bull, J.A. Rapid assembly of saturated nitrogen heterocycles in one-pot: diazo-heterocycle "stitching" by N–H insertion and cyclization. Angew. Chem., Int. Ed. **58**, 1458-1462 (2019).
7. Sheng, J., Ni, H.Q., Zhang, H.R., Zhang, K.F., Wang, Y.N. & Wang, X. S. Nickel‐catalyzed reductive cross‐coupling of aryl halides with monofluoroalkyl halides for late‐stage monofluoroalkylation. Angew. Chem., Int. Ed. **57**, 7634-7639 (2018).
8. Hartz, R.A., Ahuja, V.T., Zhuo, X., Mattson, R.J., Denhart, D.J., Deskus, J.A., Vrudhula, V.M., Pan, S., Ditta, J.L., Shu, Y.Z. & Grace, J.E. A strategy to minimize reactive metabolite formation: discovery of (*S*)-4-(1-cyclopropyl-2-methoxyethyl)-6-[6-(difluoromethoxy)-2, 5-dimethylpyridin-3-ylamino]-5-oxo-4, 5-dihydropyrazine-2-carbonitrile as a potent, orally bioavailable corticotropin-releasing factor-1 receptor antagonist. J. Med. Chem. ***52***, 7653-7668 (2009).
9. Keaveney, S.T. & Schoenebeck, F. Palladium-catalyzed decarbonylative trifluoromethylation of acid fluorides. Angew. Chem., Int. Ed. **57**, 4073-4077 (2018).
10. Beaulieu, F., Beauregard, L.P., Courchesne, G., Couturier, M., LaFlamme, F. & L'Heureux, A. Aminodifluorosulfinium tetrafluoroborate salts as stable and crystalline deoxofluorinating reagents. Org. Lett. **11**, 5050-5053 (2009).
11. Birrell, J.A., Desrosiers, J.N. & Jacobsen, E.N. Enantioselective acylation of silyl ketene acetals through fluoride anion-binding catalysis. J. Am. Chem. Soc. **133**, 13872-13875 (2011).
12. Munoz, S.B., Dang, H., Ispizua-Rodriguez, X., Mathew, T. & Prakash, G.S. Direct access to acyl fluorides from carboxylic acids using a phosphine/fluoride deoxyfluorination reagent system. Org. Lett. **21**, 1659-1663 (2019).
13. Wang, Z., Wang, X. & Nishihara, Y. Nickel-catalysed decarbonylative borylation of aroyl fluorides. Chem. Commun. **54**, 13969-13972 (2018).
14. Ueda, T., Konishi, H. & Manabe, K. Palladium-catalyzed fluorocarbonylation using N-formylsaccharin as CO source: general access to carboxylic acid derivatives. Org. Lett. **15**, 5370-5373 (2013).
15. Ryan, S.J., Candish, L. & Lupton, D.W. N-heterocyclic carbene-catalyzed (4+ 2) cycloaddition/decarboxylation of silyl dienol ethers with α, β-unsaturated acid fluorides. J. Am. Chem. Soc. **133**, 4694-4697 (2011).
16. Schaumburg, K. The $^{19}F$ and $^{1}H$ nuclear magnetic resonance spectra of 2-thienylcarbonyl fluoride and 2-thiazolylcarbonyl fluoride. Can. J. Chem. **49**, 1146-1148 (1971).
17. Okuda, Y., Xu, J., Ishida, T., Wang, C.A. & Nishihara, Y. Nickel-catalyzed decarbonylative alkylation of aroyl fluorides assisted by Lewis-acidic organoboranes. ACS Omega **3**, 13129-13140 (2018).
18. Rewcastle, G.W., Denny, W.A., Winters, R.T., Colbry, N.L. & Showalter, H.H. Synthesis of 6-substituted pyrido [3,4-*d*] pyrimidin-4 (3 H)-ones via directed lithiation of 2-substituted 5-aminopyridine derivatives. J. Chem. Soc., Perkin Trans. 1, 2221-2226 (1996).

19. Wang, S.M., Zhao, C., Zhang, X. & Qin, H.L. Clickable coupling of carboxylic acids and amines at room temperature mediated by $SO_2F_2$: a significant breakthrough for the construction of amides and peptide linkages. Org. Biomol. Chem. **17**, 4087-4101 (2019).
20. Kubota, Y., Hanaoka, T.A., Takeuchi, K. & Sugi, Y. An efficient synthesis of aryl esters by palladium-catalyzed carbonylation of 4-bromobiphenyl. Synlett **1994**, 515-517 (1994).
21. Wang, Q., Liu, L., Dong, J., Tian, Z. & Chen, T. Metal-free thioesterification of amides generating acyl thioesters. New J. Chem. **43**, 9384-9388 (2019).
22. Ogiwara, Y., Sakino, D., Sakurai, Y. & Sakai, N. Acid fluorides as acyl electrophiles in Suzuki–Miyaura coupling. Eur. J. Org. Chem. **2017**, 4324-4327 (2017).
23. Uchiyama, M., Furuyama, T., Kobayashi, M., Matsumoto, Y. & Tanaka, K. Toward a protecting-group-free halogen−metal exchange reaction: practical, chemoselective metalation of functionalized aromatic halides using dianion-type zincate, $^{t}Bu_4ZnLi_2$. J. Am. Chem. Soc. **128**, 8404-8405 (2006).
24. Liu, L., Dong, Y., Pang, B. & Ma, J. [Bmim]$PF_6$-promoted ligandless Suzuki–Miyaura coupling reaction of potassium aryltrifluoroborates in water. J. Org. Chem. **79**, 7193-7198 (2014).
25. Ogiwara, Y., Sakurai, Y., Hattori, H. & Sakai, N. Palladium-catalyzed reductive conversion of acyl fluorides via ligand-controlled decarbonylation. Org, Lett. **20**, 4204-4208 (2018).
26. Guo, Z., Liu, Q., Wei, X., Zhang, Y., Tong, H., Chao, J., Guo, J. & Liu, D. 2-Aminopyrrolyl dilithium compounds: Synthesis, structural diversity, and catalytic activity for amidation of aldehydes with amines. Organometallics **32**, 4677-4683 (2013).

## $^{1}$H NMR Spectra, $^{19}$F NMR Spectra and $^{13}$C NMR Spectra (Supplementary Figure 28-130)

**(1*S*,2*R*,5*S*) -2-Isopropyl-5-methylcyclohexyl 4-iodobenzoate (1w)**

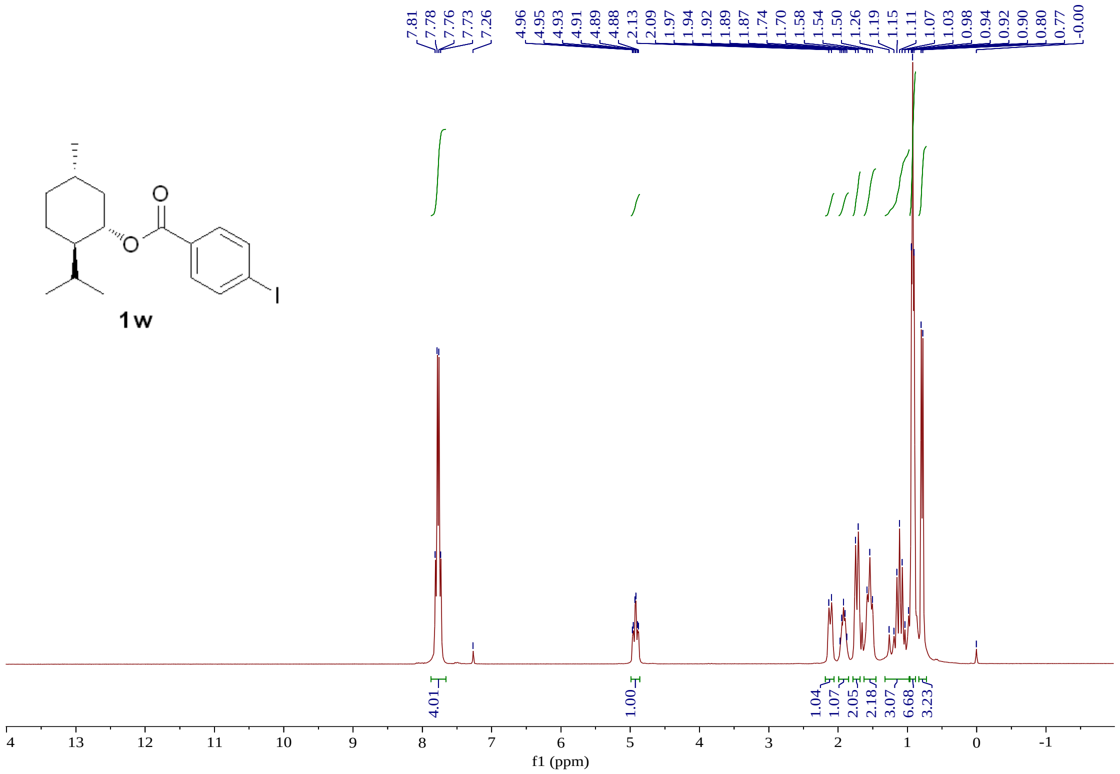


**Supplementary Figure 29.** $^{1}$H NMR Spectra of **1w.**

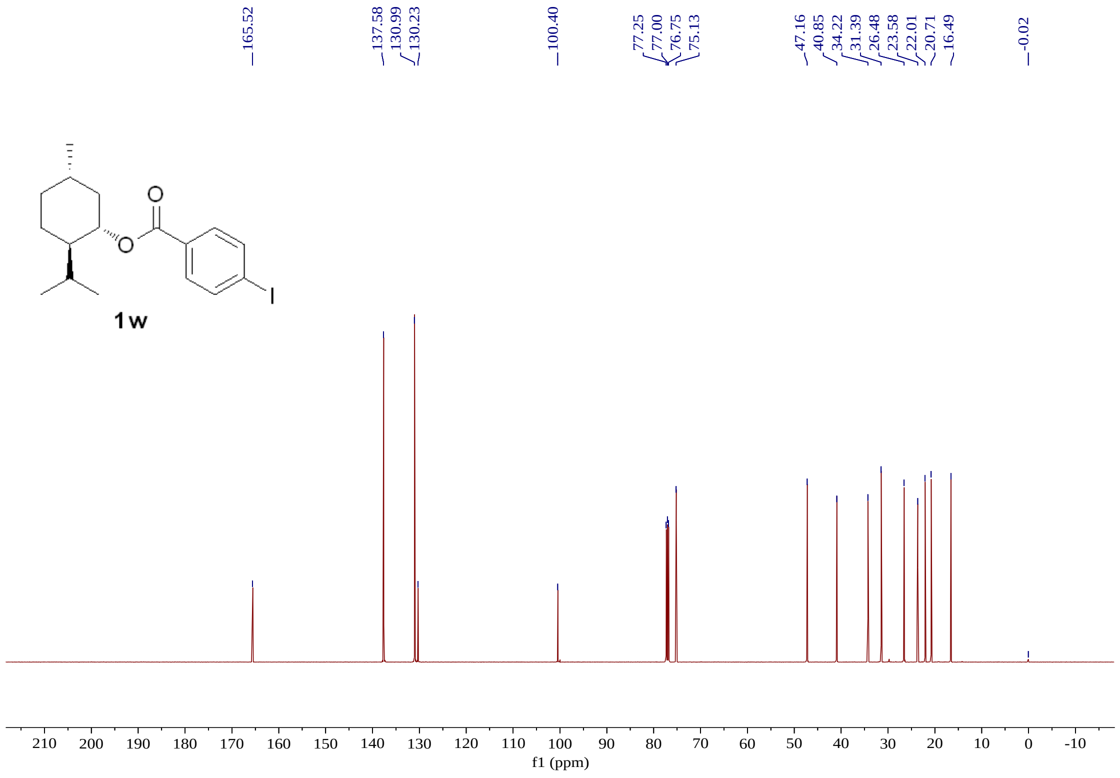


**Supplementary Figure 30.**$^{13}$C NMR Spectra of **1w.**

**(8*R*,9*S*,13*S*,14*S*)-13-methyl-17-oxo-7,8,9,11,12,13,14,15,16,17-decahydro-6*H*-cyclopenta[*a*]phenanthren-3-yl 4-iodobenzoate (1z)**

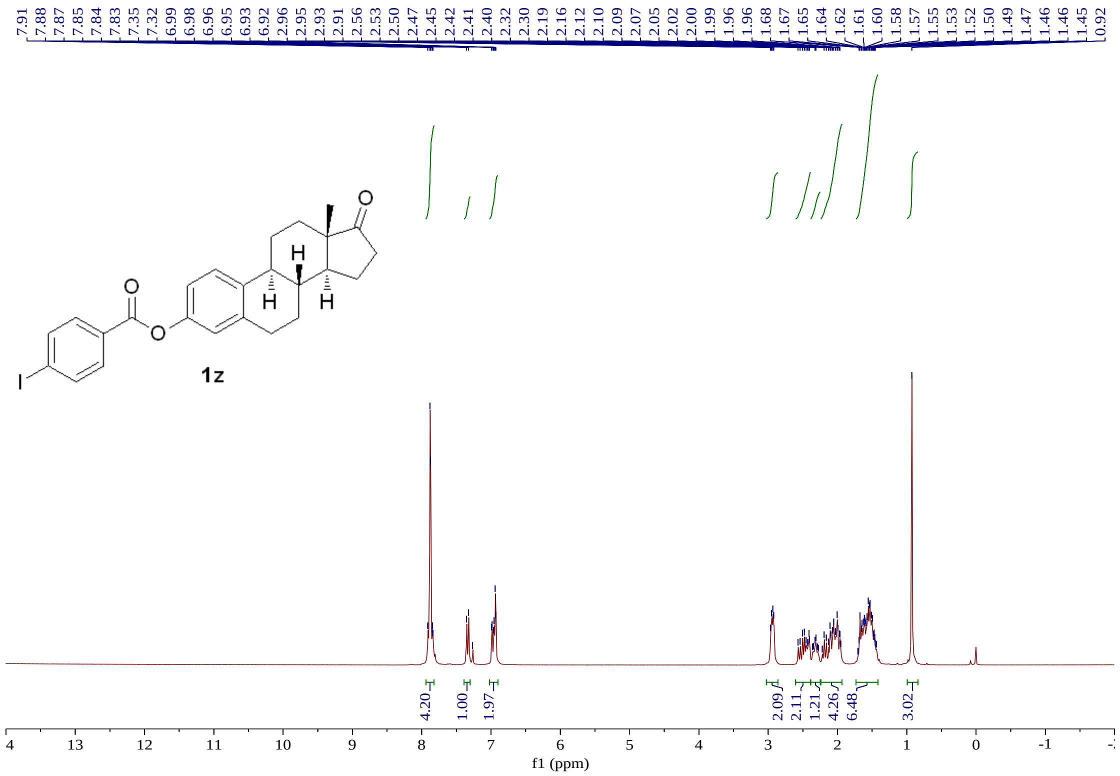

**Supplementary Figure 31.** $^{1}$H NMR Spectra of **1z.**

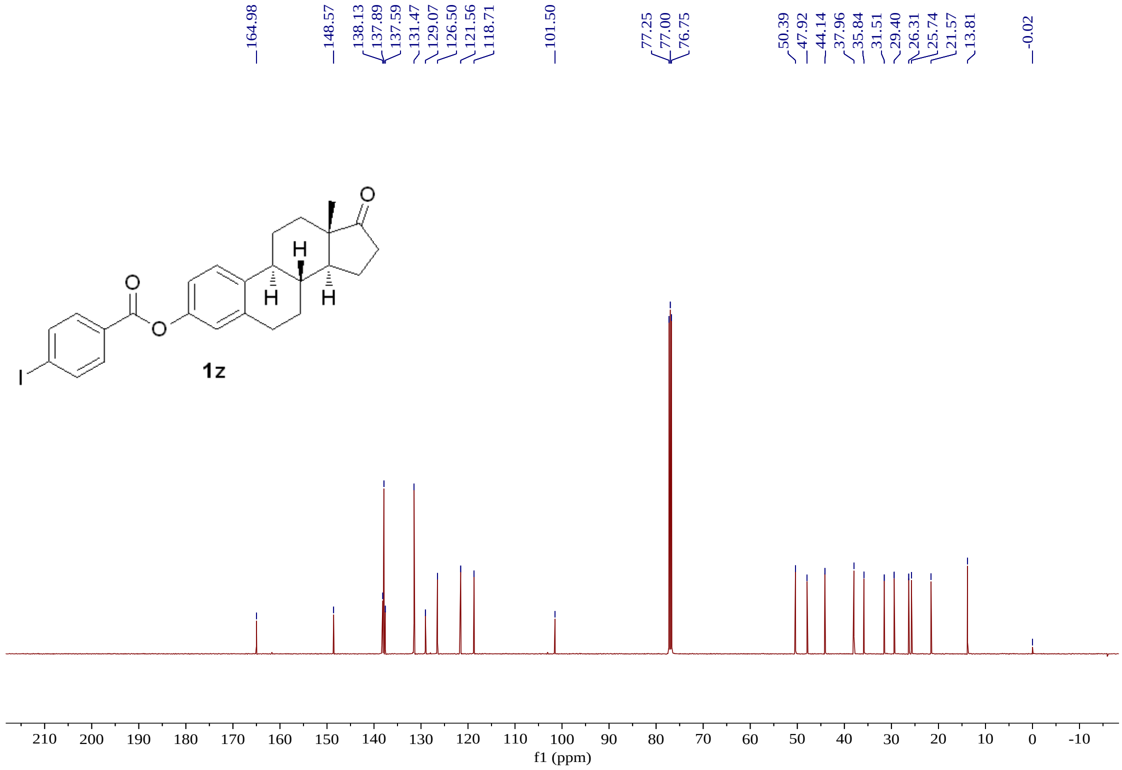

**Supplementary Figure 32.** $^{13}$C NMR Spectra of **1z.**

**4-Iodobenzyl 2-(11-oxo-6,11-dihydrodibenzo[*b*,*e*]oxepin-2-yl)acetate (1za)**

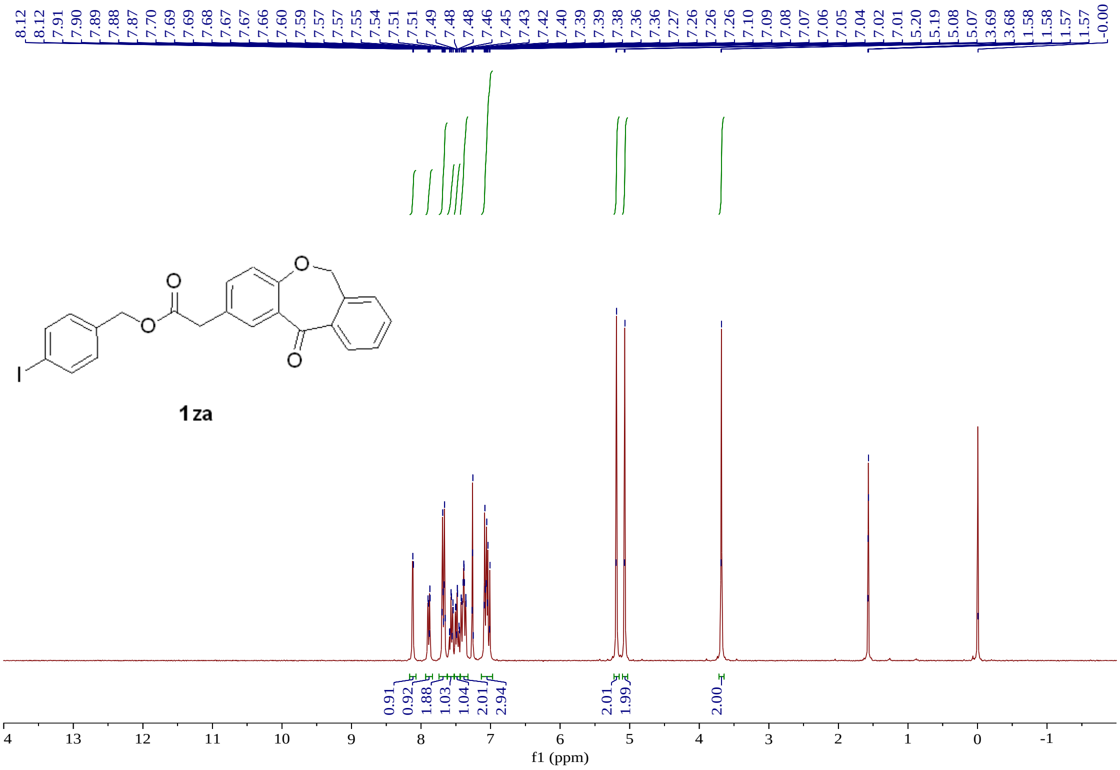


**Supplementary Figure 33.** $^{1}$H NMR Spectra of **1za.**

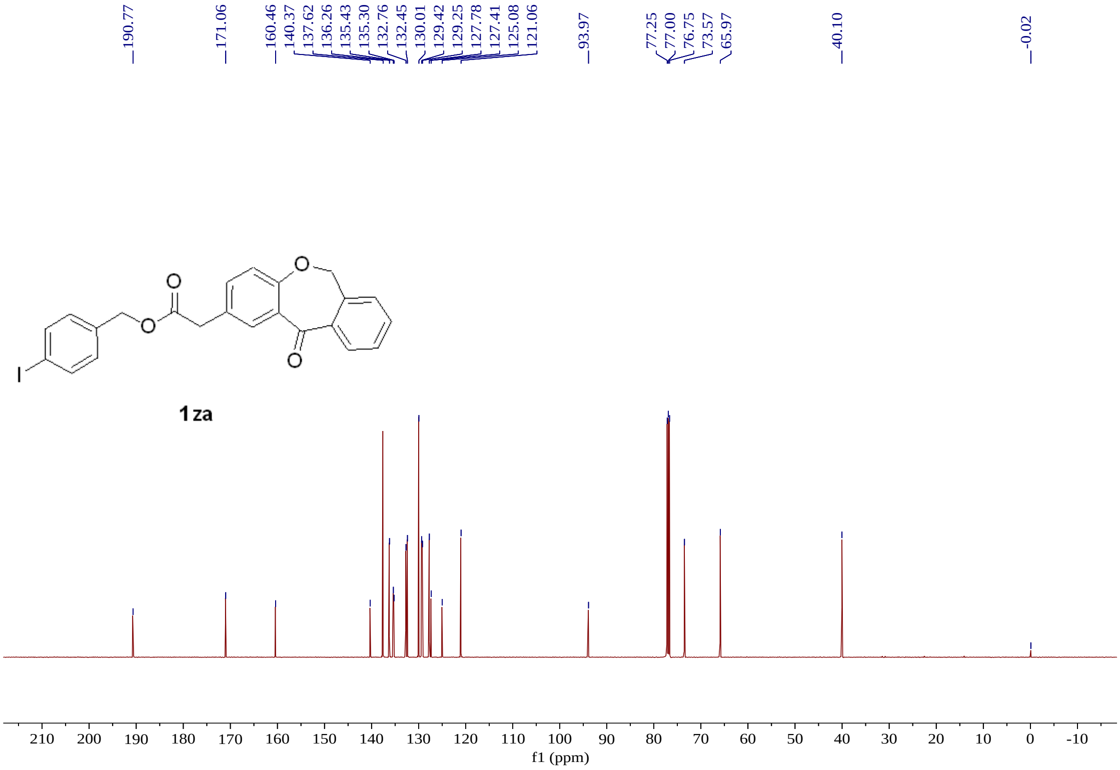


**Supplementary Figure 34.** $^{13}$C NMR Spectra of **1za.**

**(All-rac)-6-((4-Iodobenzyl)oxy)-2,5,7,8-tetramethyl-2-(4,8,12-trimethyltridecyl)chroman (1zb)**

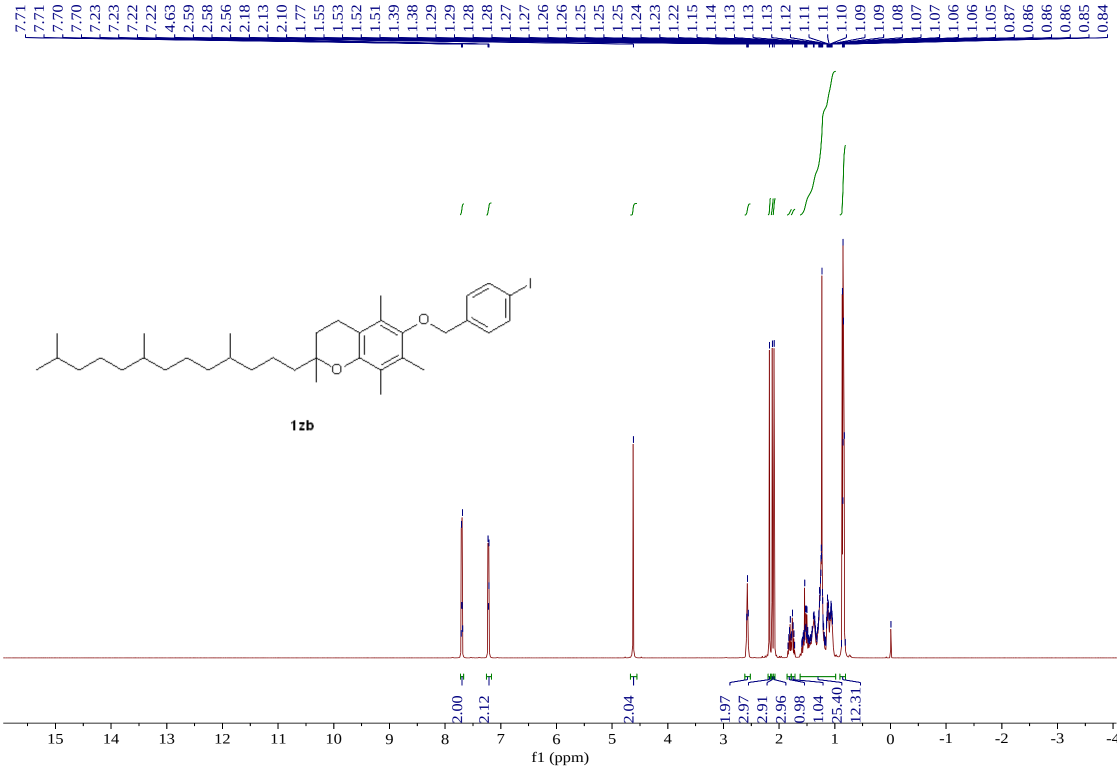


**Supplementary Figure 35.** $^{1}$H NMR Spectra of **1zb.**

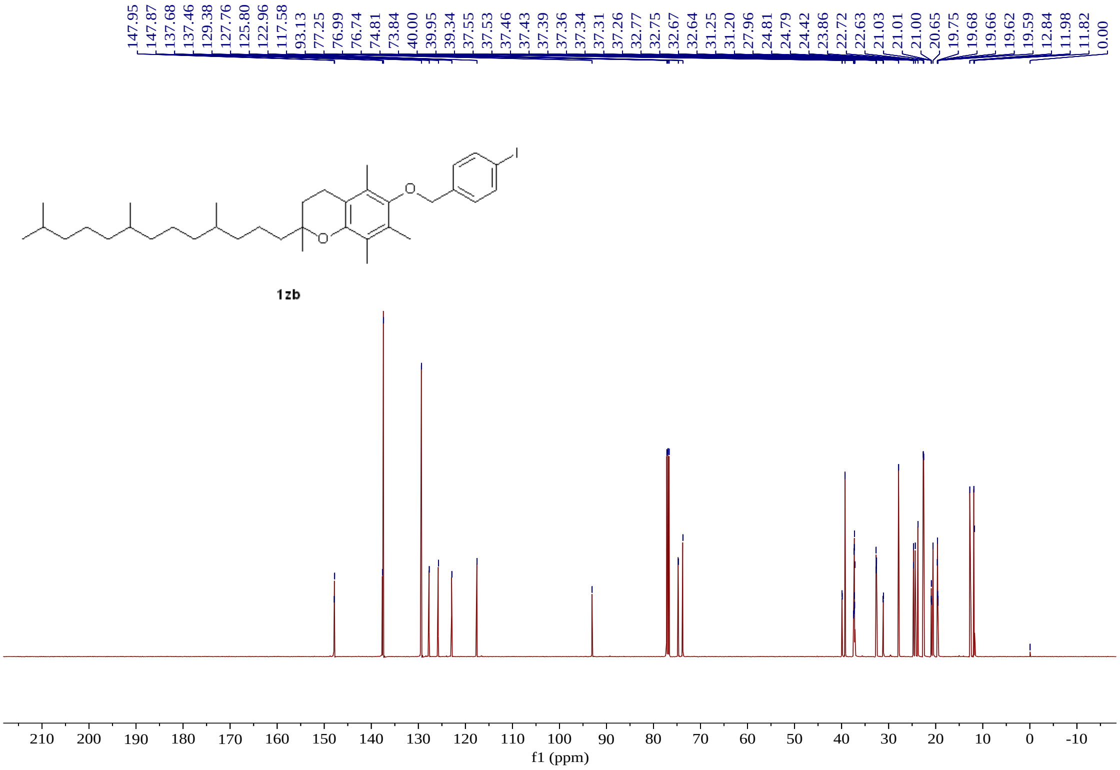


**Supplementary Figure 36.** $^{13}$C NMR Spectra of **1zb.**

**(8*R*,9*S*,10*R*,13*S*,14*S*,17*R*)-10,13-dimethyl-3-oxo-2,3,6,7,8,9,10,11,12,13,14,15,16,17-tetradecahydro-1*H*-**

**cyclopenta[*a*]phenanthren-17-yl 4-iodobenzoate (1zc)**

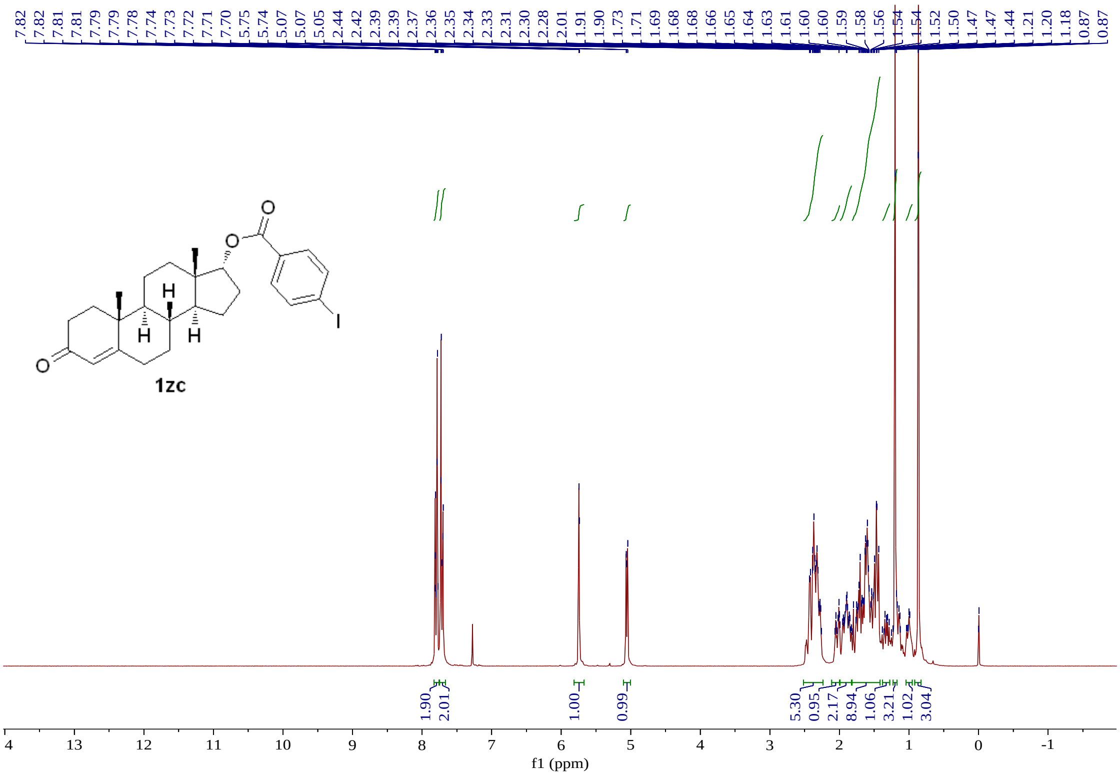

**Supplementary Figure 37** $^{1}H$ NMR Spectra of **1zc.**

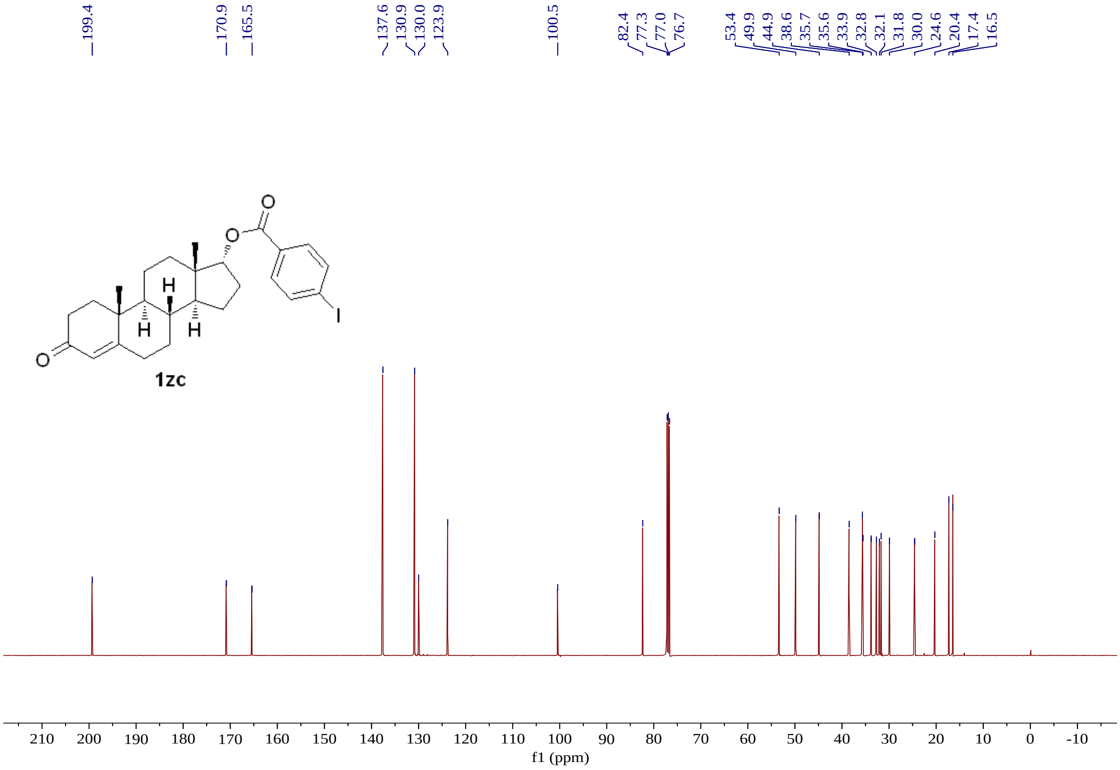

**Supplementary Figure 38.** $^{13}C$ NMR Spectra of **1zc.**

**(1*R*)-((2*S*,4*S*,5*R*)-5-ethylquinuclidin-2-yl)(6-methoxyquinolin-4-yl)methyl 4-iodobenzoate (1zi)**

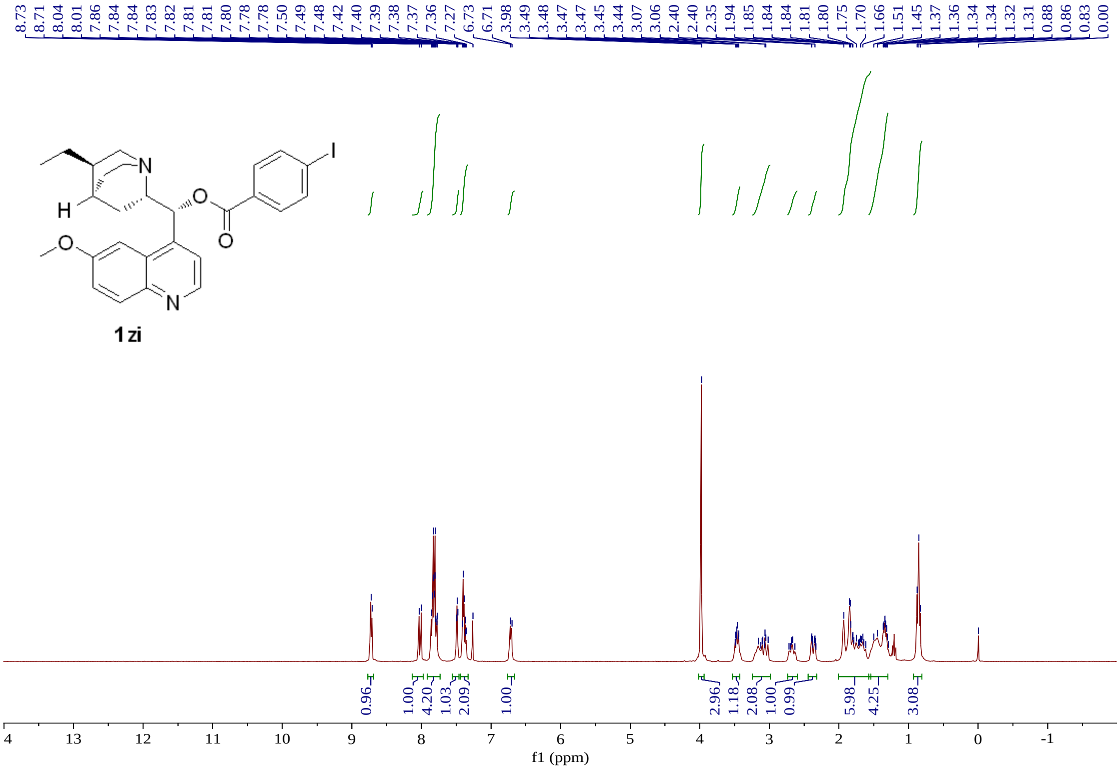


**Supplementary Figure 39.** $^{1}$H NMR Spectra of **1zi.**

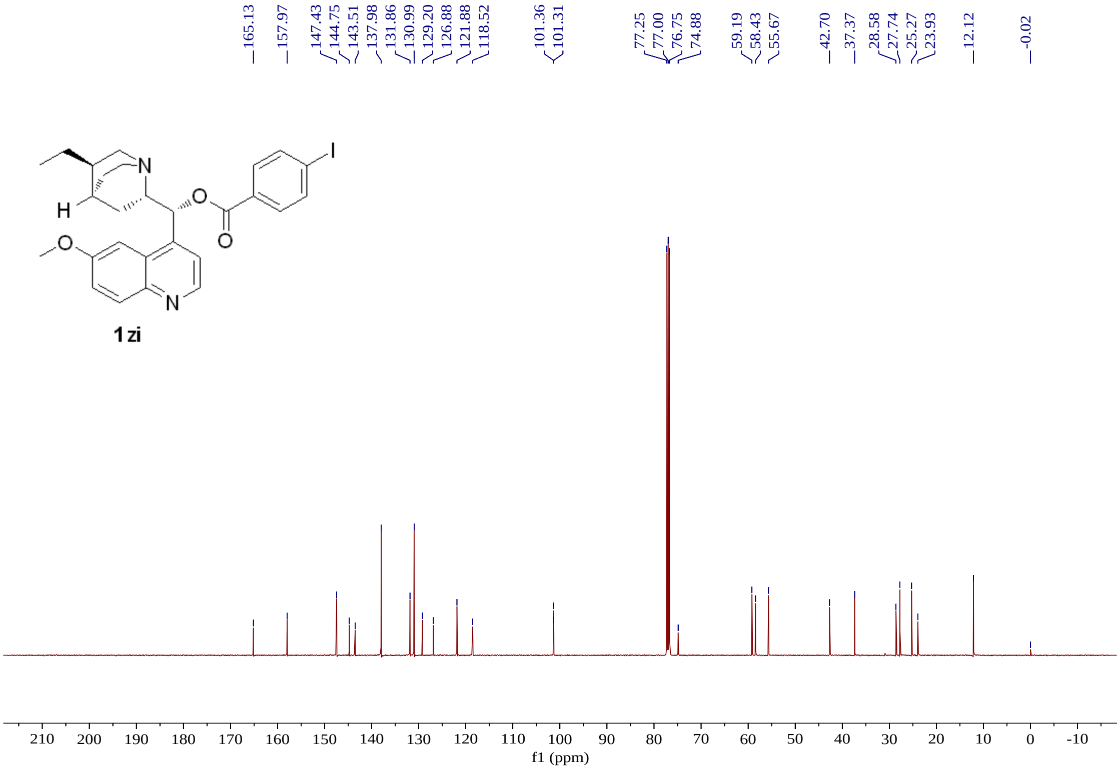


**Supplementary Figure 40.** $^{13}$C NMR Spectra of **1zi.**

**2-(Difluoromethoxy)-5-nitropyridine (2)**

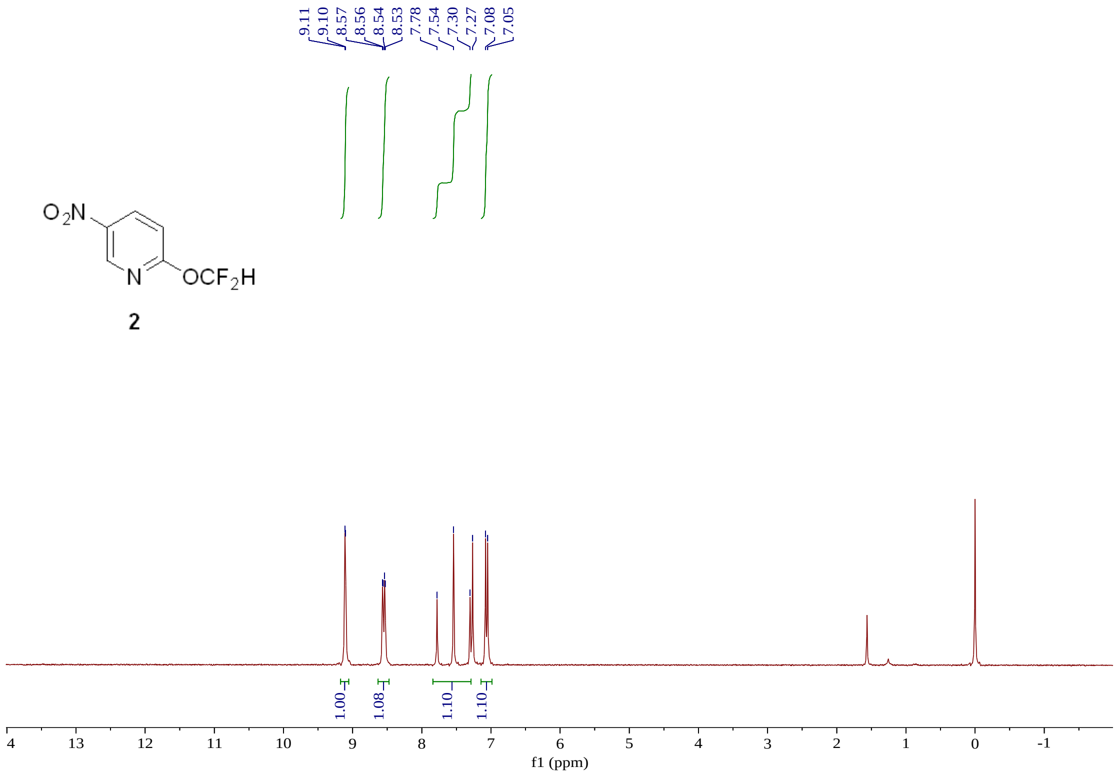


**Supplementary Figure 41.** $^{1}$H NMR Spectra of **2.**

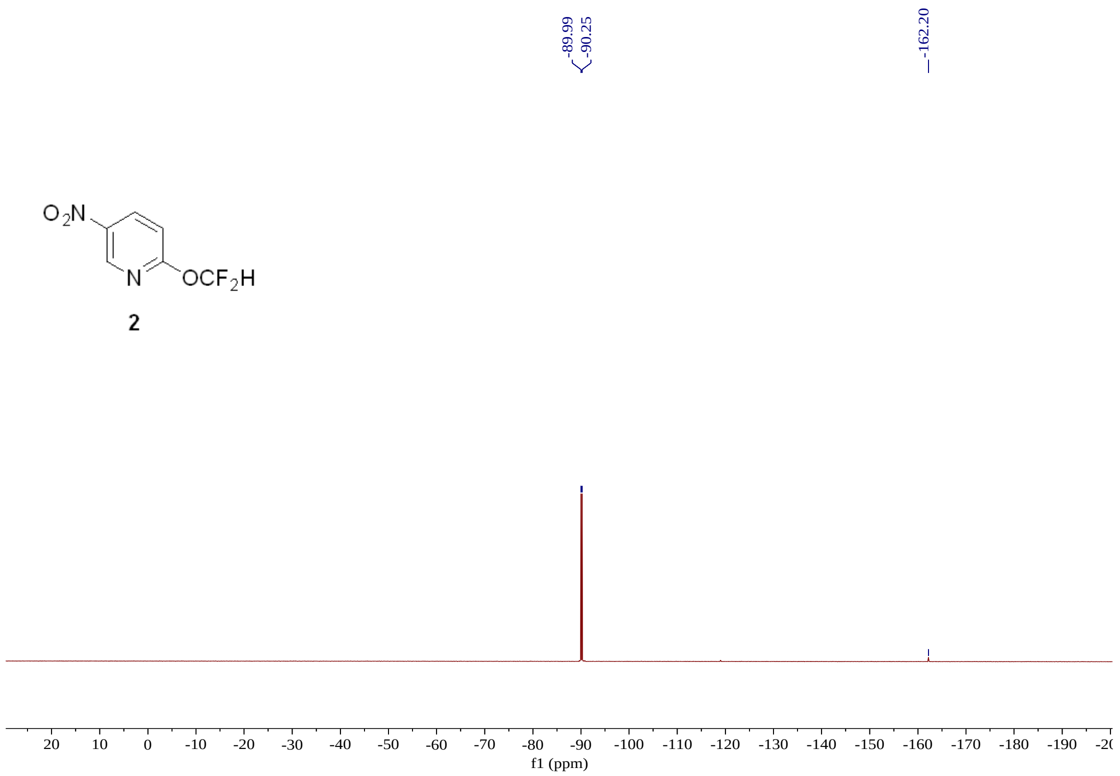


**Supplementary Figure 42.** $^{19}$F NMR Spectra of **2.**

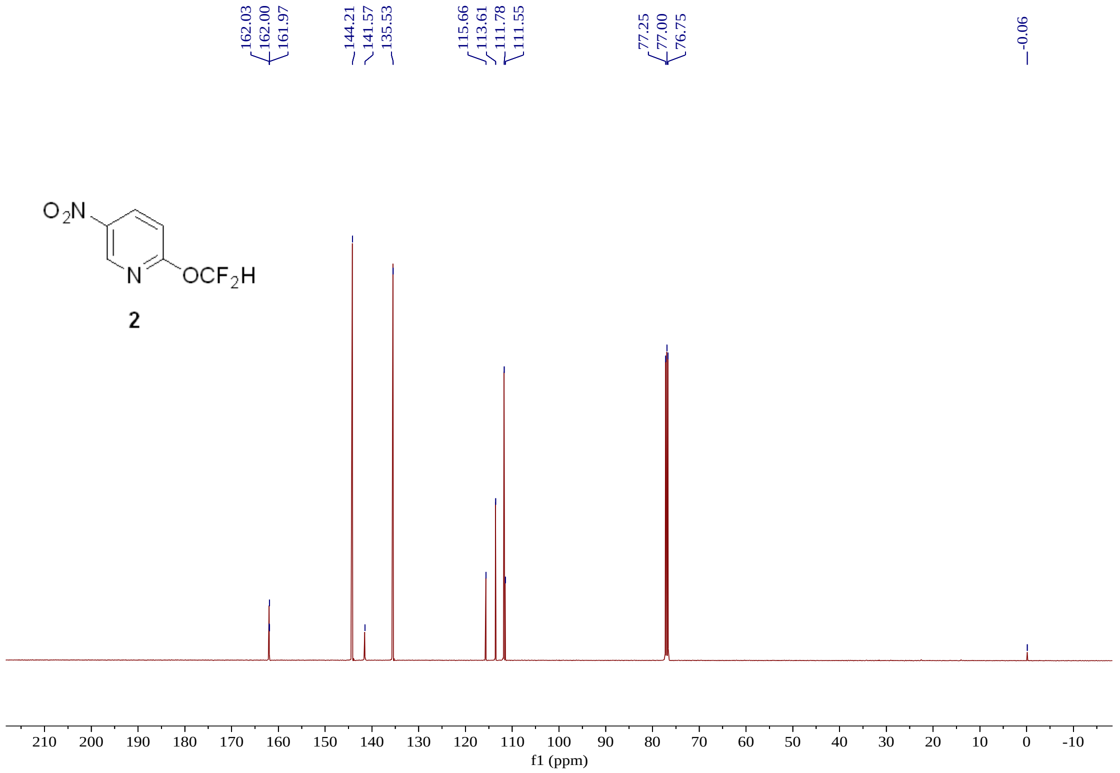


**Supplementary Figure 43.** $^{13}$C NMR Spectra of **2.**

**[1,1'-Biphenyl]-4-carbonyl fluoride (3a)**

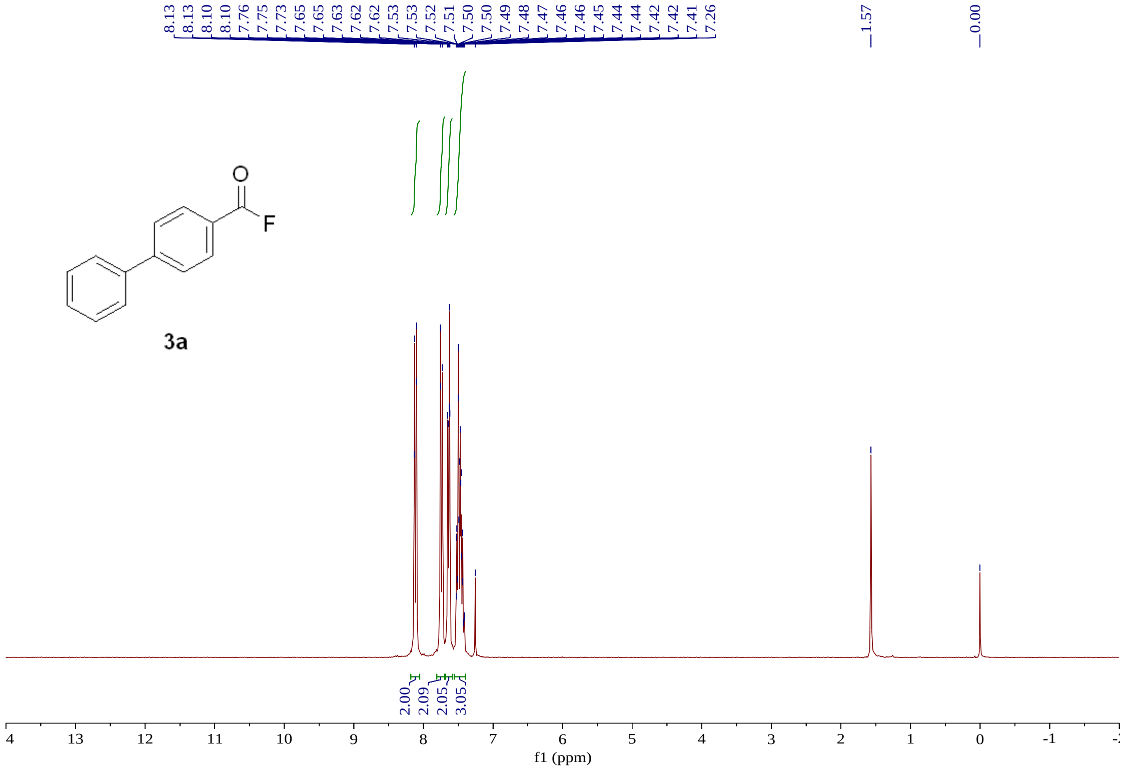


**Supplementary Figure 44.** $^{1}$H NMR Spectra of **3a.**

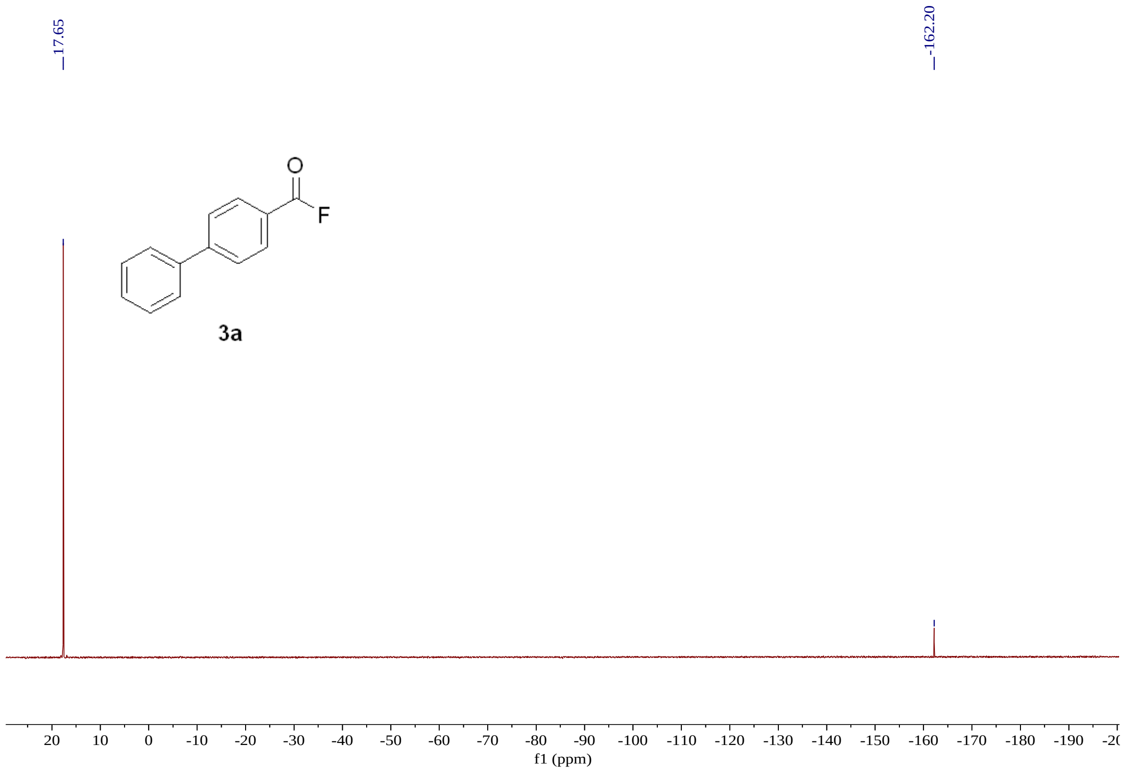

**Supplementary Figure 45.** $^{19}$F NMR Spectra of **3a.**

**Benzoyl fluoride (3b)**

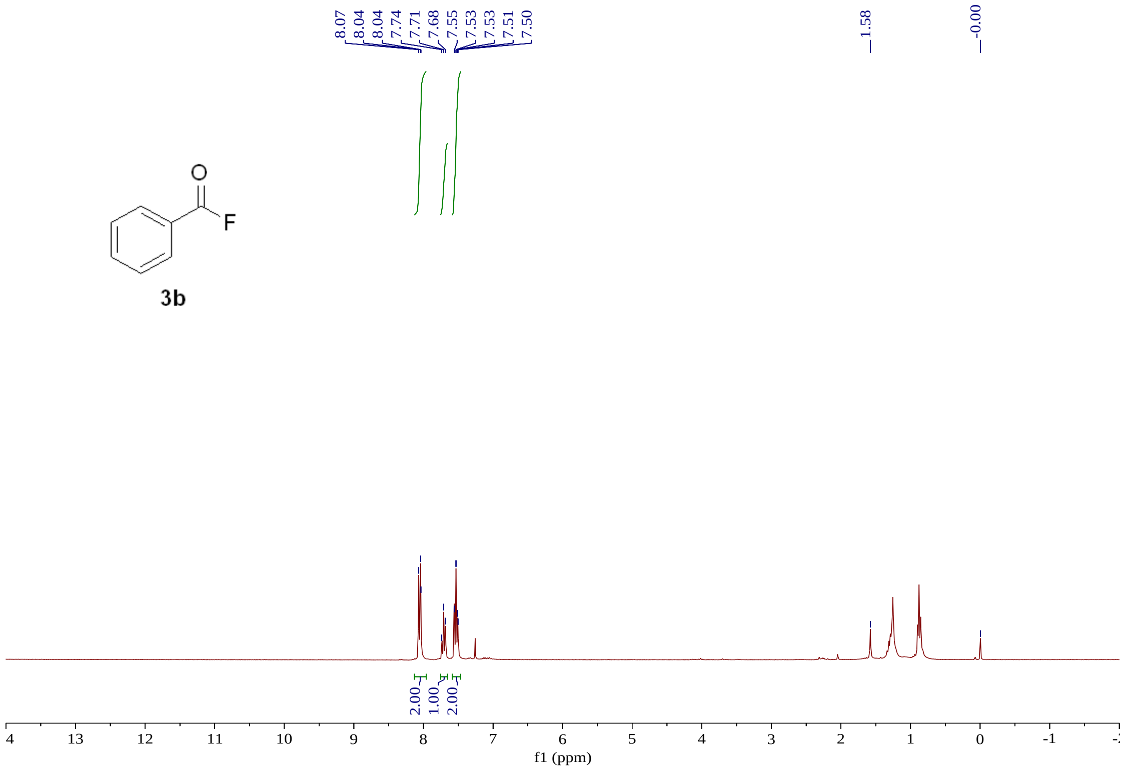

**Supplementary Figure 46.** $^{1}$H NMR Spectra of **3a.**

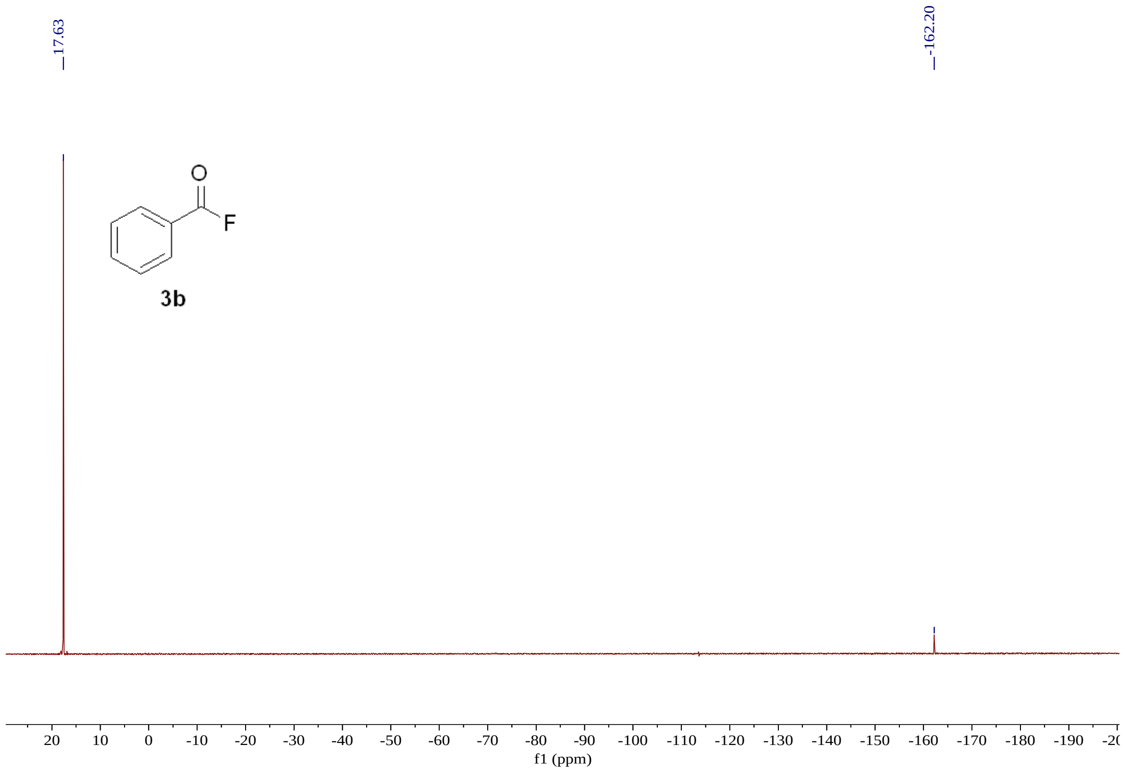


**Supplementary Figure 47.** $^{19}$F NMR Spectra of **3.**

## 4-Methylbenzoyl fluoride (3c)

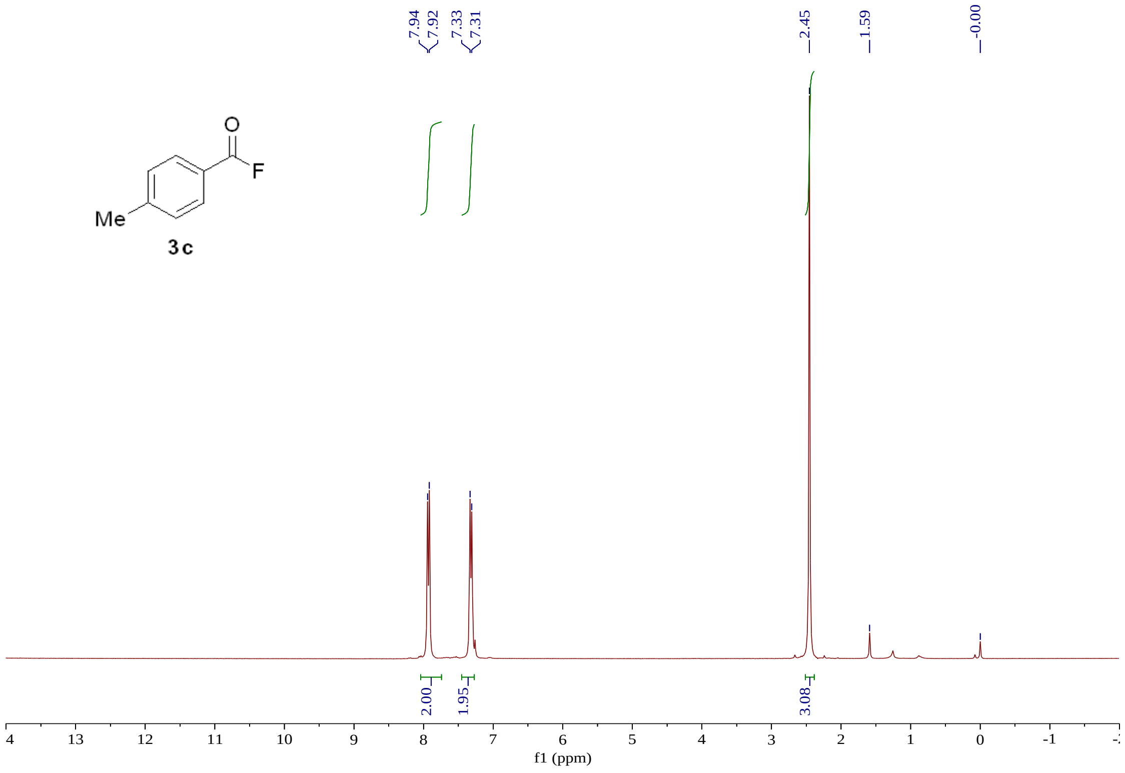


**Supplementary Figure 48.** $^{1}$H NMR Spectra of **3c.**

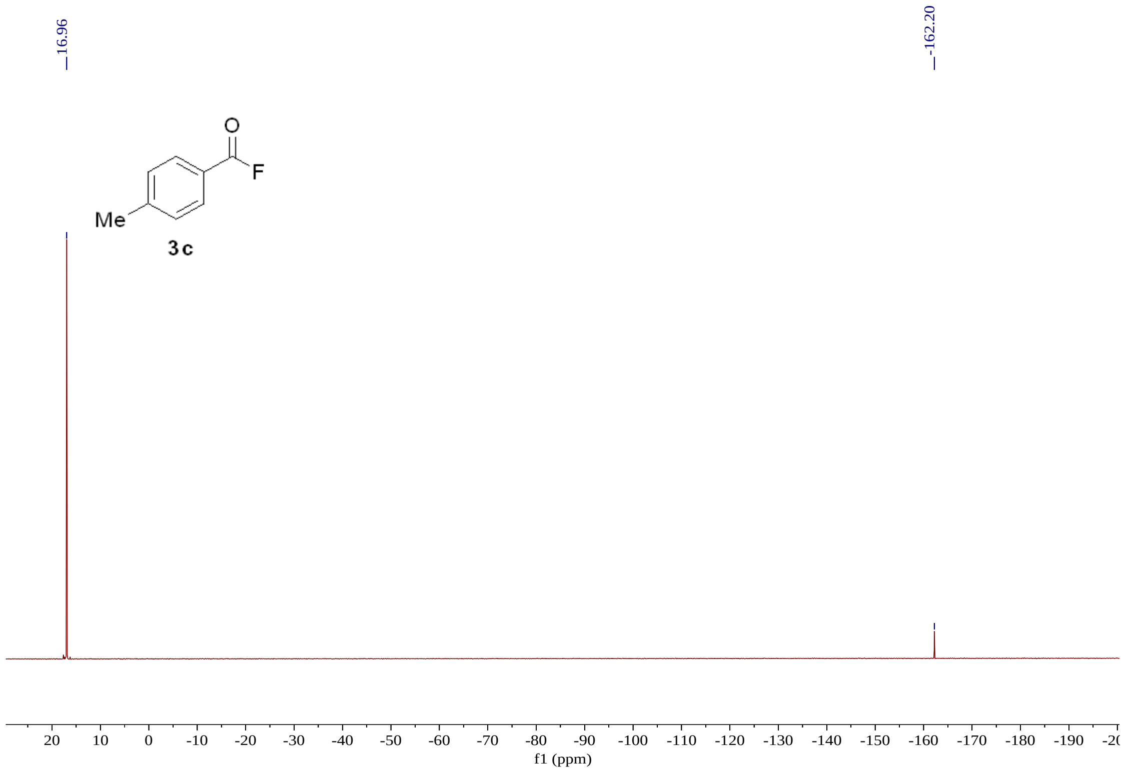


**Supplementary Figure 49.** $^{19}$F NMR Spectra of **3c.**

**4-Methoxybenzoyl fluoride (3d)**

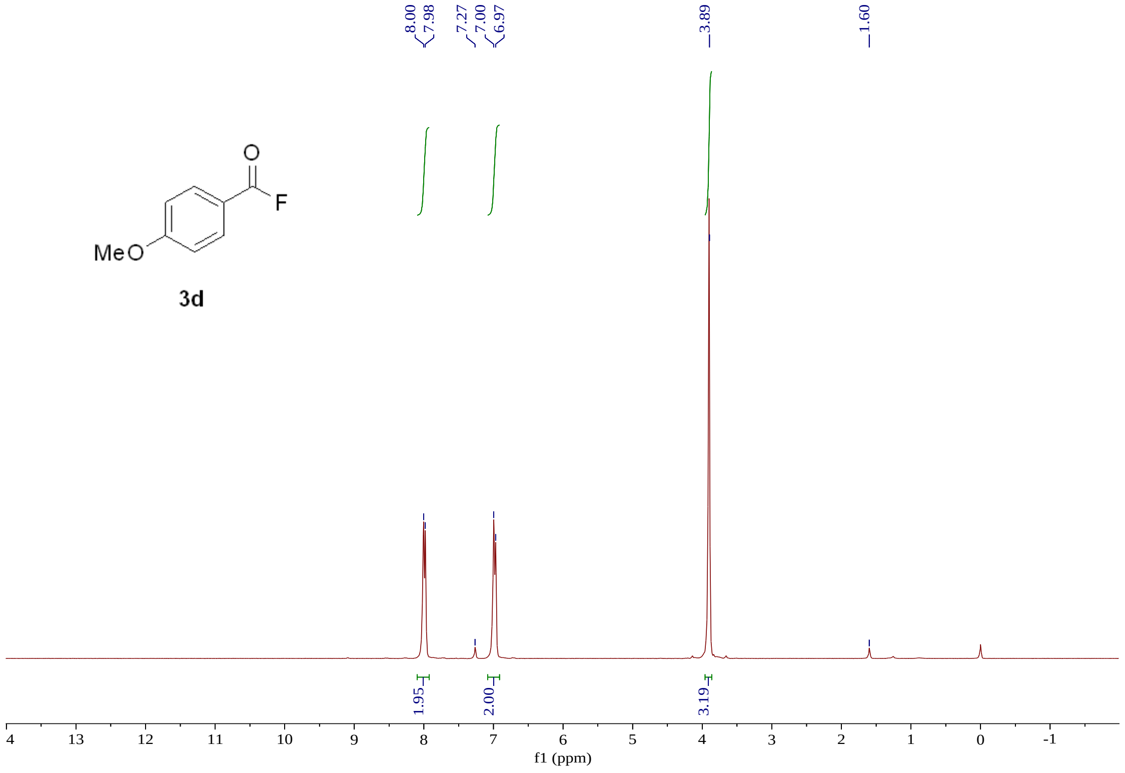


**Supplementary Figure 50.** $^{1}$H NMR Spectra of **3d.**

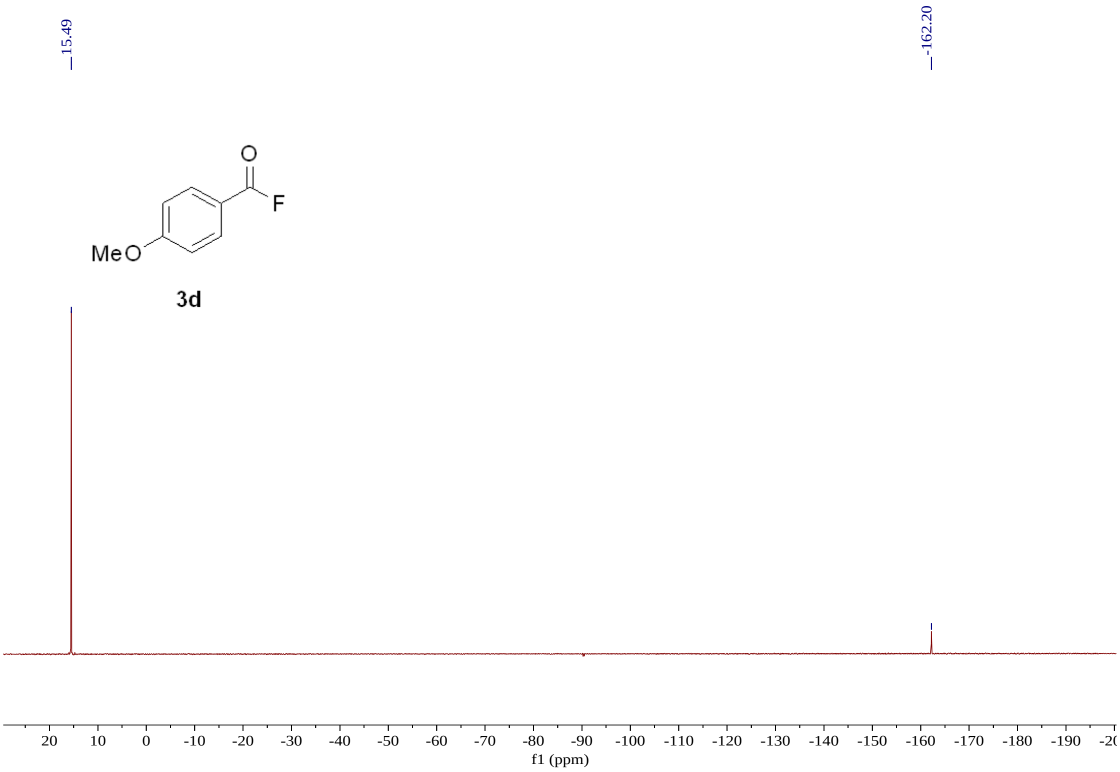


**Supplementary Figure 51.** $^{19}$F NMR Spectra of **3d.**

## Methyl 4-(fluorocarbonyl)benzoate (3e)

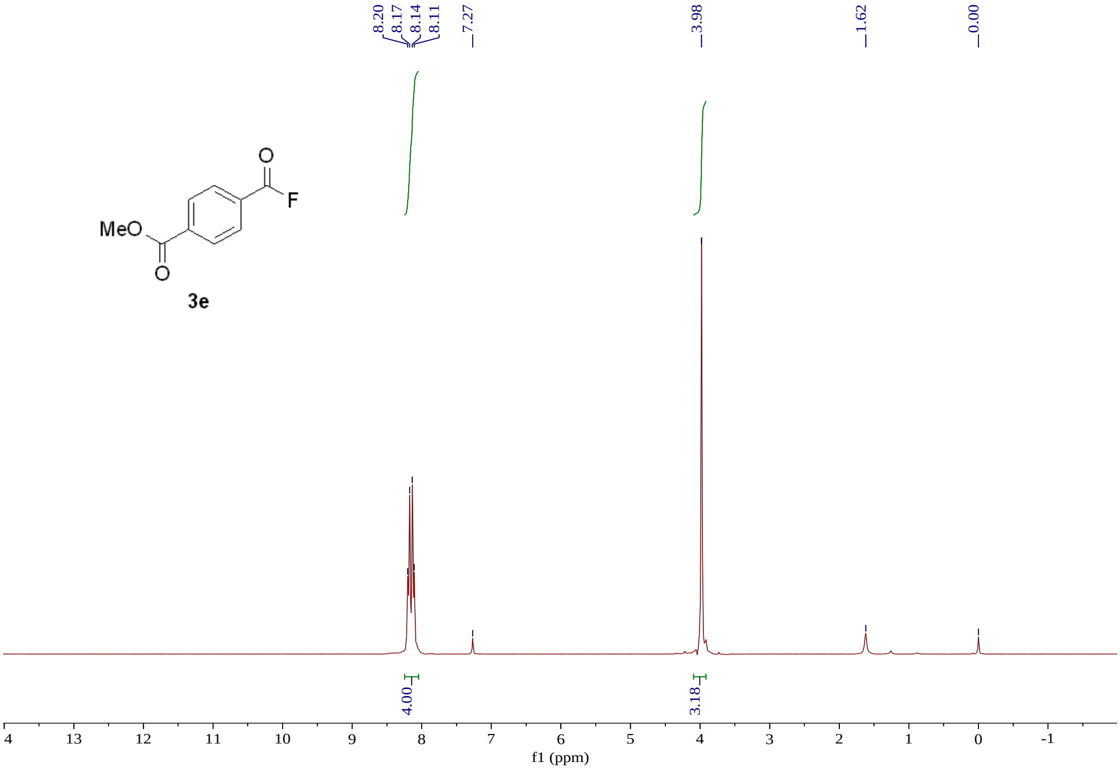


**Supplementary Figure 52.** $^{1}$H NMR Spectra of **3e.**

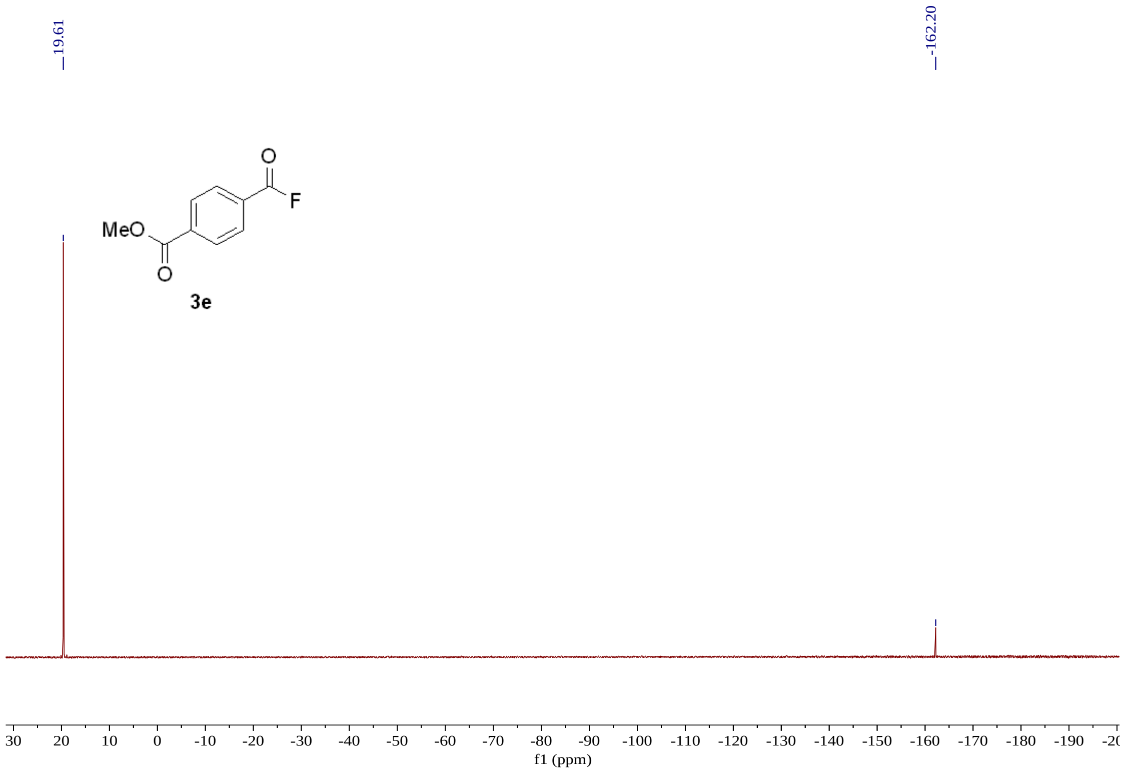


**Supplementary Figure 53.** $^{19}$F NMR Spectra of **3e.**

## 4-(Trifluoromethyl)benzoyl fluoride (3f)

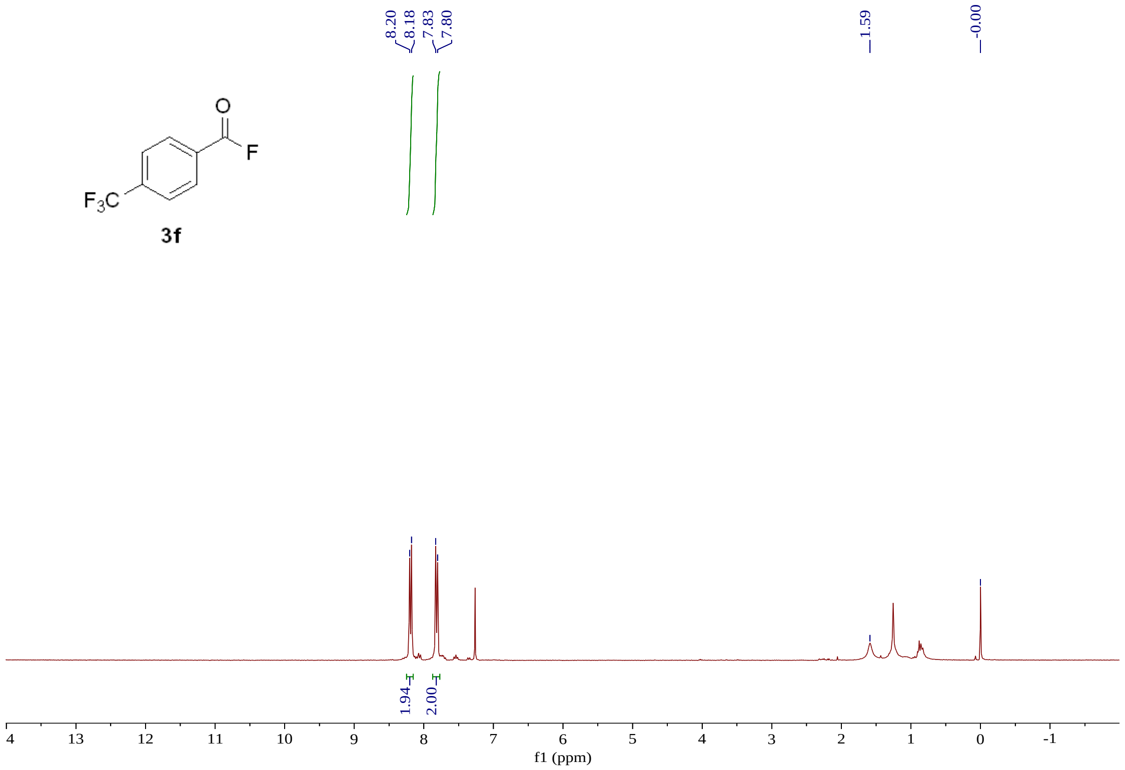


**Supplementary Figure 54.** $^{1}$H NMR Spectra of **3f.**

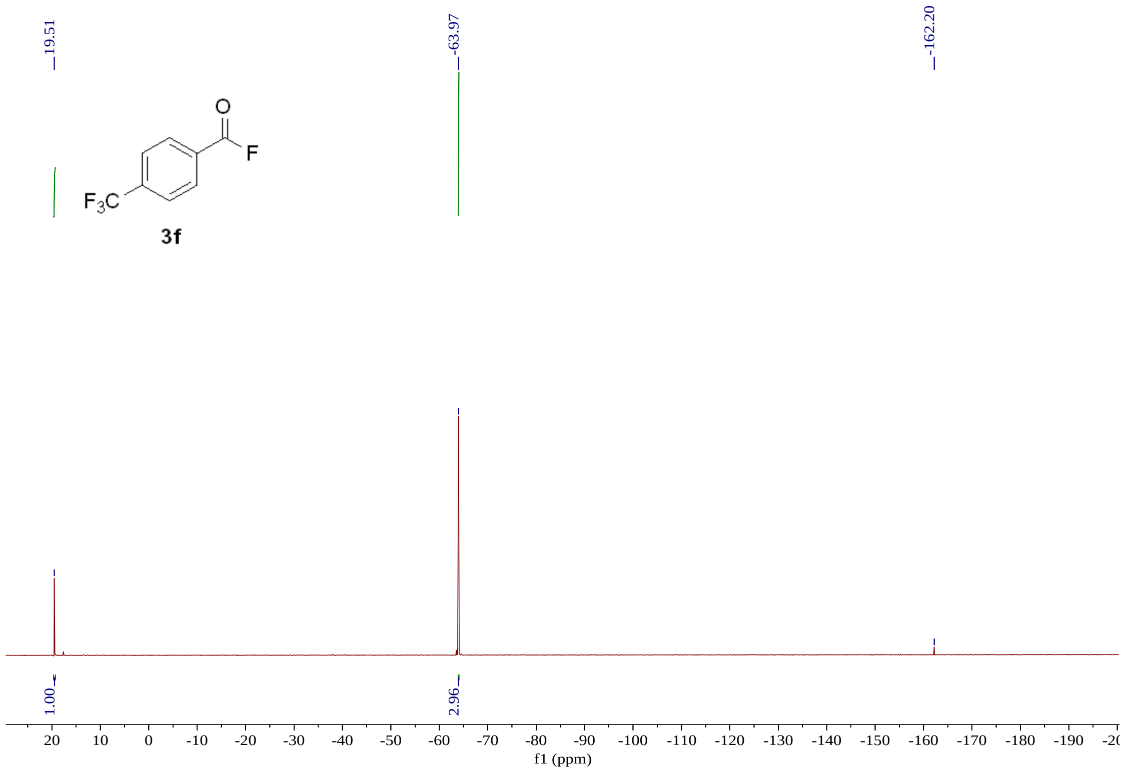


**Supplementary Figure 55.** $^{19}$F NMR Spectra of **3f.**

## 4-Chlorobenzoyl fluoride (3g)

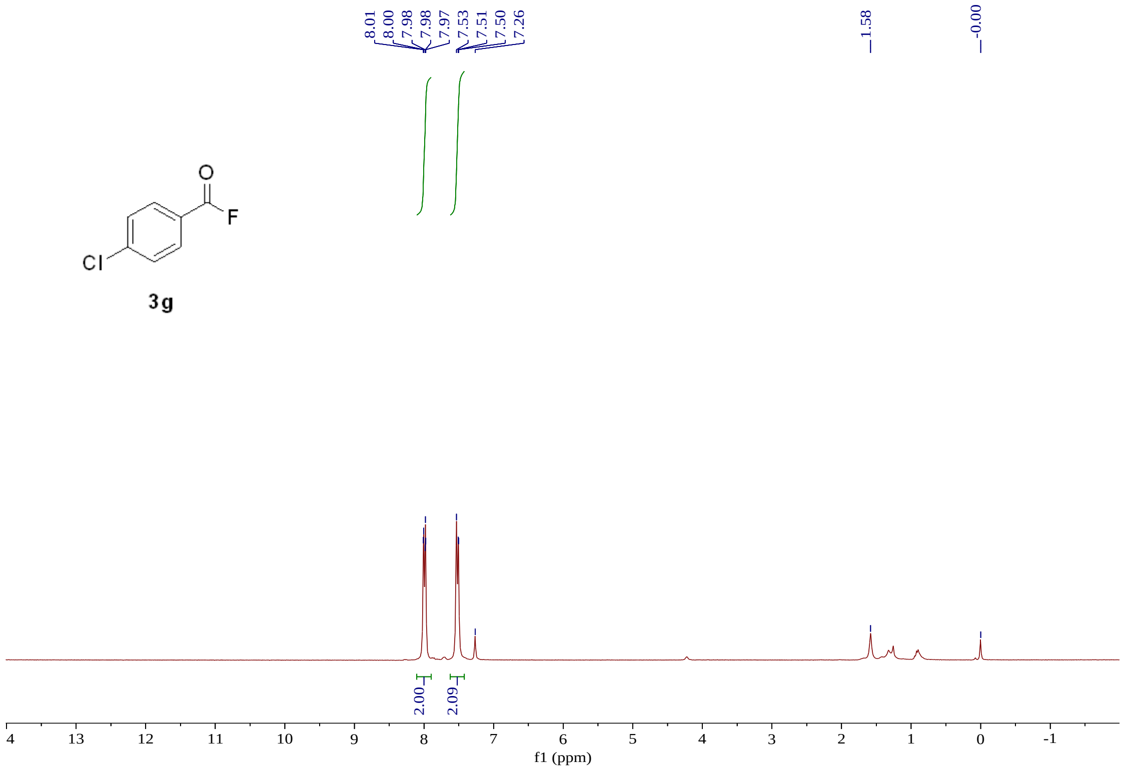


**Supplementary Figure 56.** $^{1}$H NMR Spectra of **3g.**

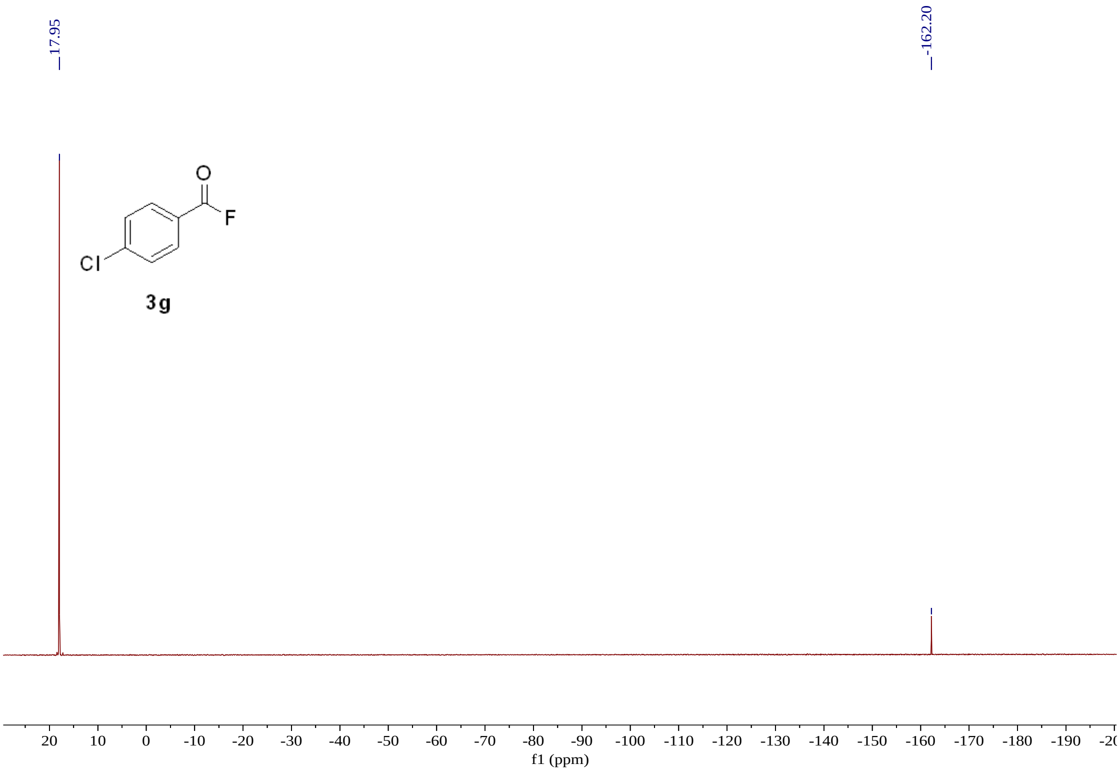


**Supplementary Figure 57.** $^{19}$F NMR Spectra of **3g.**

## 4-Bromobenzoyl fluoride (3h)

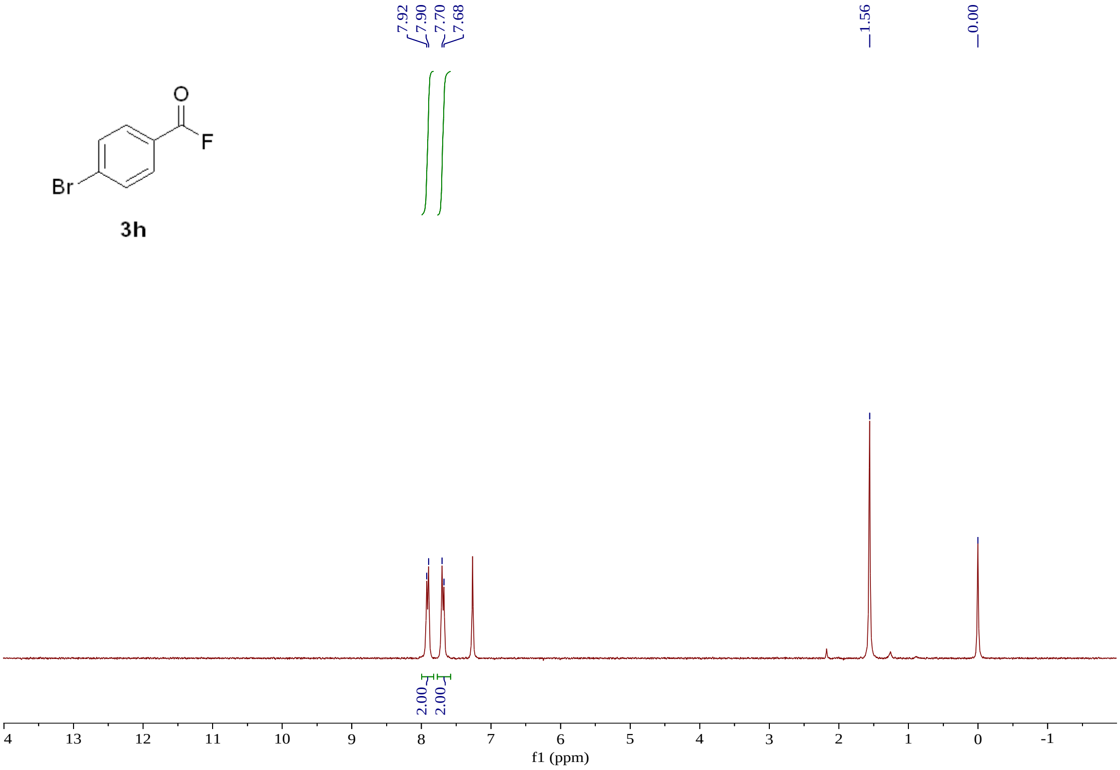


**Supplementary Figure 58.** $^{1}$H NMR Spectra of **3h.**

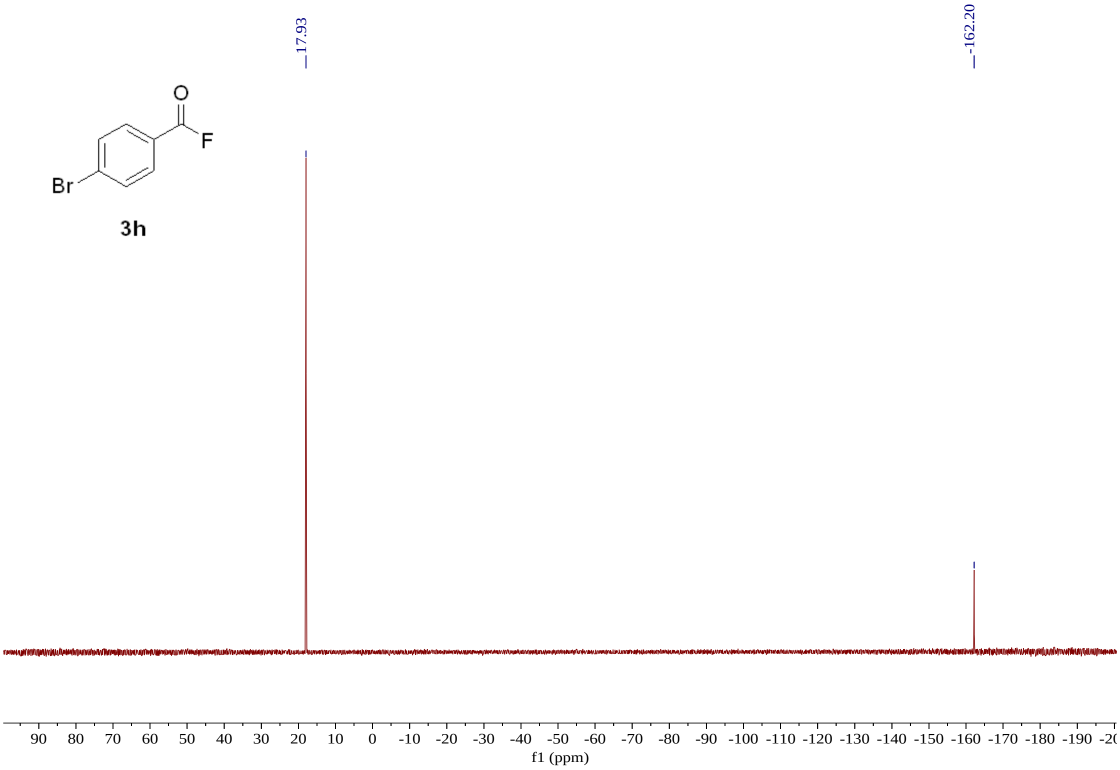


**Supplementary Figure 59.** $^{19}$F NMR Spectra of **3h.**

**4-Fluorobenzoyl fluoride (3i)**

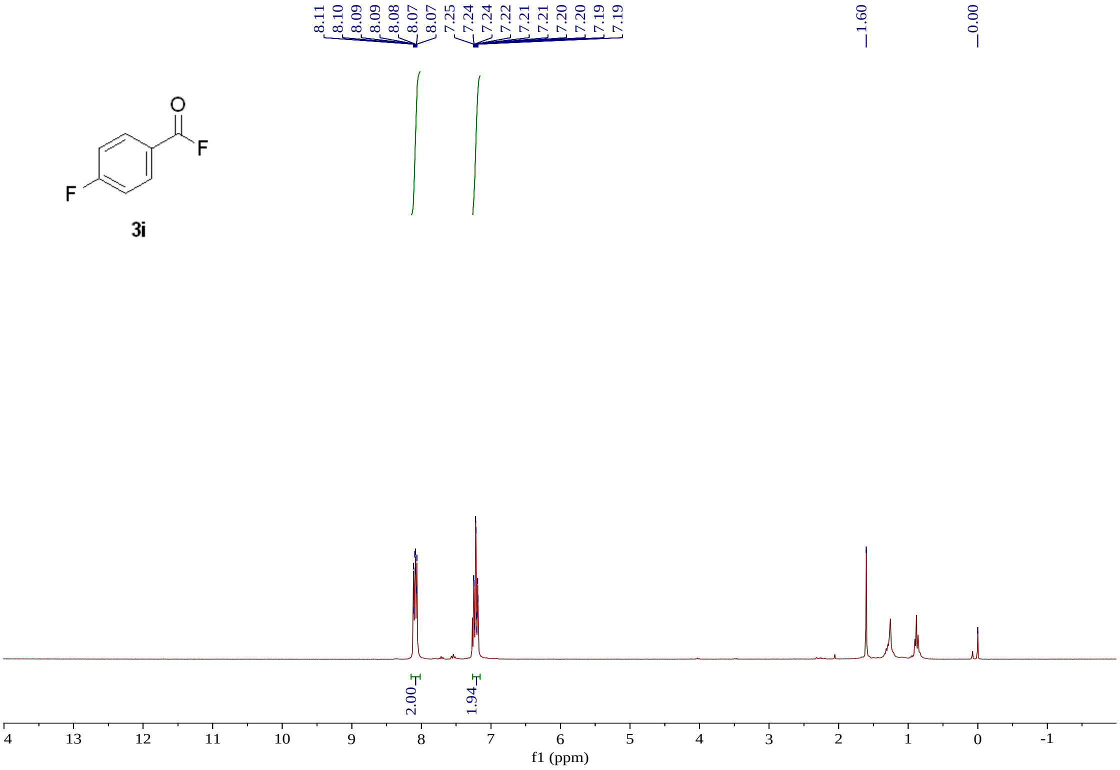


**Supplementary Figure 60.** $^1$H NMR Spectra of **3i.**

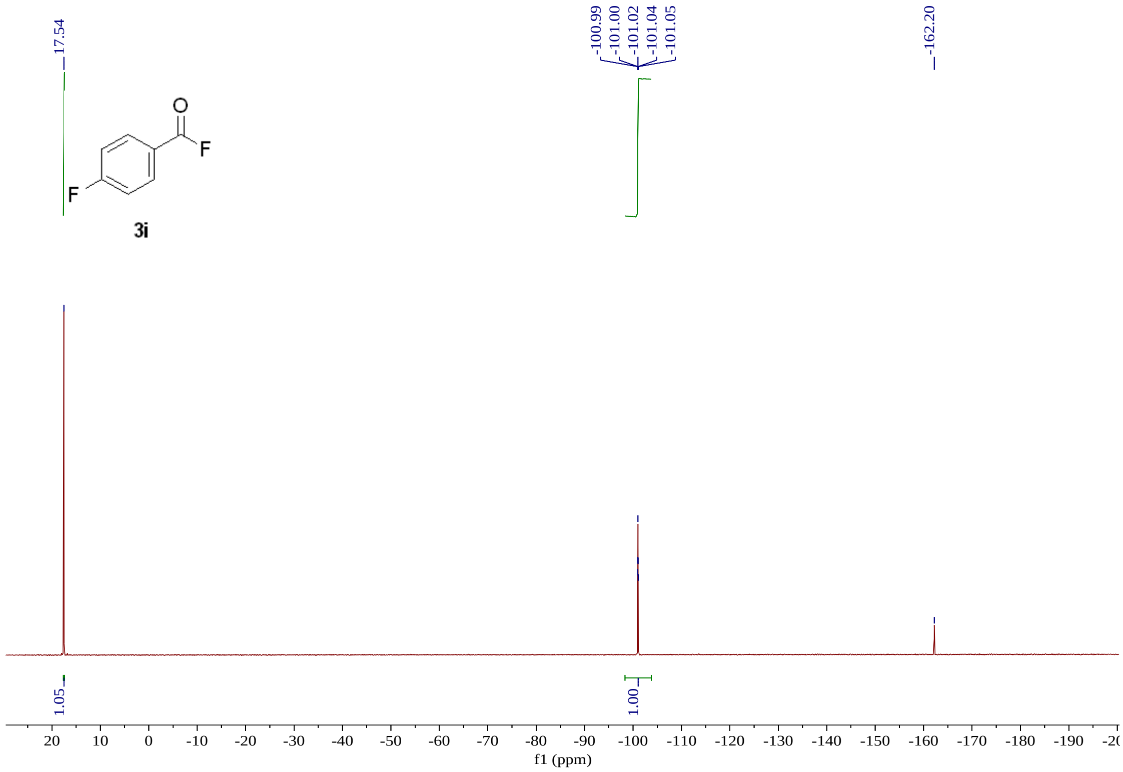


**Supplementary Figure 61.** $^{19}$F NMR Spectra of **3i.**

## 4-Fluorobenzoyl fluoride (3j)

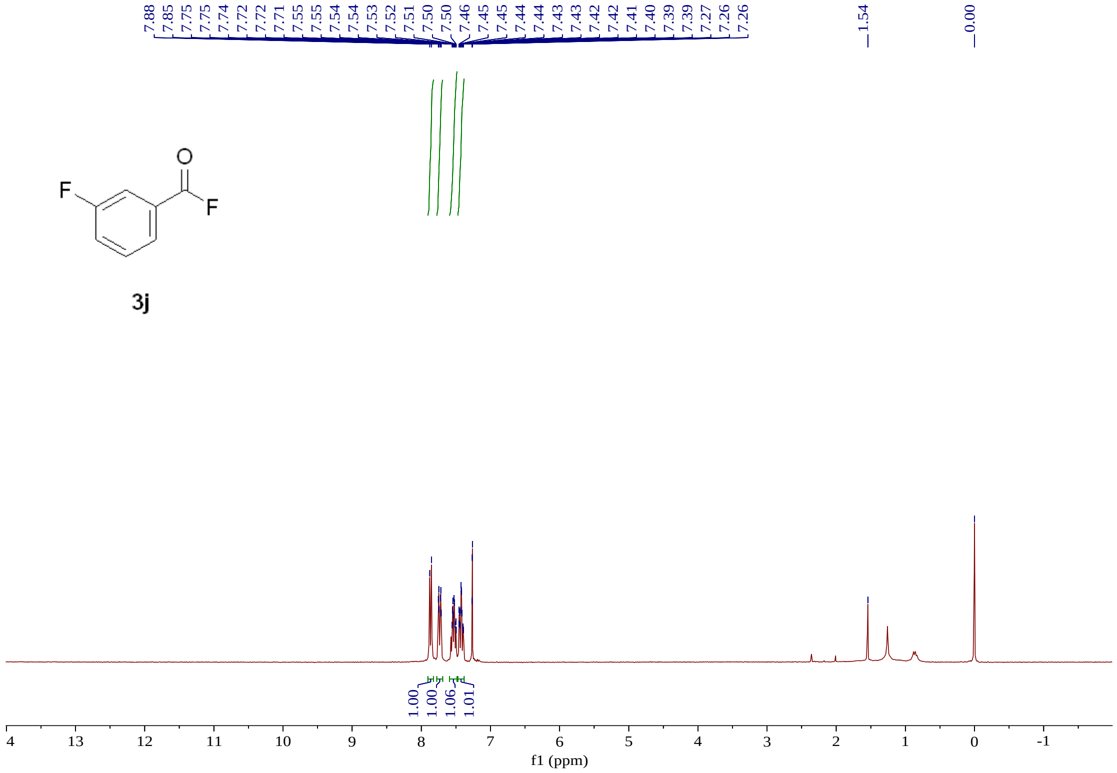


**Supplementary Figure 62.** $^{1}$H NMR Spectra of **3j.**

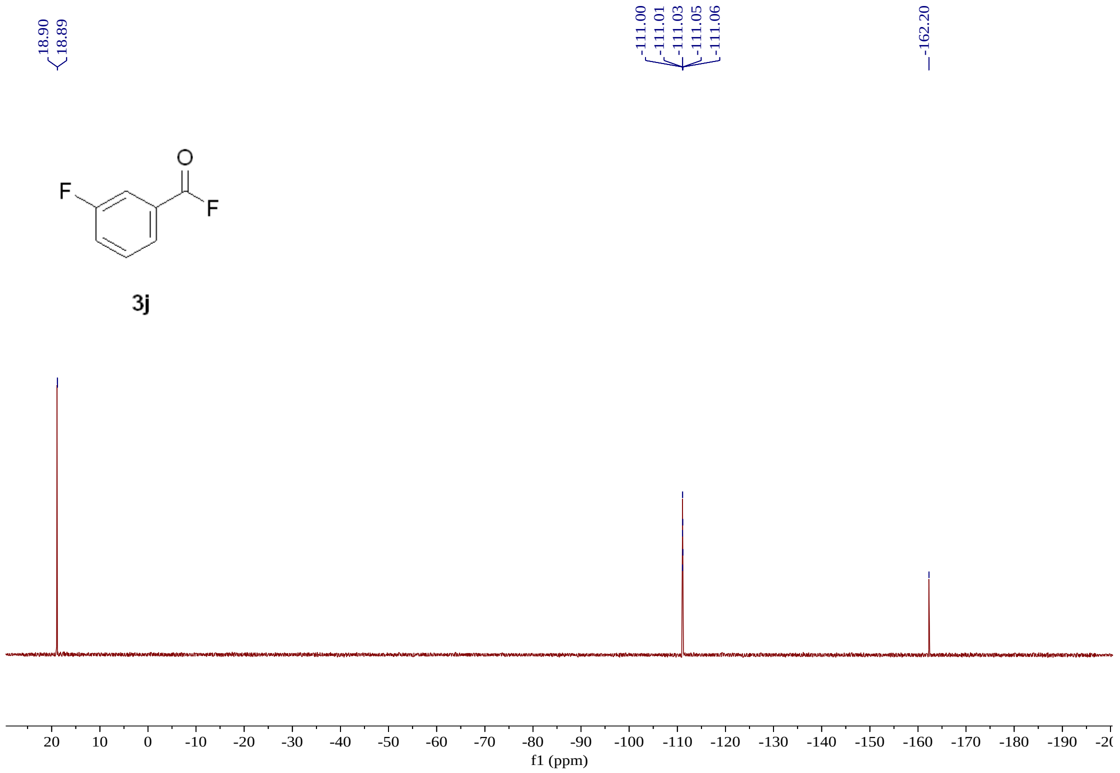


**Supplementary Figure 63.** $^{19}$F NMR Spectra of **3j.**

## 3-Methylbenzoyl fluoride (3k)

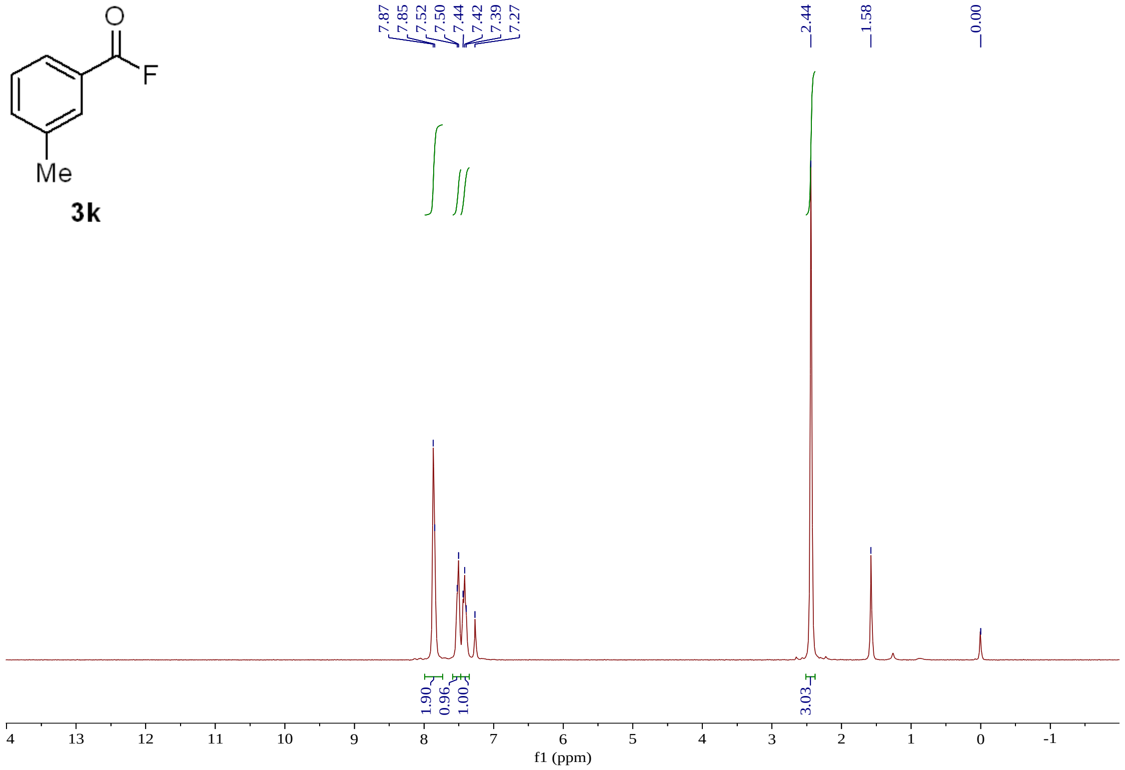


**Supplementary Figure 64.** $^{1}$H NMR Spectra of **3k.**

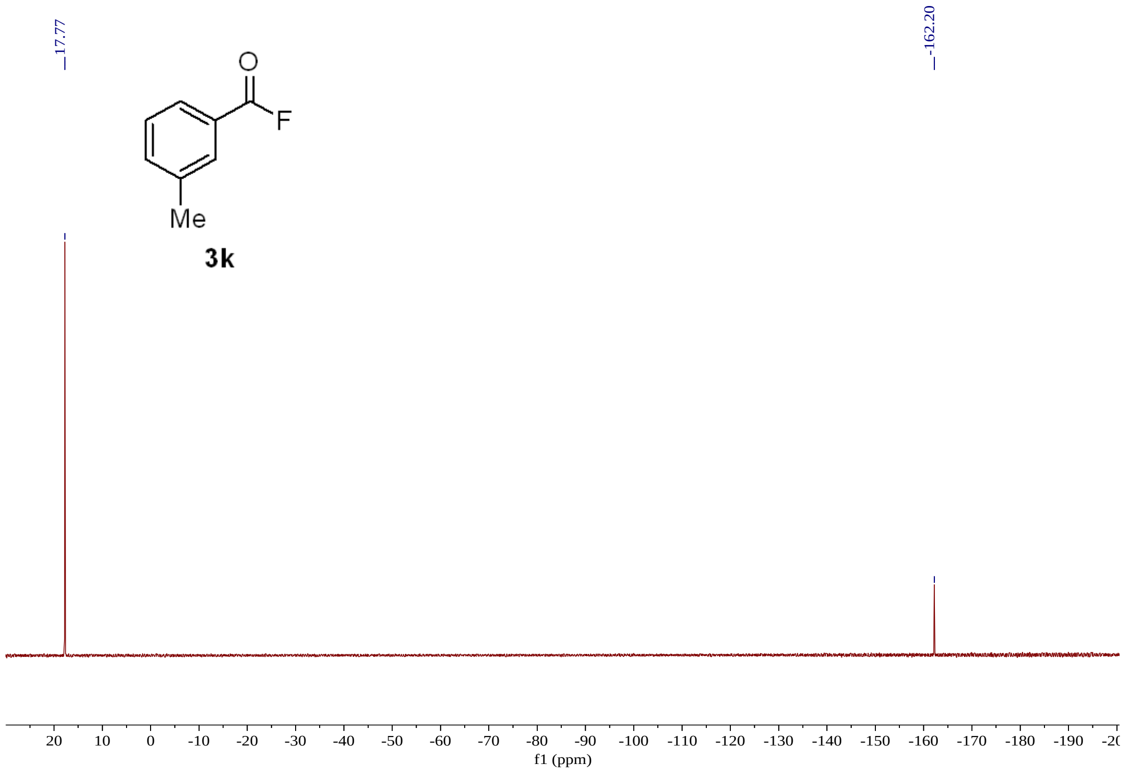

**Supplementary Figure 65.** $^{19}$F NMR Spectra of **3k.**

**2-Methylbenzoyl fluoride (3l)**

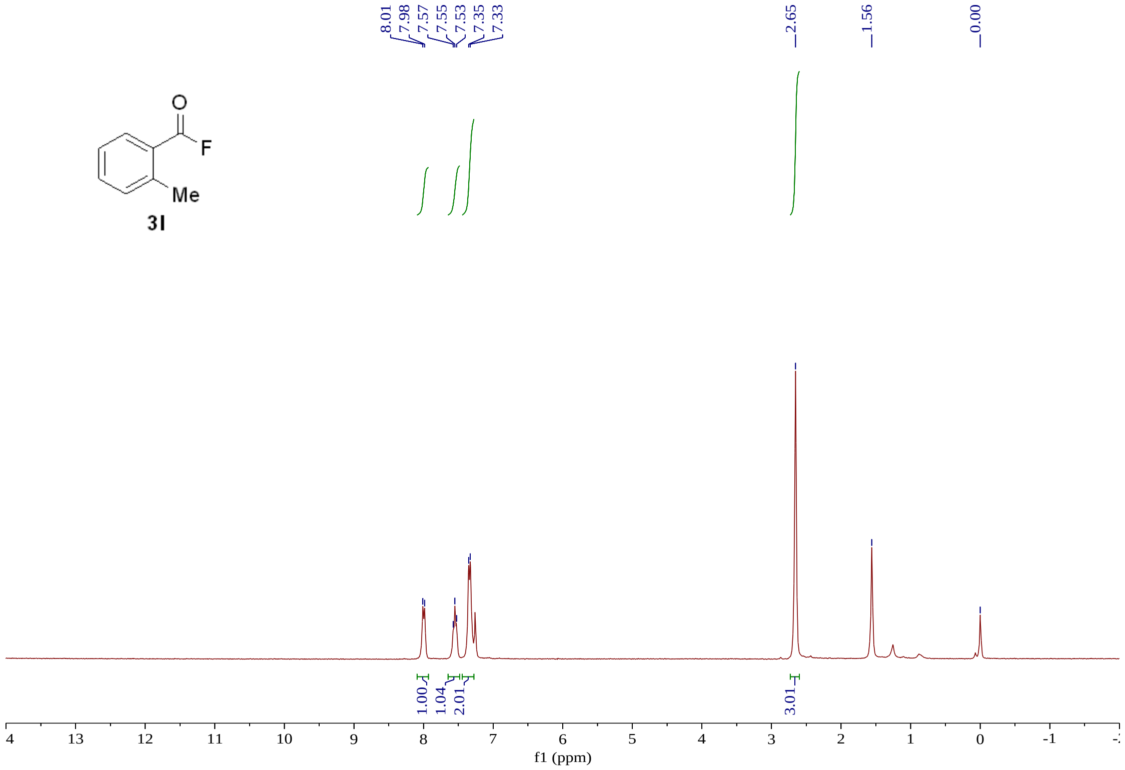

**Supplementary Figure 66.** $^{1}$H NMR Spectra of **3l.**

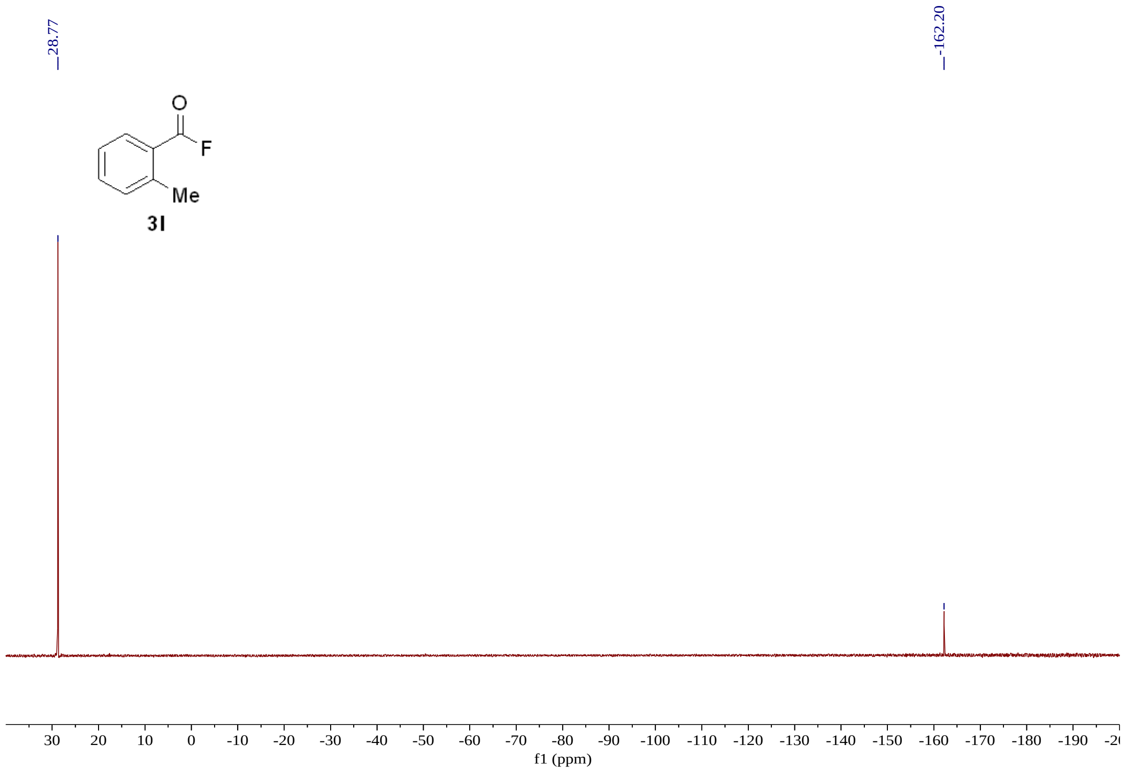


**Supplementary Figure 67.** $^{19}$F NMR Spectra of **3l.**

## 2-Naphthoyl fluoride (3m)

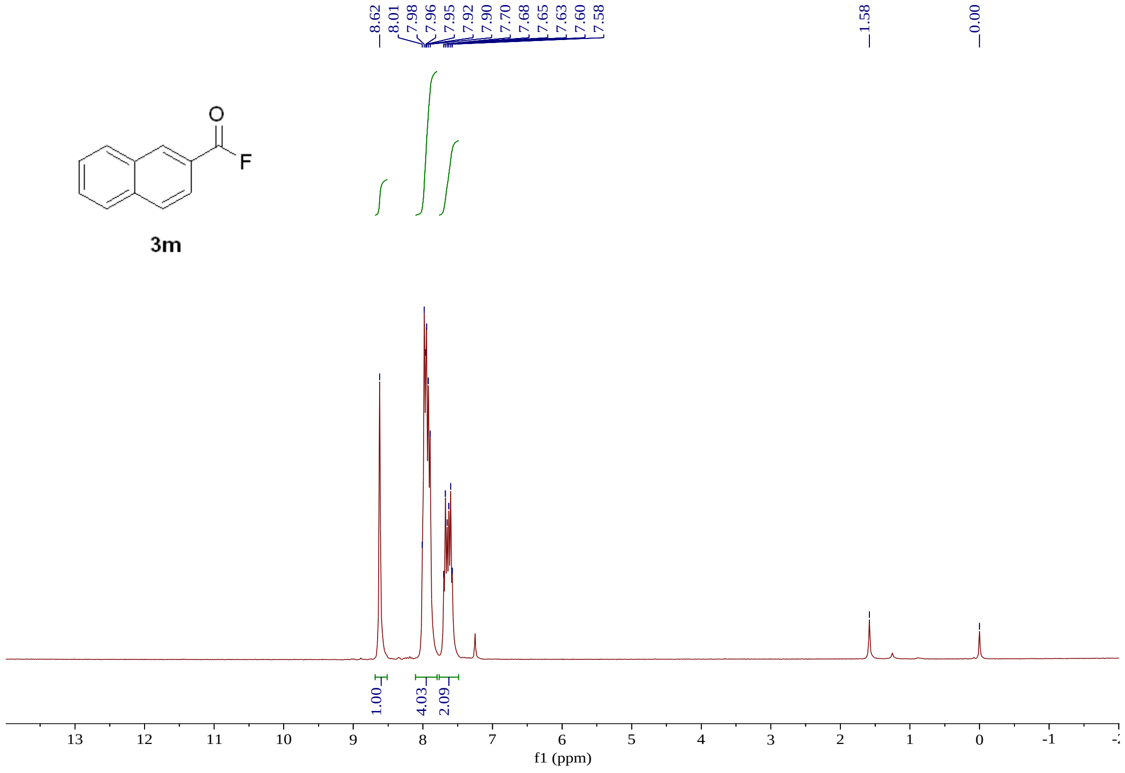


**Supplementary Figure 68.** $^{1}$H NMR Spectra of **3m.**

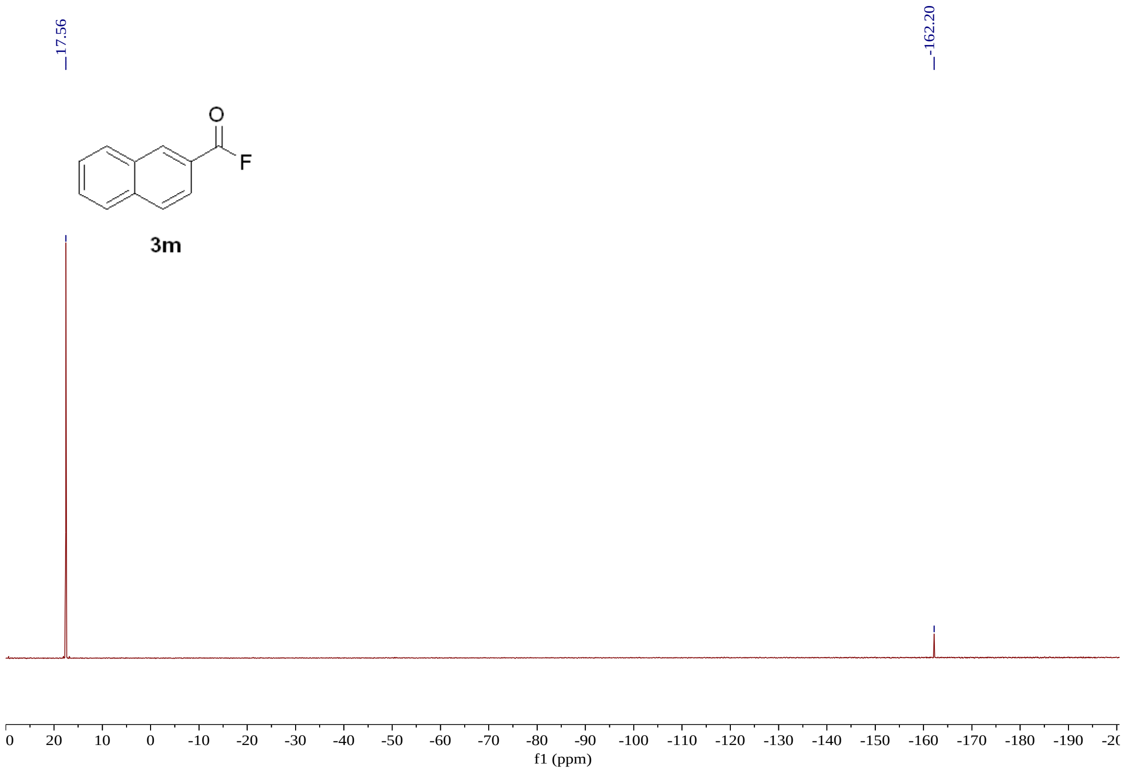

Supplementary Figure 69. $^{19}$F NMR Spectra of **3m.**

**1-Naphthoyl fluoride (3n)**

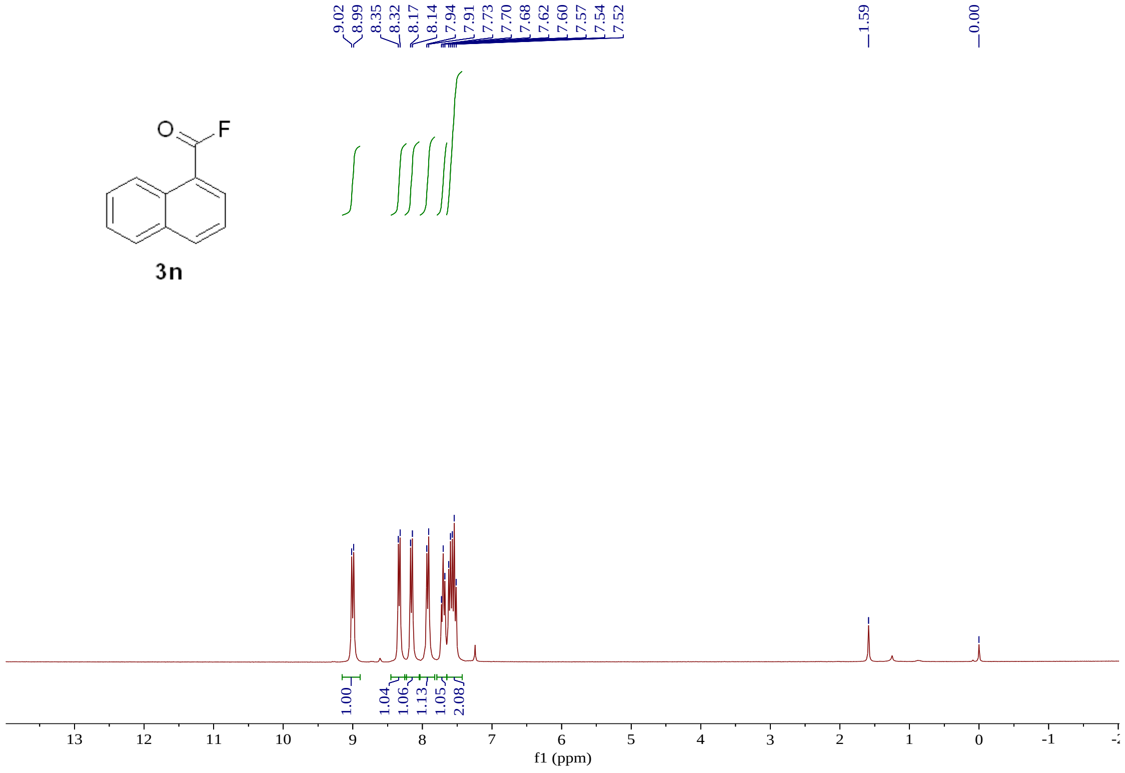

**Supplementary Figure 70.** $^{1}$H NMR Spectra of **3n.**

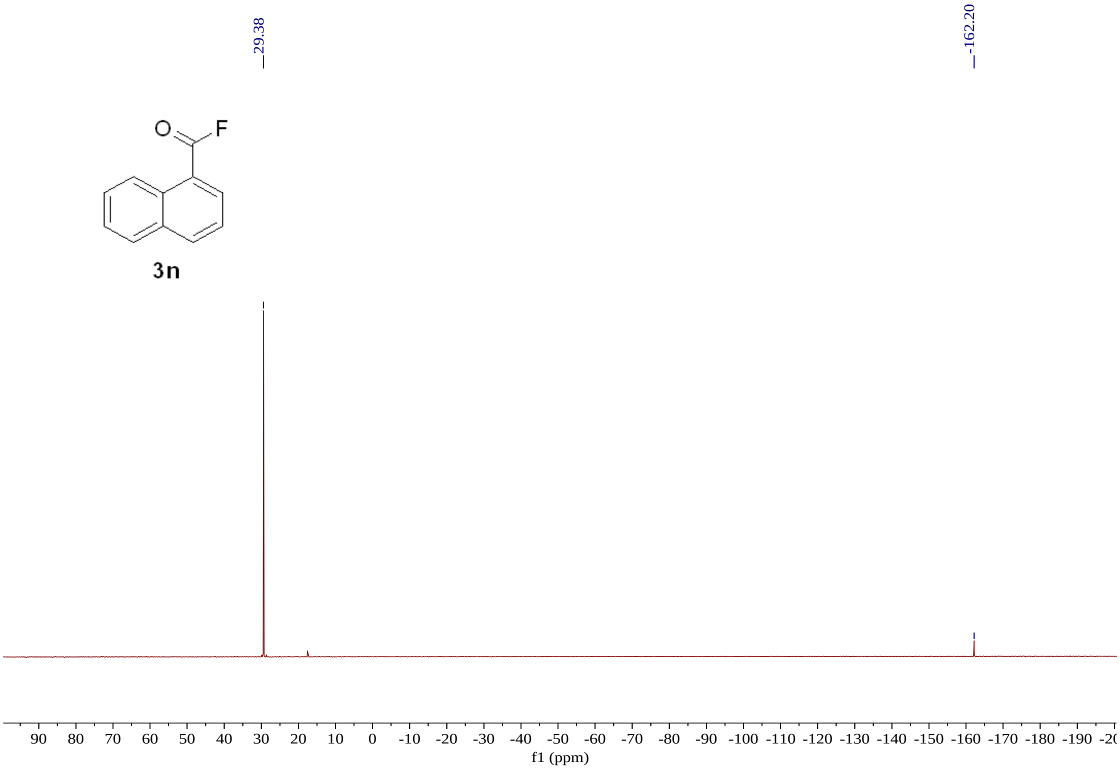


**Supplementary Figure 71.** $^{19}$F NMR Spectra of **3n.**

**Cinnamoyl fluoride (3o)**

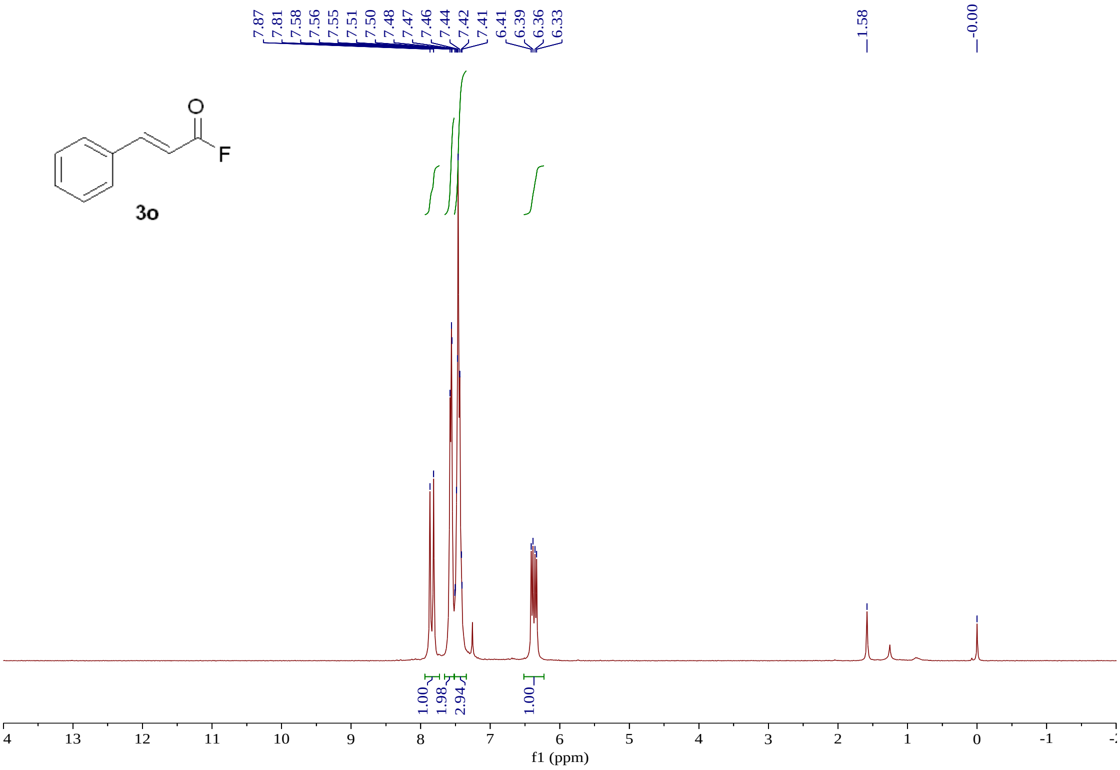


**Supplementary Figure 72.** $^{1}$H NMR Spectra of **3o.**

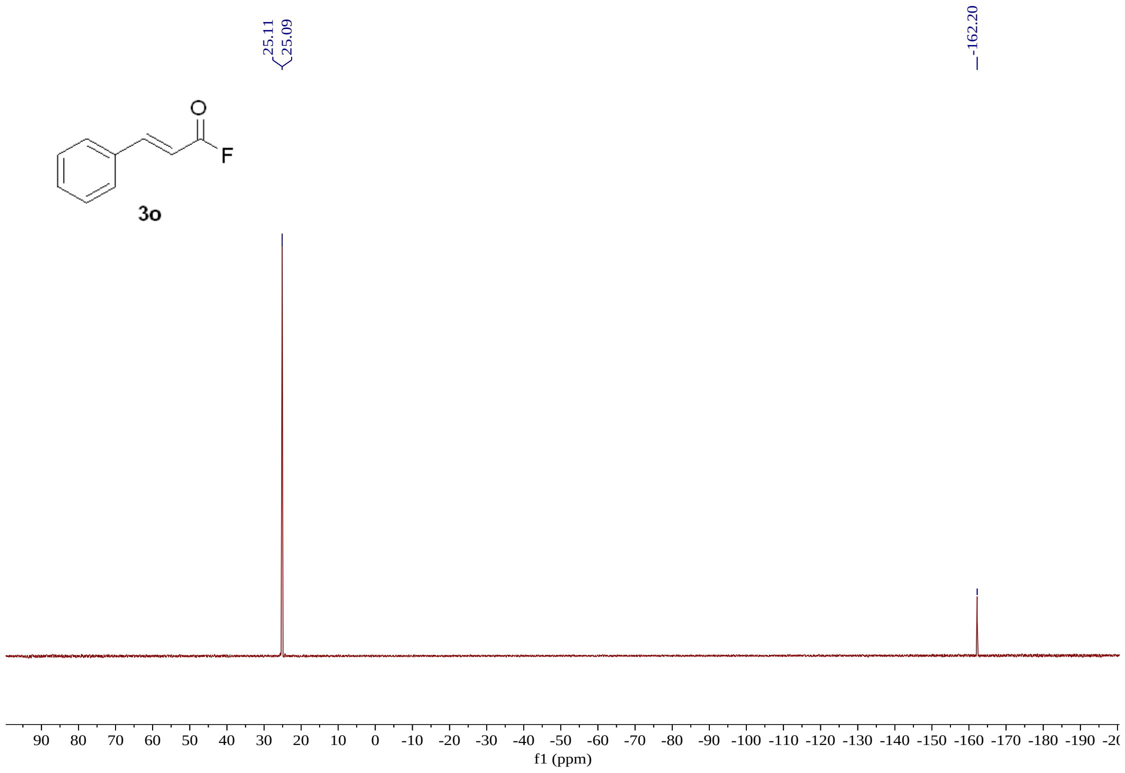

**Supplementary Figure 73.** $^{19}$F NMR Spectra of **3o.**

**_(E)_-3-(4-Methoxyphenyl)acryloyl fluoride (3p)**

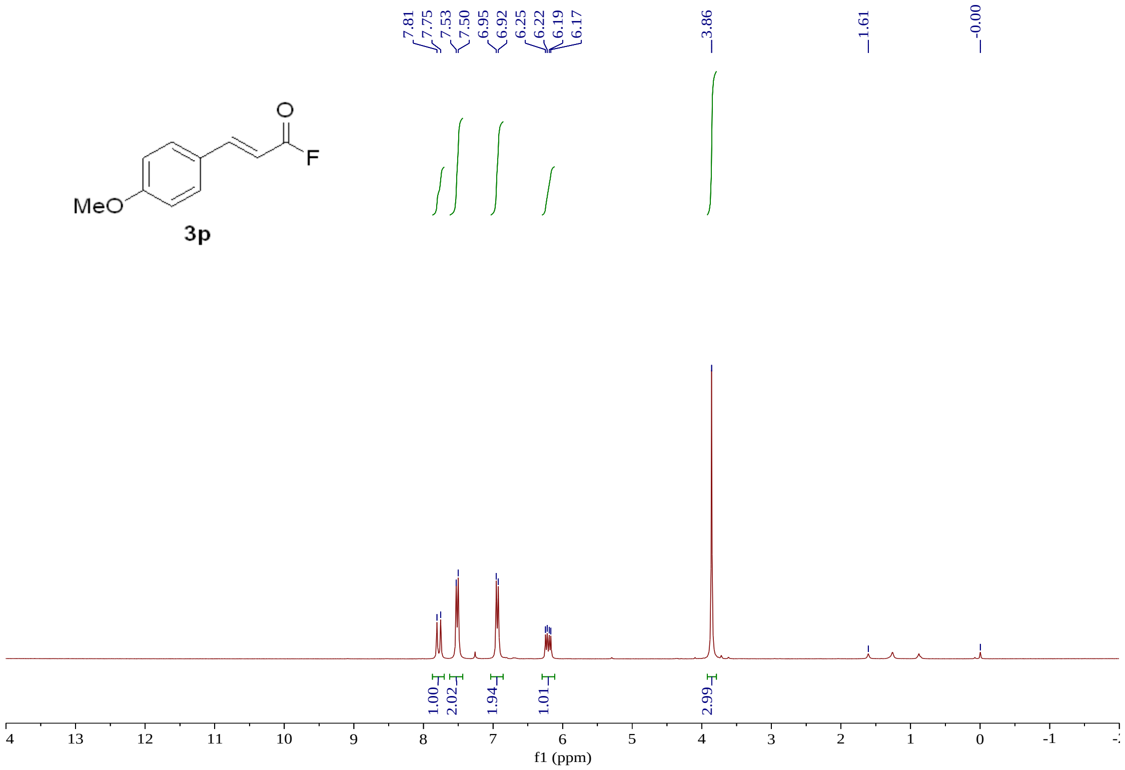

**Supplementary Figure 74.** $^{1}$H NMR Spectra of **3.**

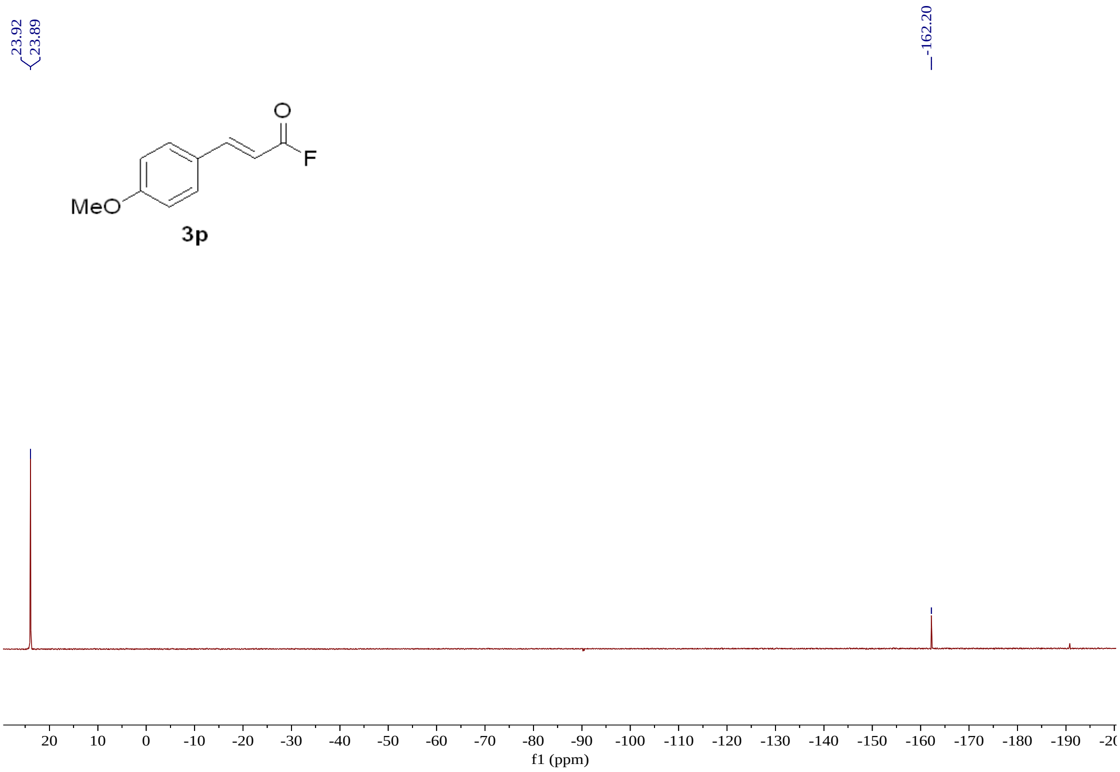


**Supplementary Figure 75.** $^{19}$F NMR Spectra of **3p.**

## Thiophene-2-carbonyl fluoride (3q)

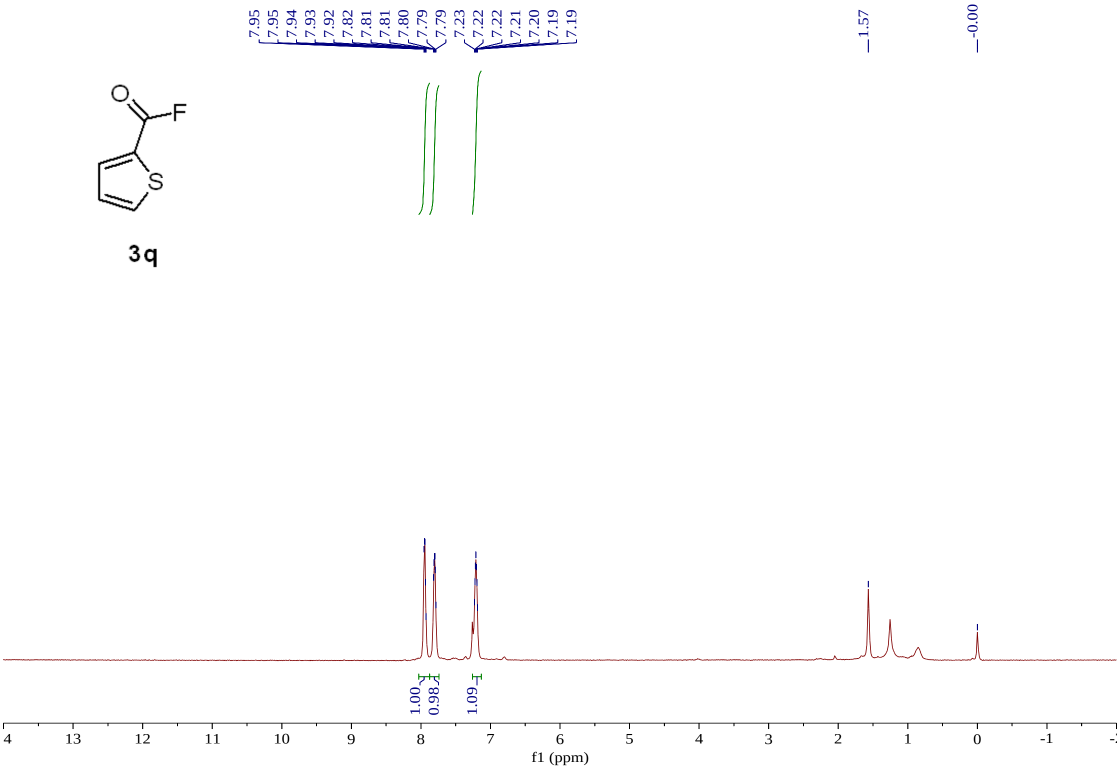


**Supplementary Figure 76.** $^{1}$H NMR Spectra of **3q.**

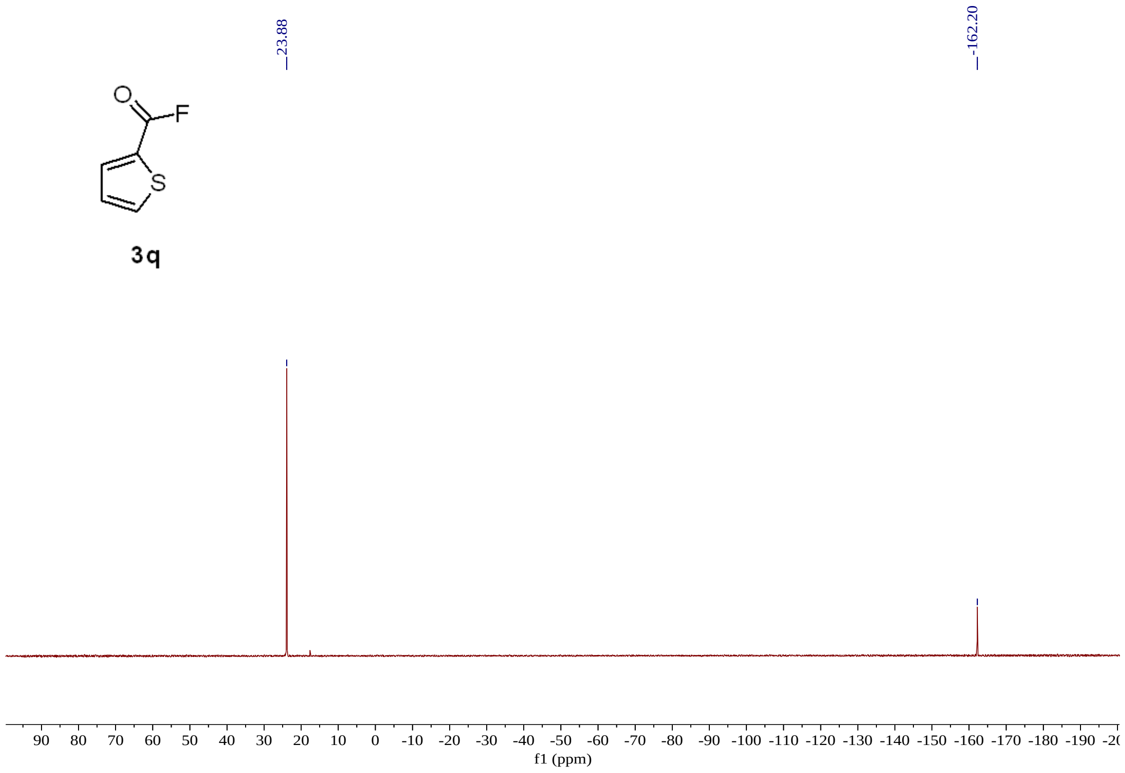


**Supplementary Figure 77.** $^{19}$F NMR Spectra of **3q.**

**Benzo[*b*]thiophene-2-carbonyl fluoride (3r)**

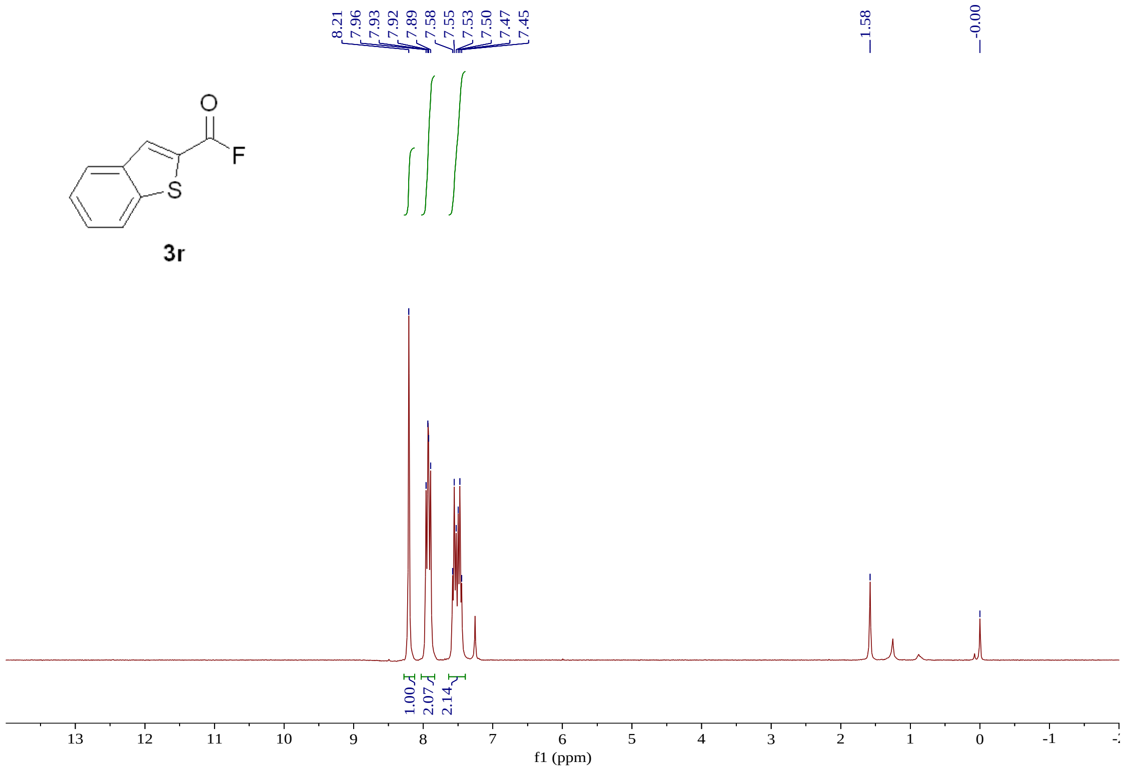


**Supplementary Figure 78.** $^{1}$H NMR Spectra of **3r.**

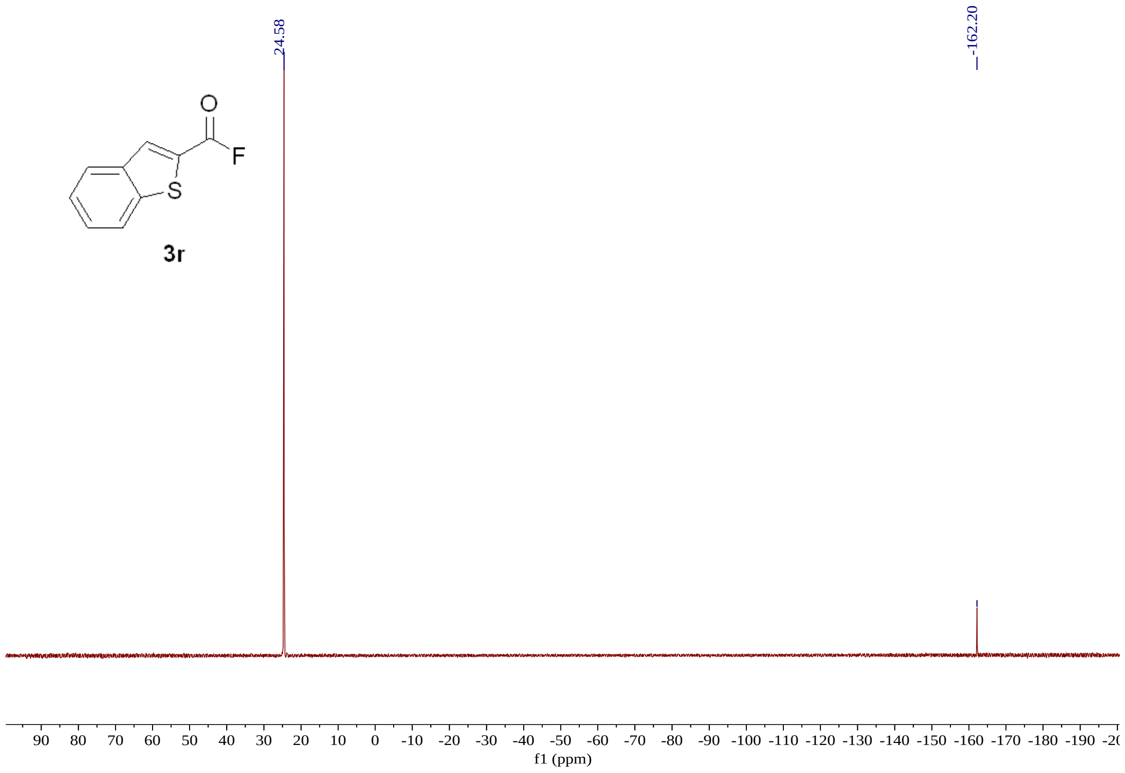

**Supplementary Figure 79.** $^{19}$F NMR Spectra of **3r.**

**4-((1,3-Dioxoisoindolin-2-yl)methyl)benzoyl fluoride (3s)**

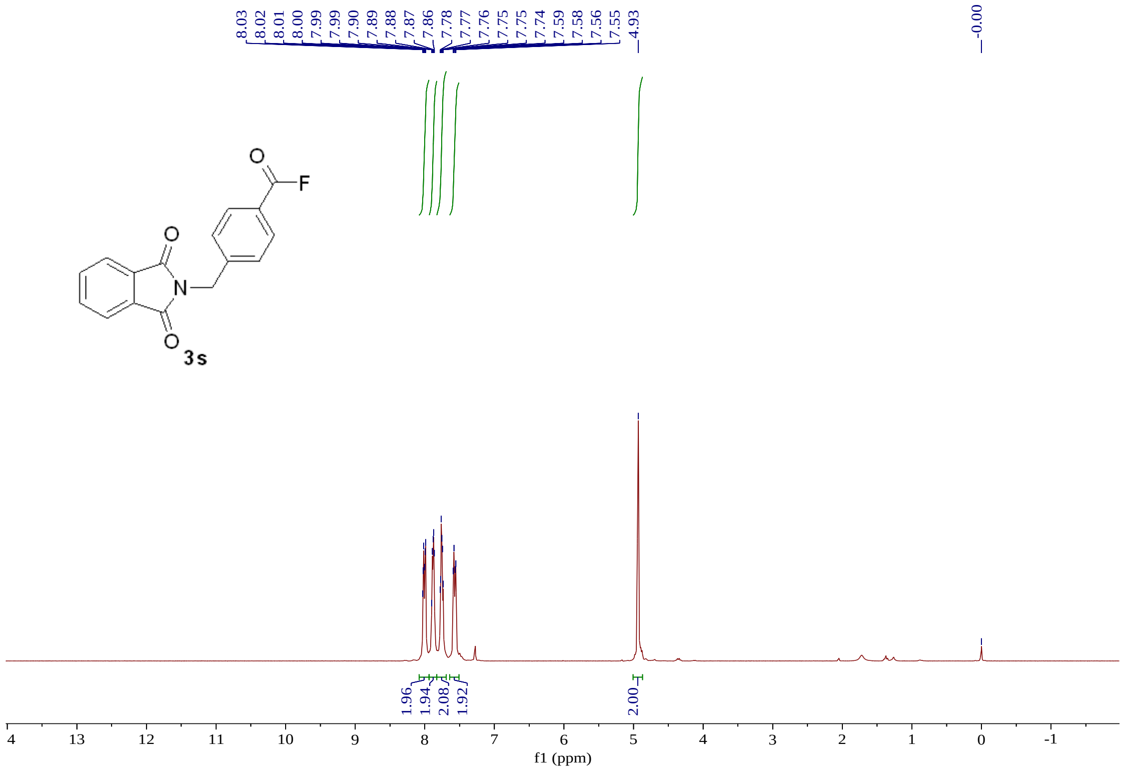

**Supplementary Figure 80.** $^{1}$H NMR Spectra of **3s.**

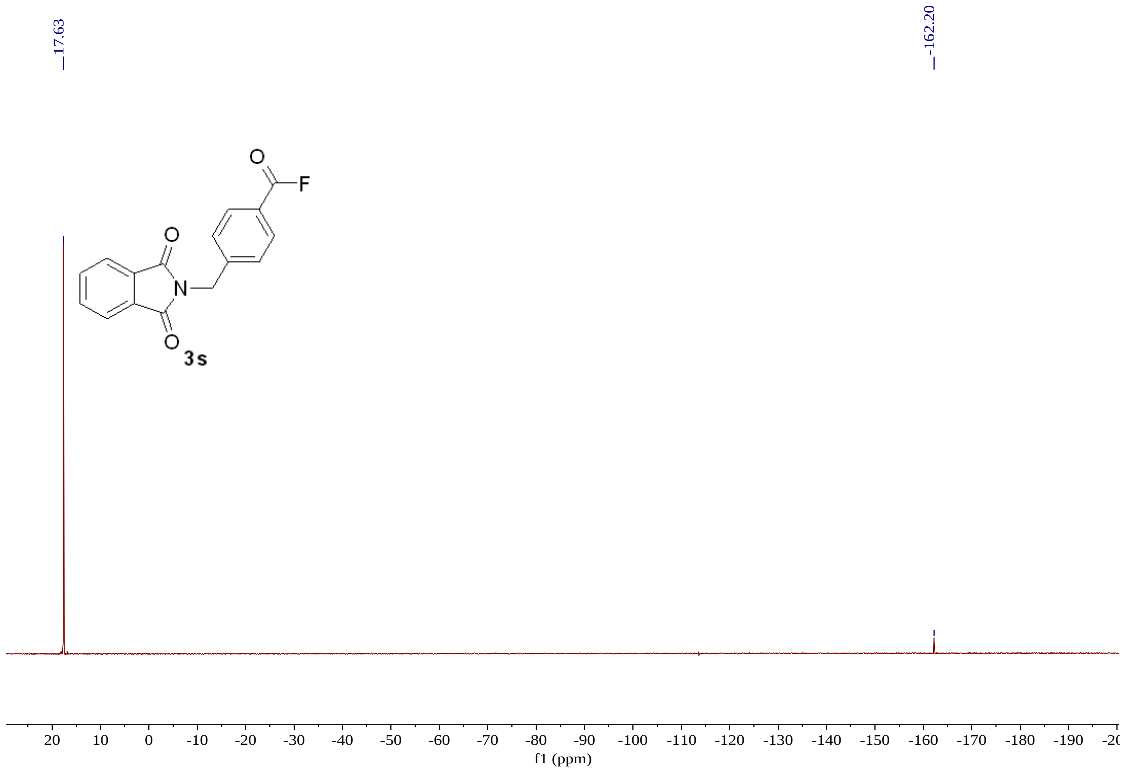


**Supplementary Figure 81.** $^{19}$F NMR Spectra of **3s.**

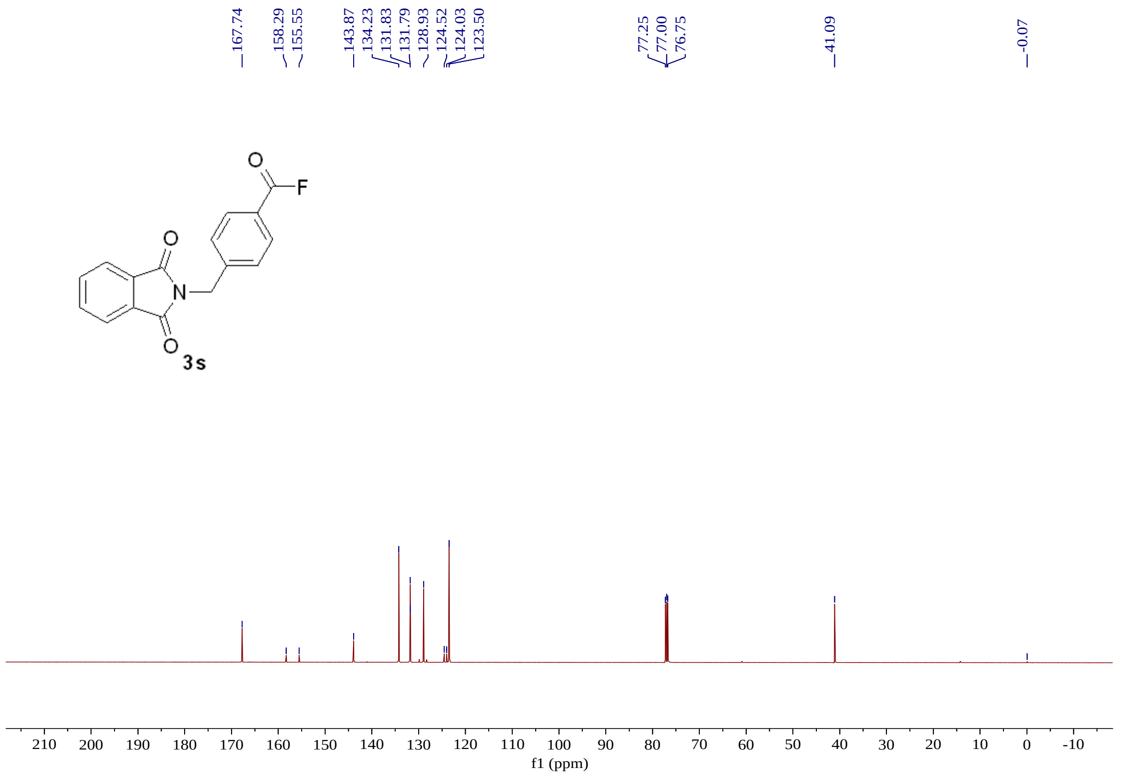


**Supplementary Figure 82.** $^{13}$C NMR Spectra of **3s.**

**1-Benzyl-1*H*-pyrazole-4-carbonyl fluoride (3t)**

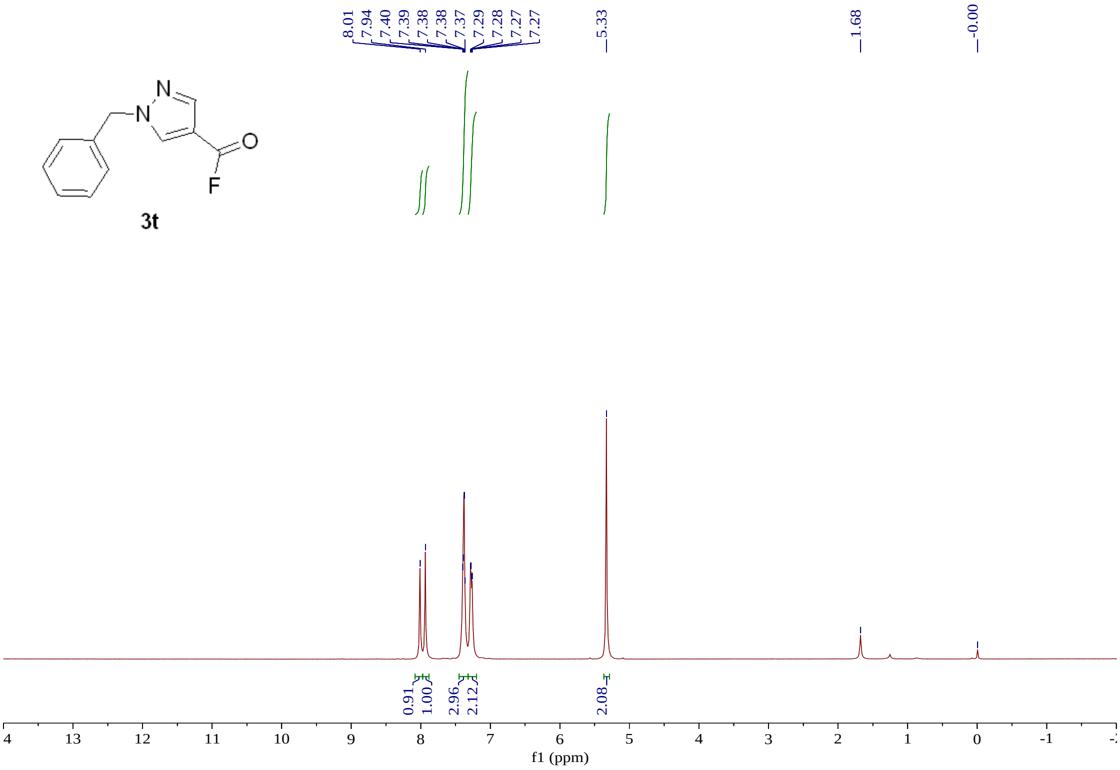


**Supplementary Figure 83.** $^{1}$H NMR Spectra of **3t.**

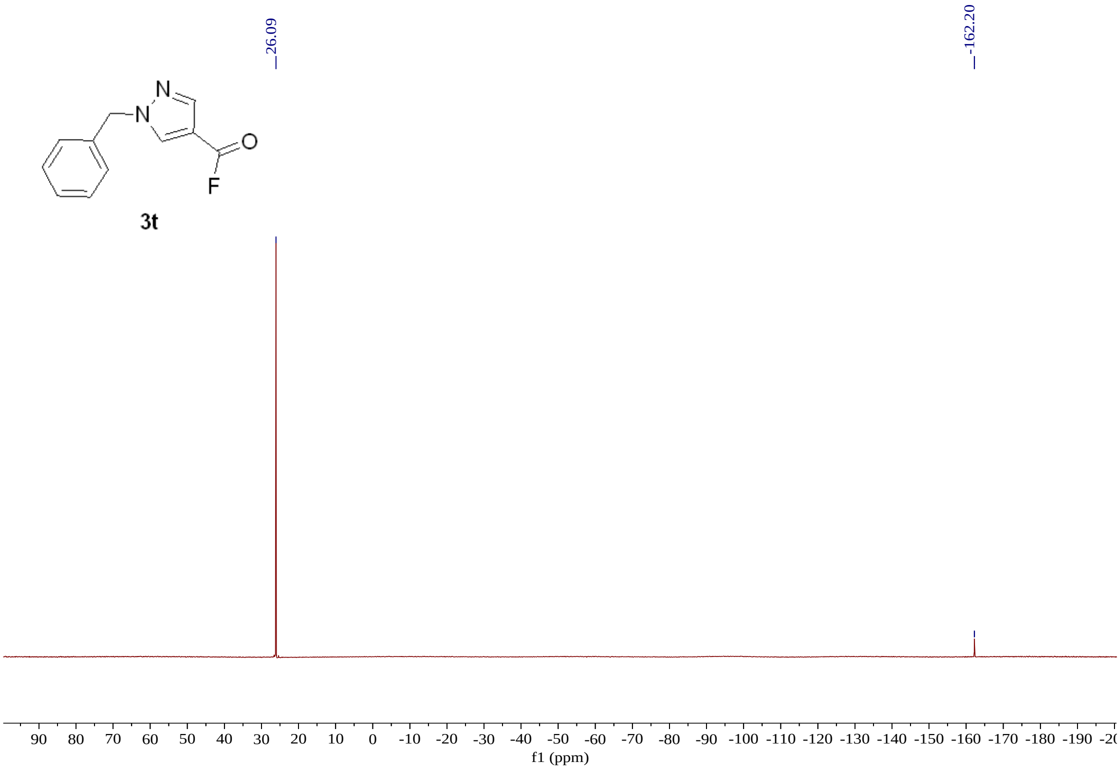


**Supplementary Figure 84.** $^{19}$F NMR Spectra of **3t.**

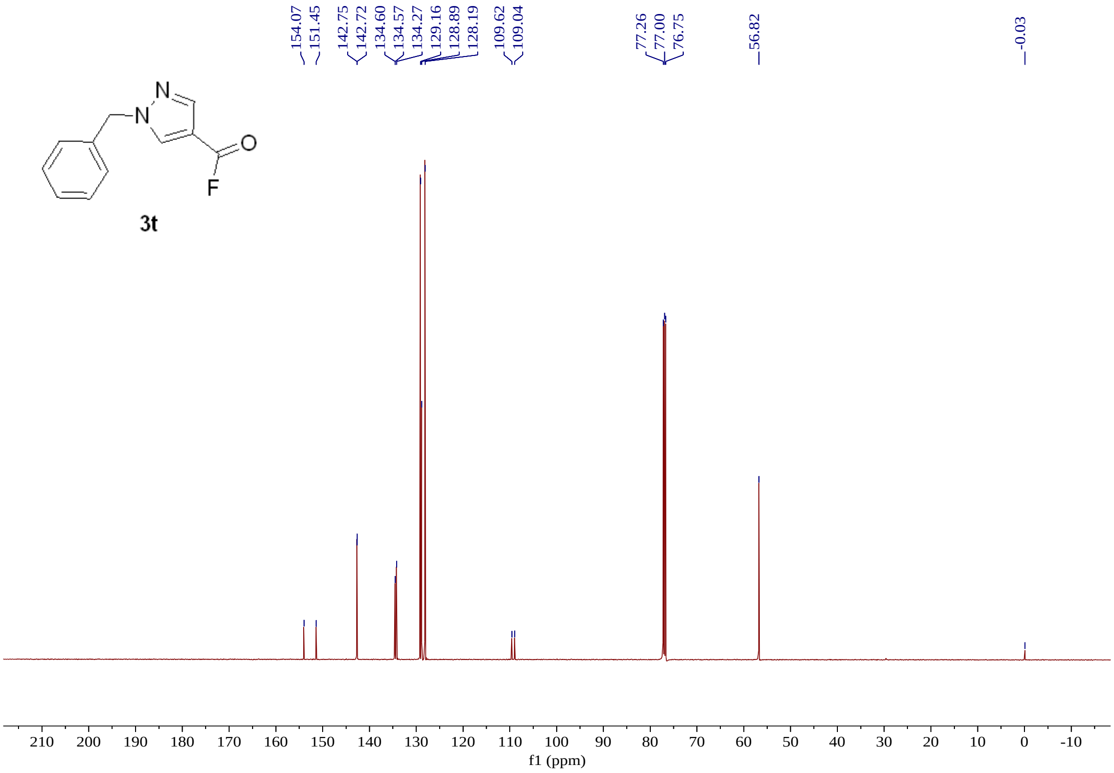


**Supplementary Figure 85.** $^{13}$C NMR Spectra of **3t.**

**2-Phenylacryloyl fluoride (3u)**

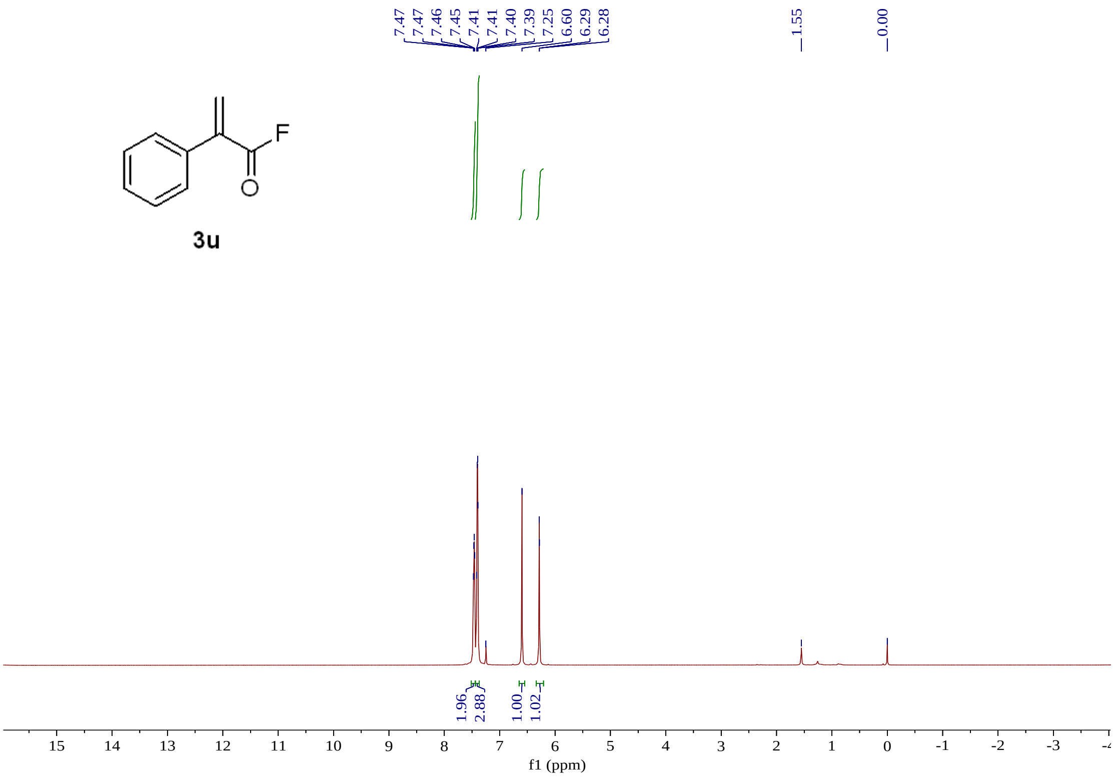


**Supplementary Figure 86.** $^{1}$H NMR Spectra of **3u.**

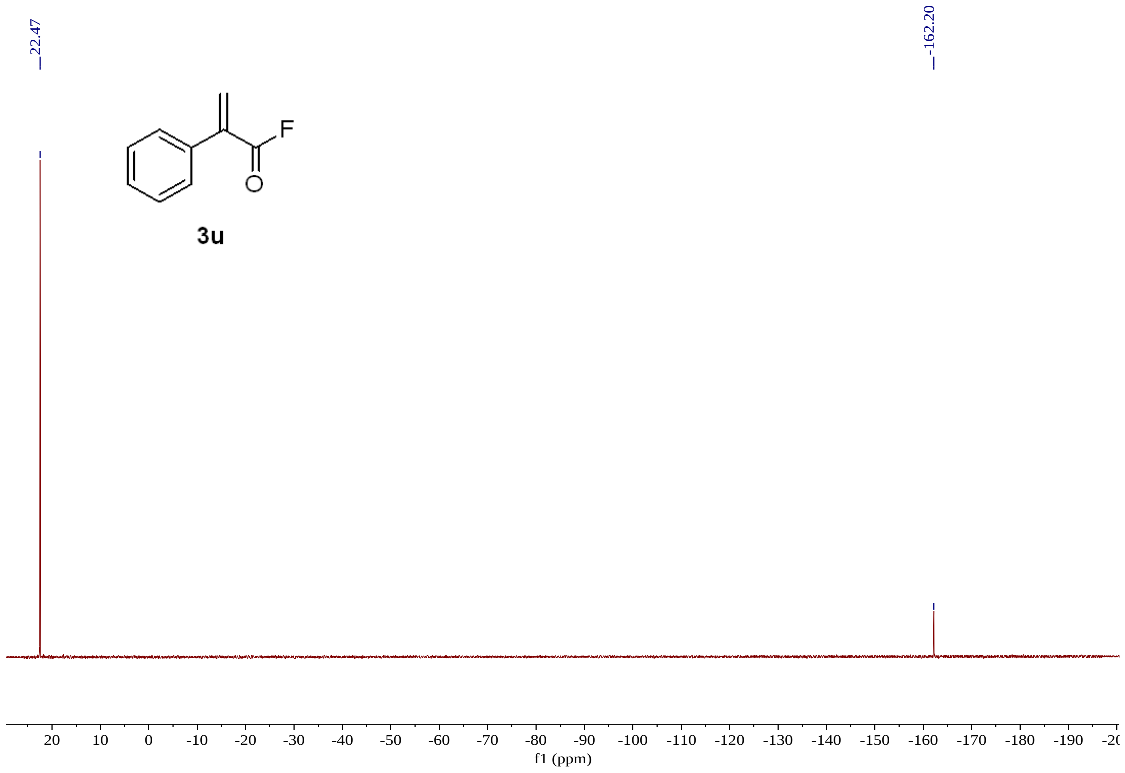


**Supplementary Figure 87.** $^{19}$F NMR Spectra of **3u.**

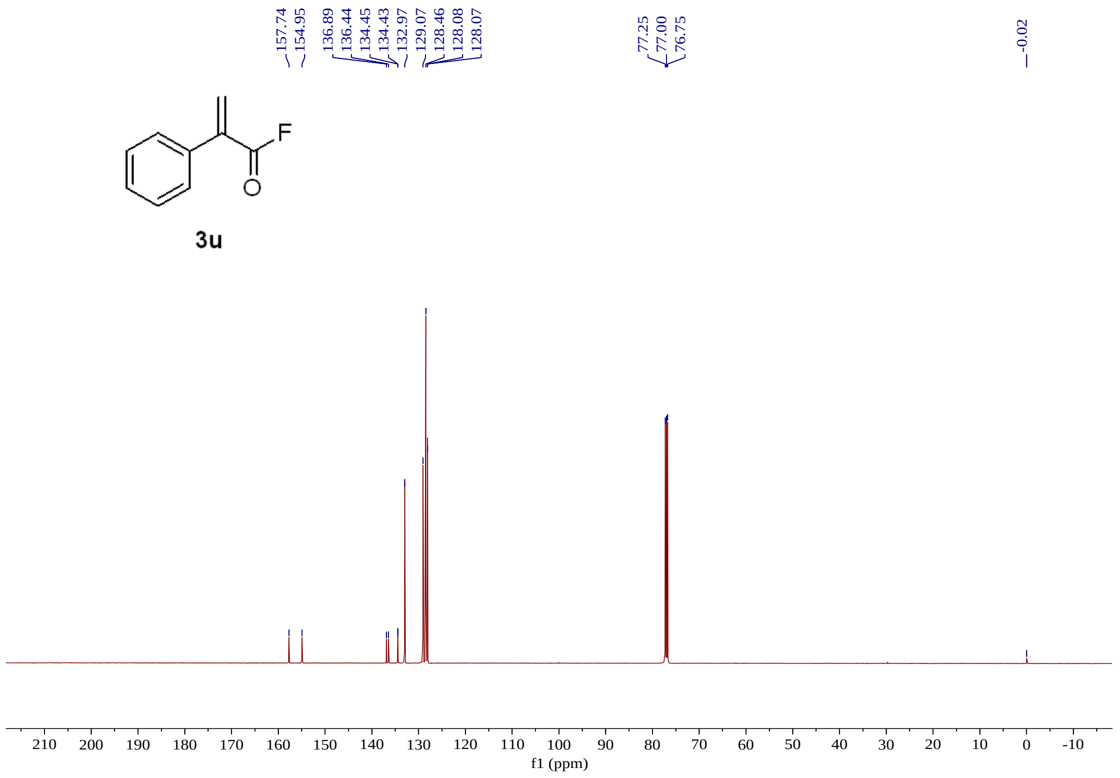


**Supplementary Figure 88.** $^{13}$C NMR Spectra of **3u.**

***(E)*-Undec-2-enoyl fluoride (3v)**

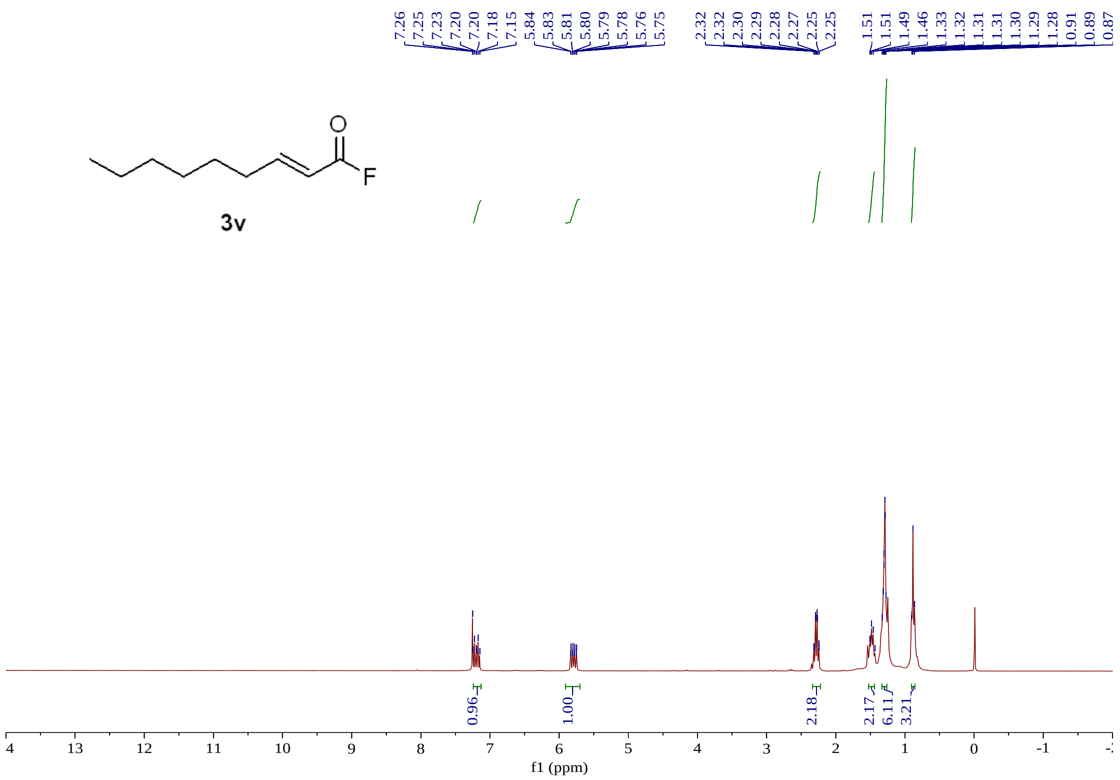


**Supplementary Figure 89.** $^{1}$H NMR Spectra of **3v.**

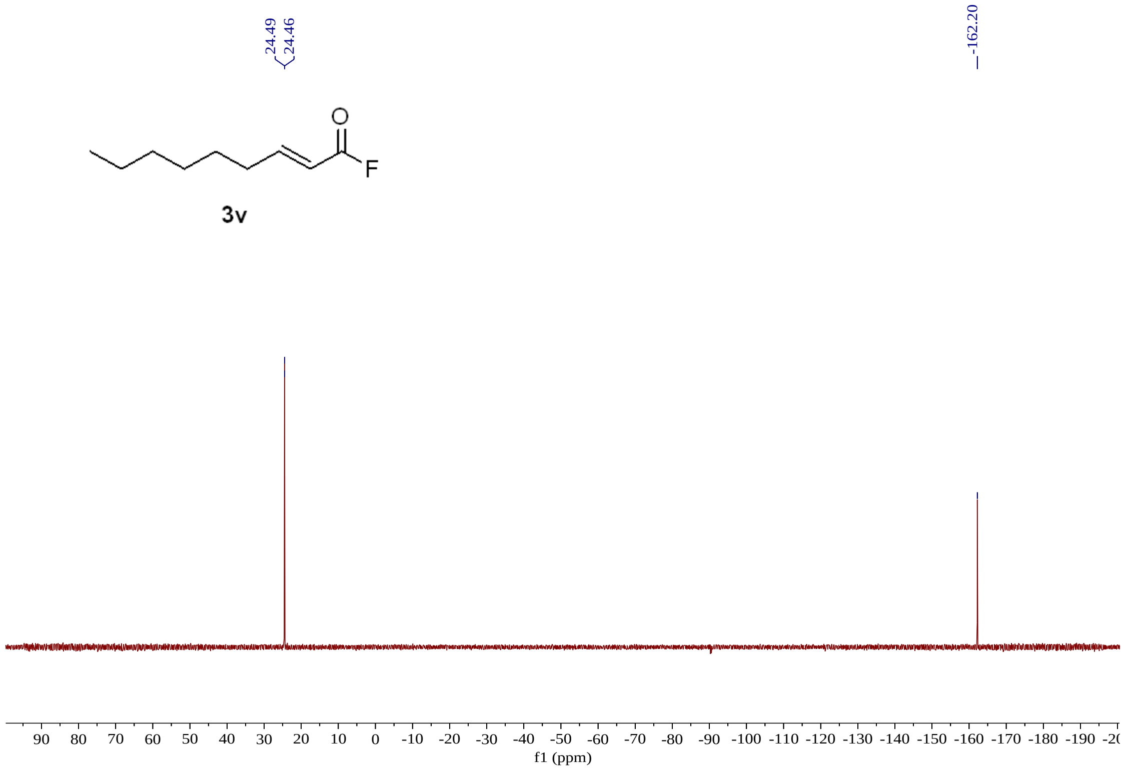


**Supplementary Figure 90.** $^{19}$F NMR Spectra of **3v.**

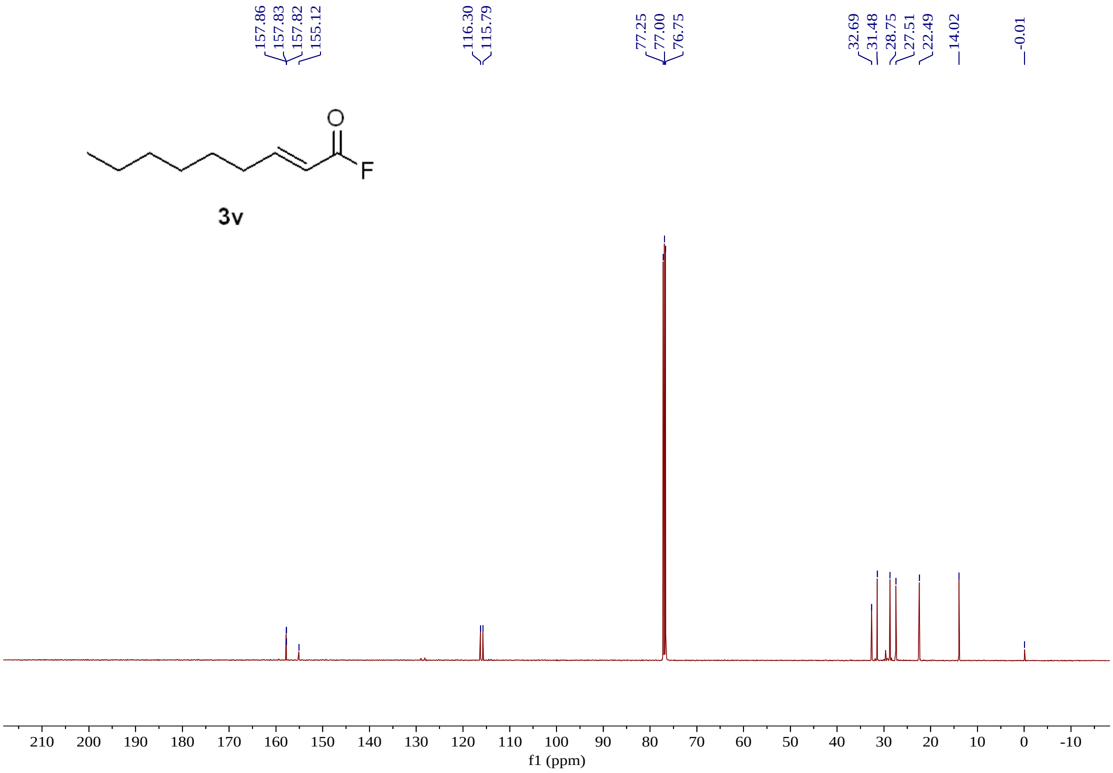


**Supplementary Figure 91.** $^{13}$C NMR Spectra of **3v.**

**(*1S*,*2R*,*5S*)-2-Isopropyl-5-methylcyclohexyl 4-(fluorocarbonyl)benzoate (3w)**

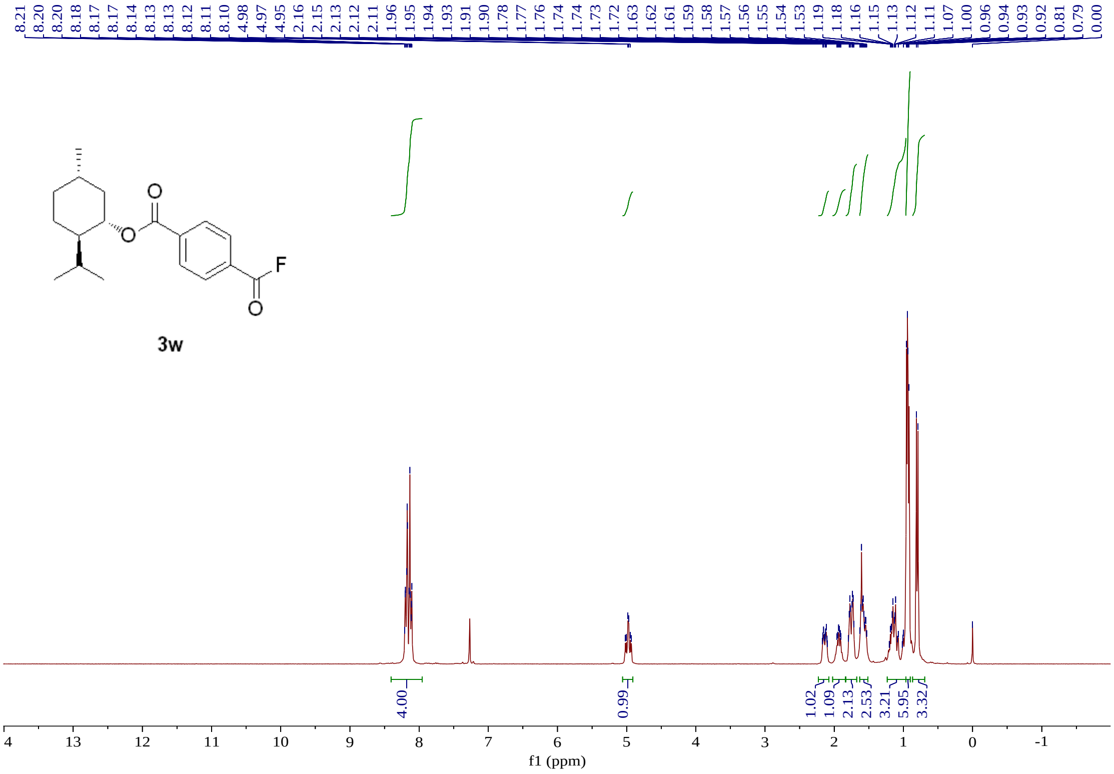


**Supplementary Figure 92.** $^{1}$H NMR Spectra of **3w.**

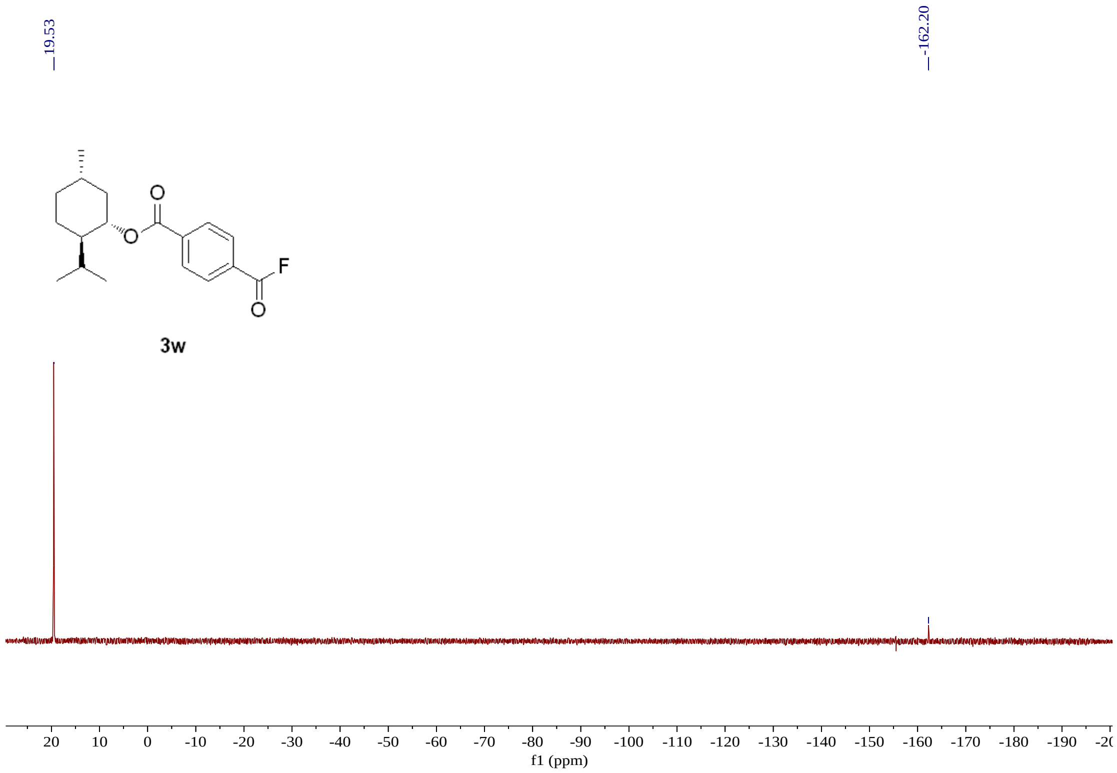


**Supplementary Figure 93.** $^{19}$F NMR Spectra of **3w.**

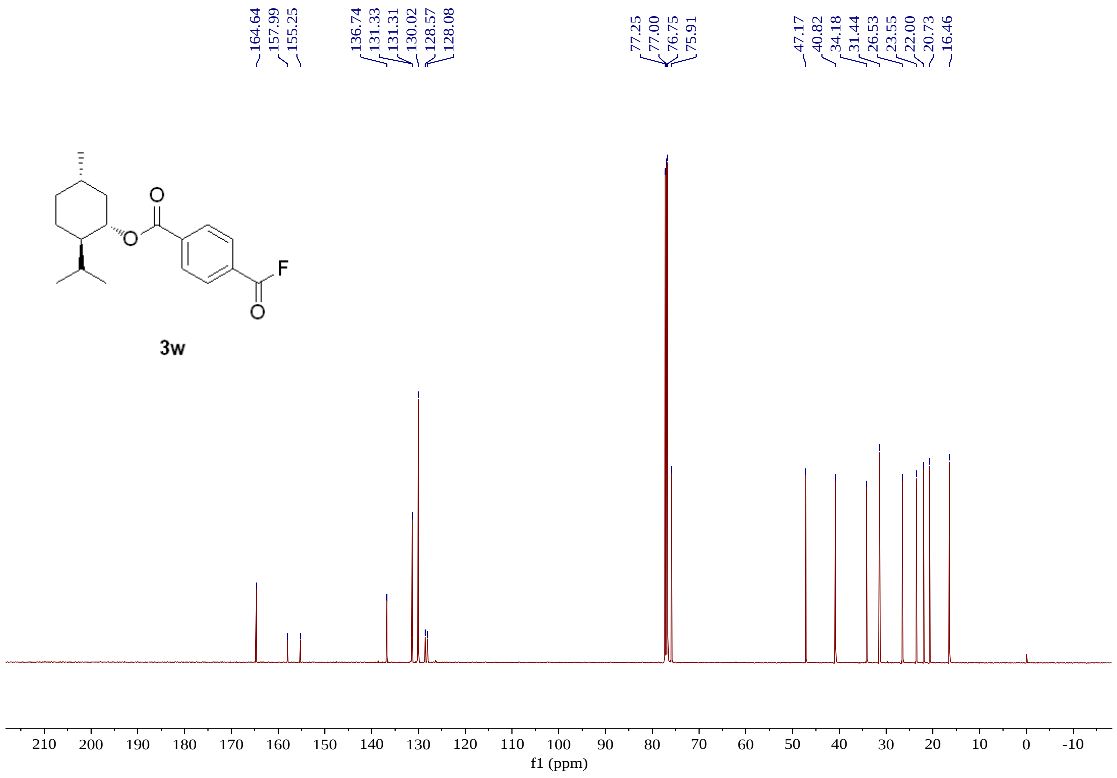


**Supplementary Figure 94.** $^{13}$C NMR Spectra of **3w.**

**Isopropyl 2-(4-(4-(fluorocarbonyl)benzoyl)phenoxy)-2-methylpropanoate (3x)**

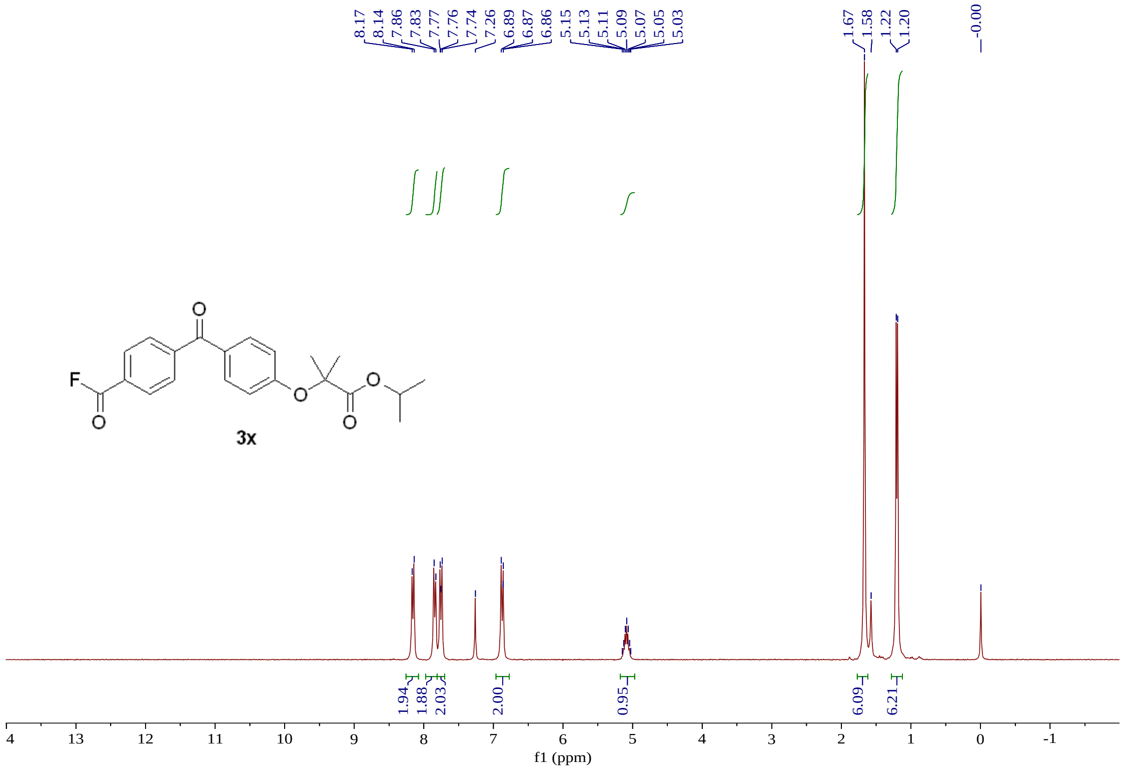


**Supplementary Figure 95.** $^{1}H$ NMR Spectra of **3x.**

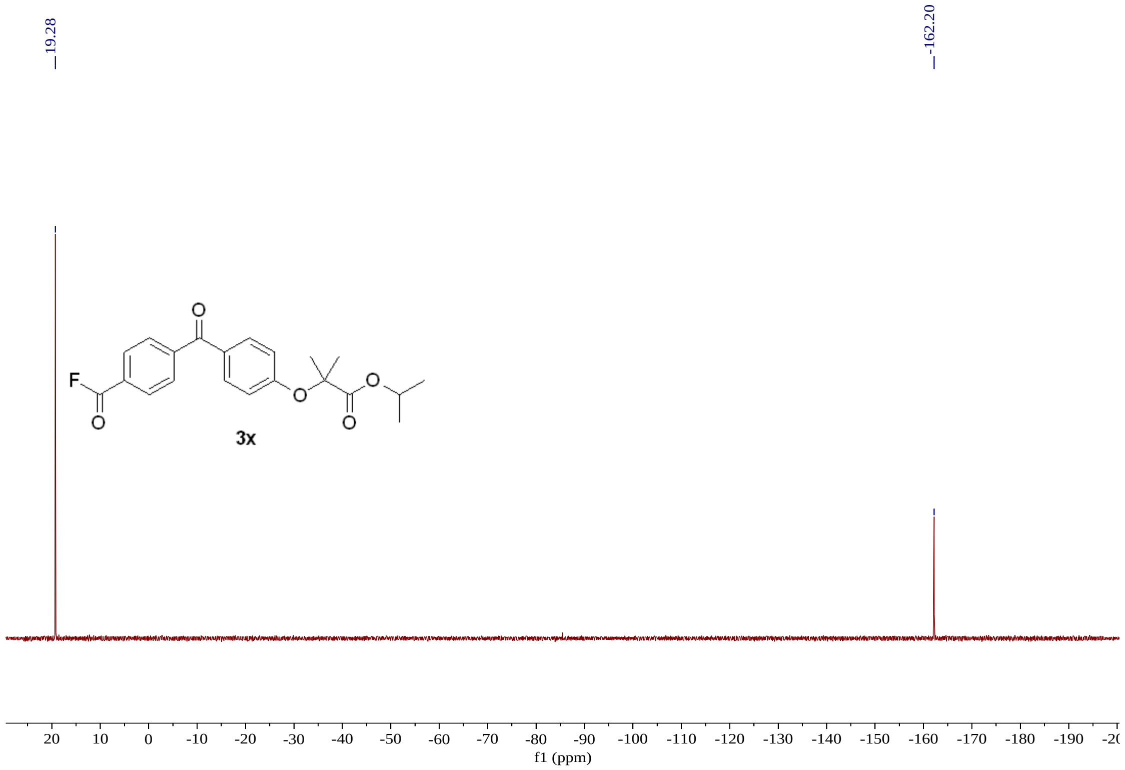


**Supplementary Figure 96.** $^{19}F$ NMR Spectra of **3x.**

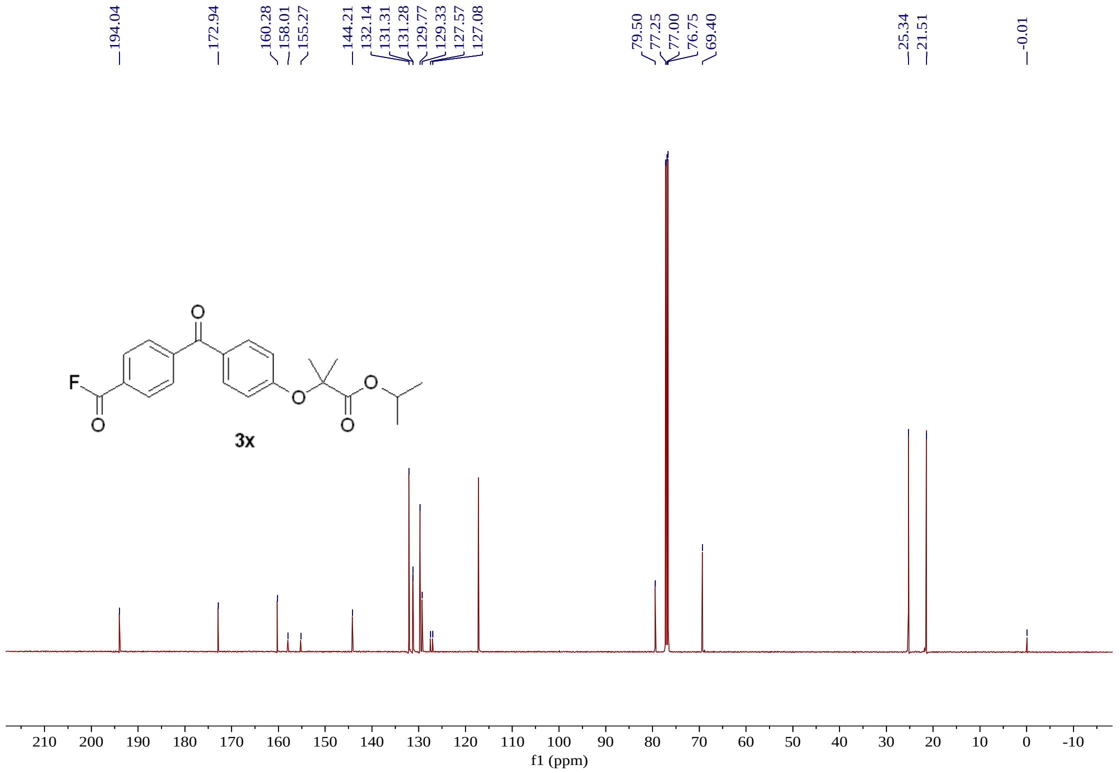

**Supplementary Figure 97.** $^{13}$C NMR Spectra of **3x.**

**(8*R*,9*S*,13*S*,14*S*)-13-Methyl-17-oxo-7,8,9,11,12,13,14,15,16,17-decahydro-6*H*-cyclopenta[*a*]phenanthrene-3-carbonyl fluoride (3y)**

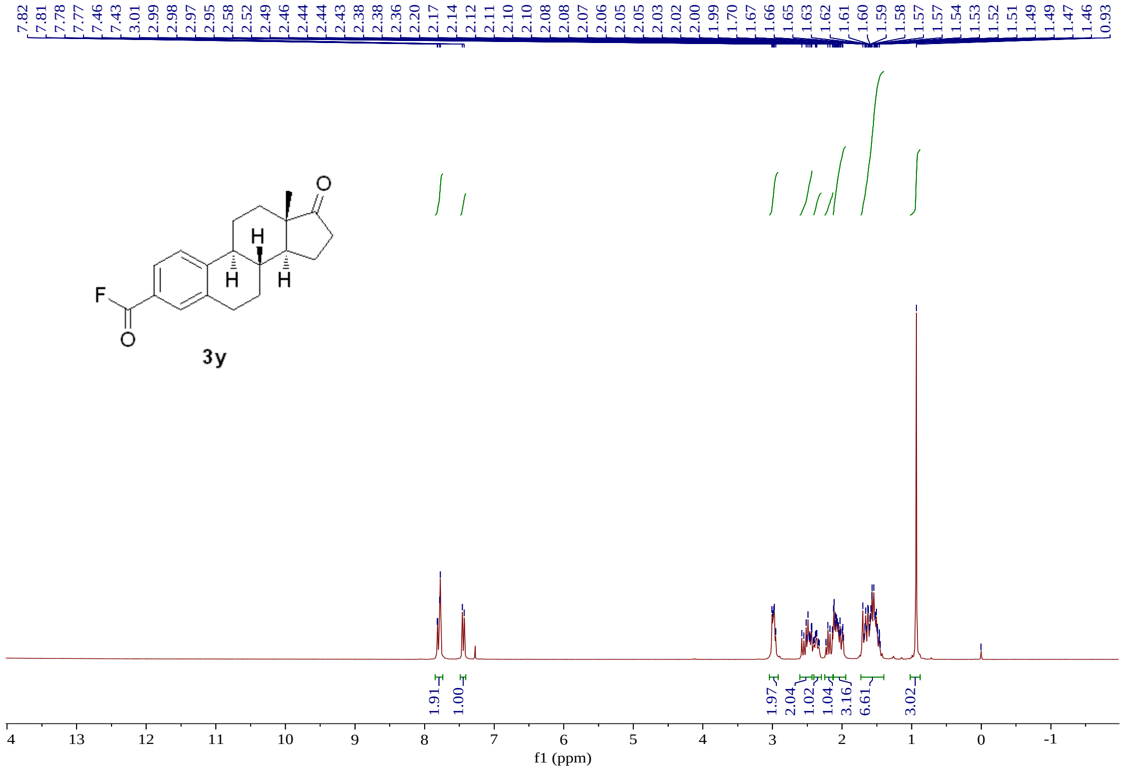

**Supplementary Figure 98.** $^{1}$H NMR Spectra of **3y.**

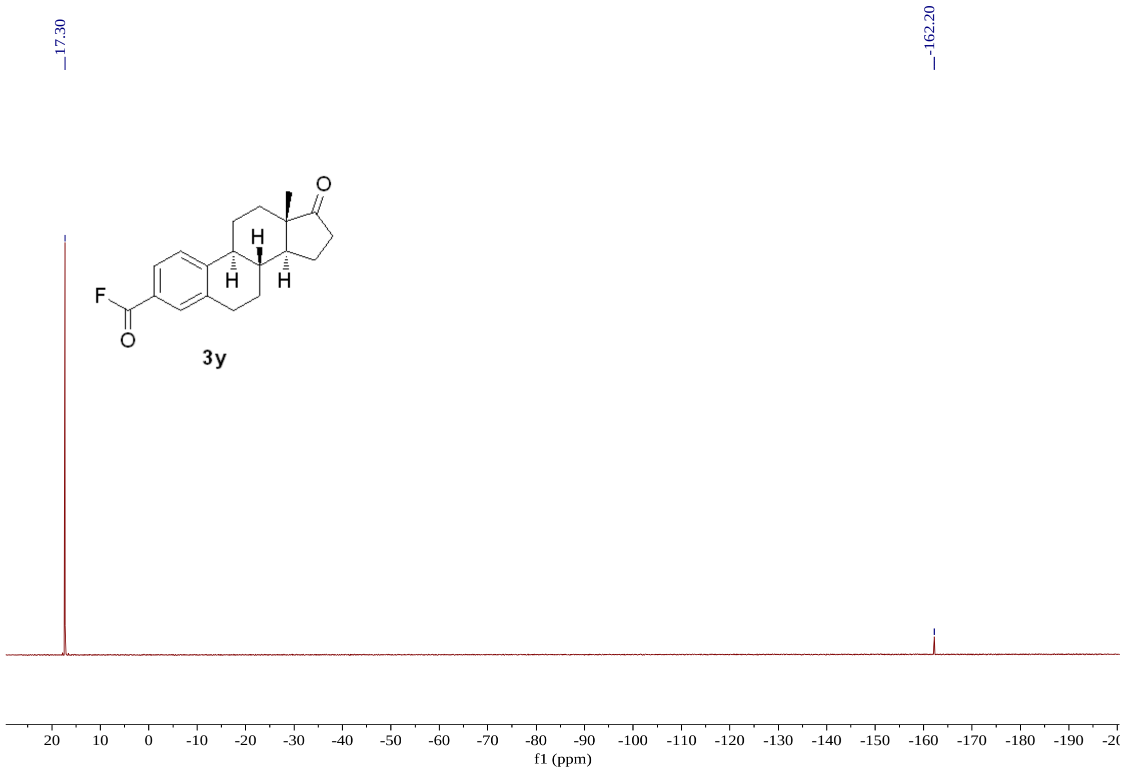


**Supplementary Figure 99.** $^{19}$F NMR Spectra of **3y.**

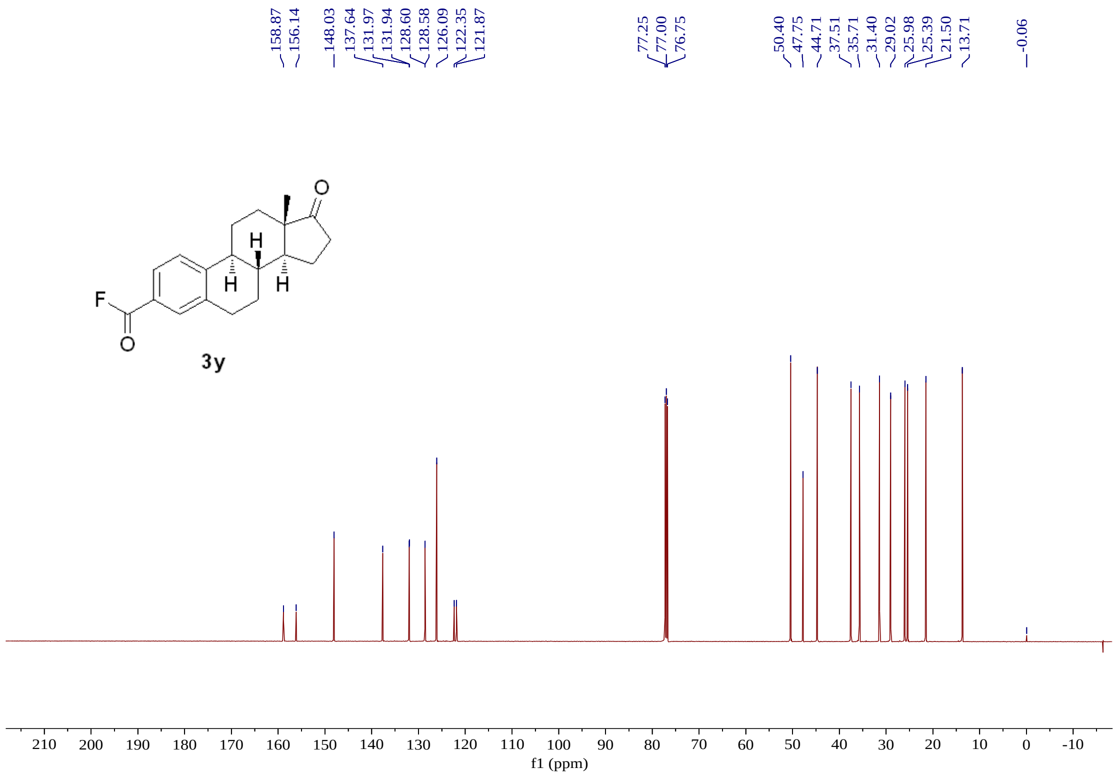


**Supplementary Figure 100.** $^{13}$C NMR Spectra of **3y.**

**(8*R*,9*S*,13*S*,14*S*)-13-Methyl-17-oxo-7,8,9,11,12,13,14,15,16,17-decahydro-6*H*-cyclopenta[*a*]phenanthren-3-yl 4-(fluorocarbonyl)benzoate (3z)**

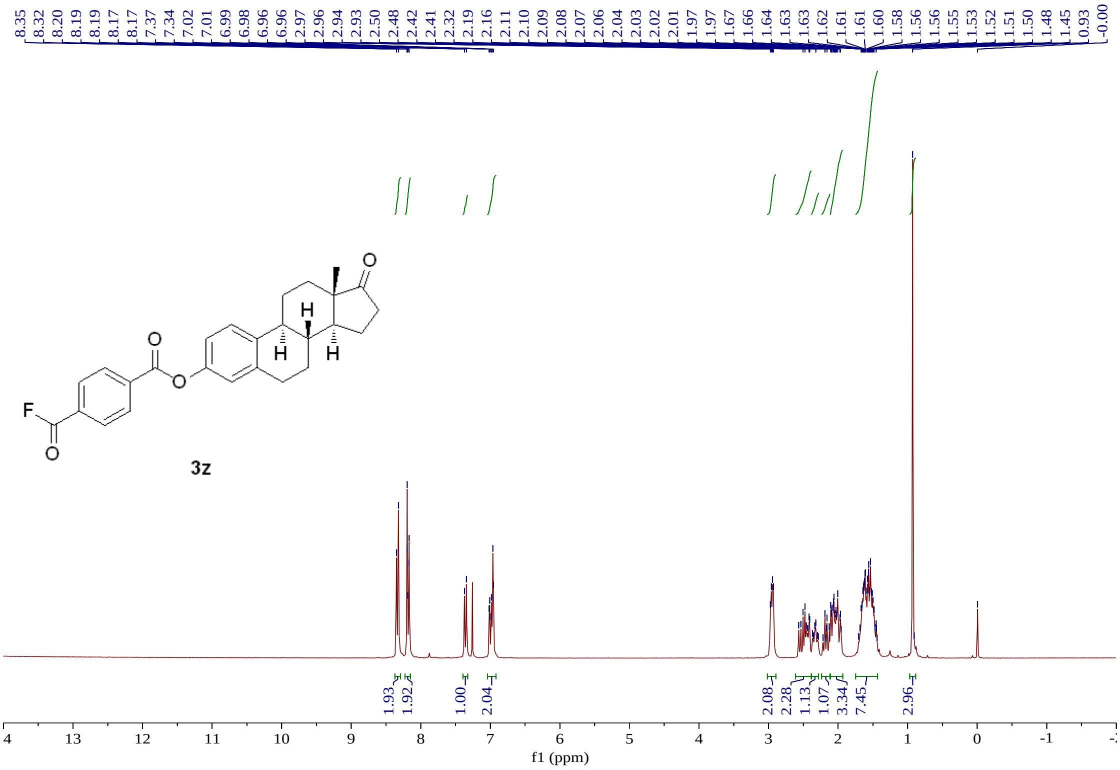

**Supplementary Figure 101.** $^{1}$H NMR Spectra of **3z.**

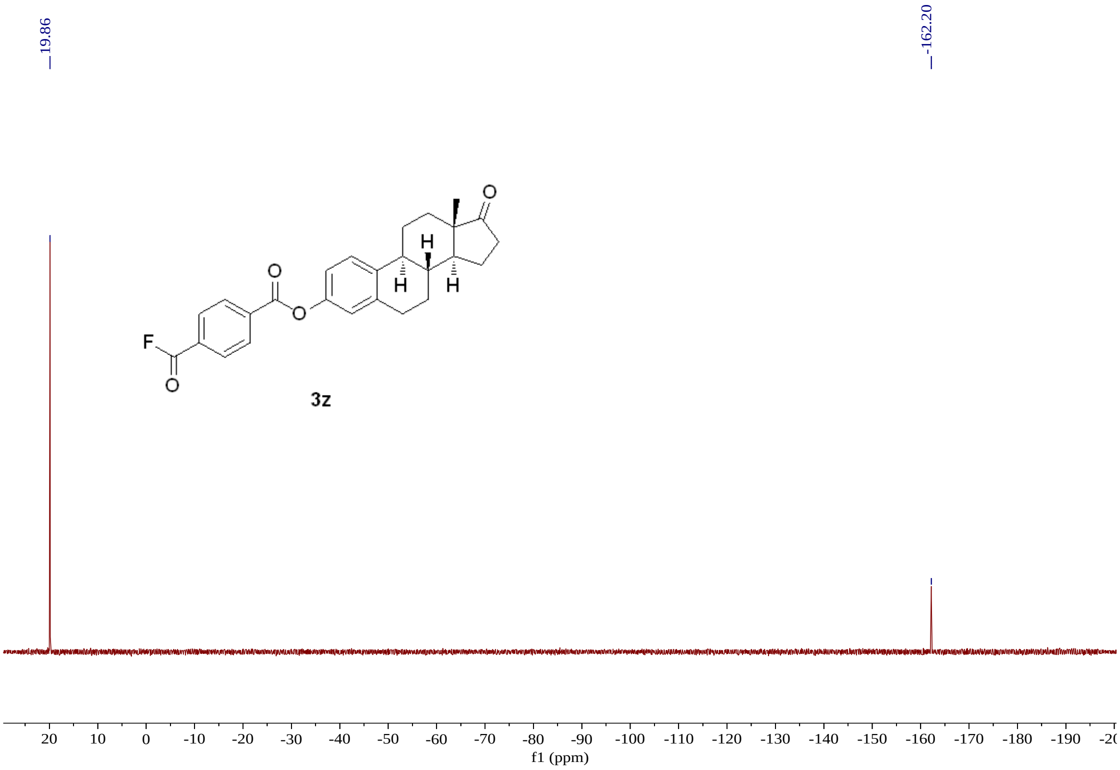

**Supplementary Figure 102.** $^{19}$F NMR Spectra of **3z.**

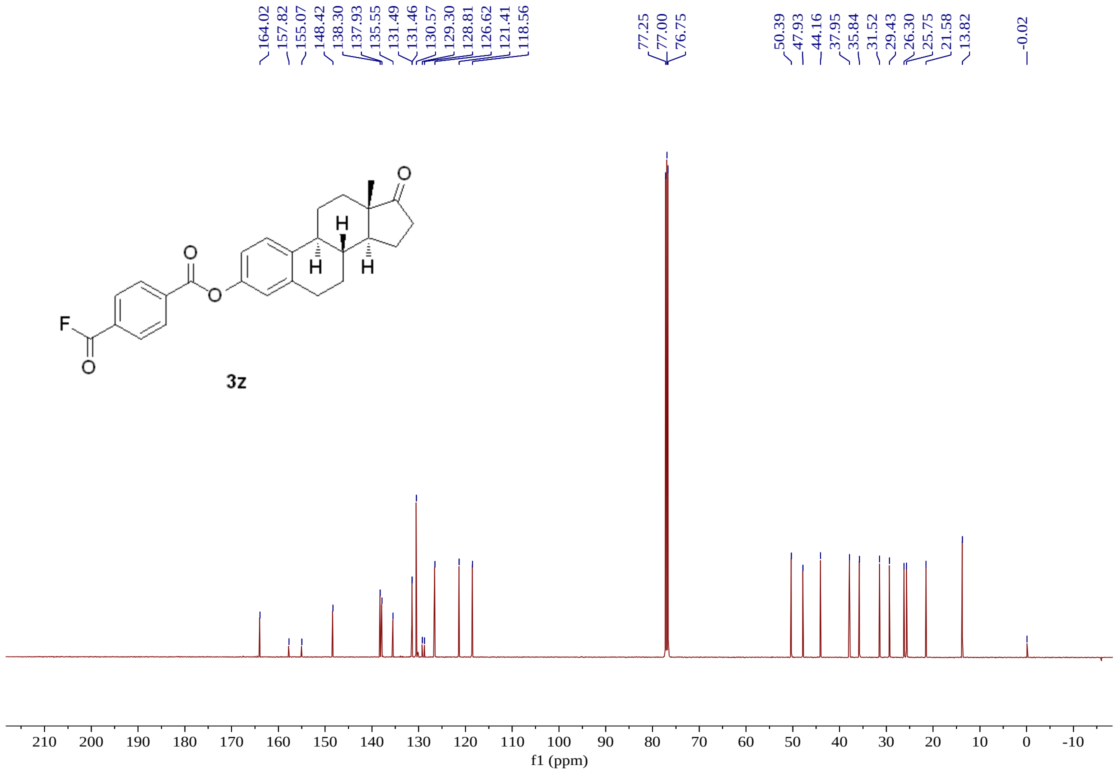


**Supplementary Figure 103.** $^{13}$C NMR Spectra of **3z.**

**4-(Fluorocarbonyl)benzyl 2-(11-oxo-6,11-dihydrodibenzo[*b*,*e*]oxepin-2-yl)acetate (3za)**

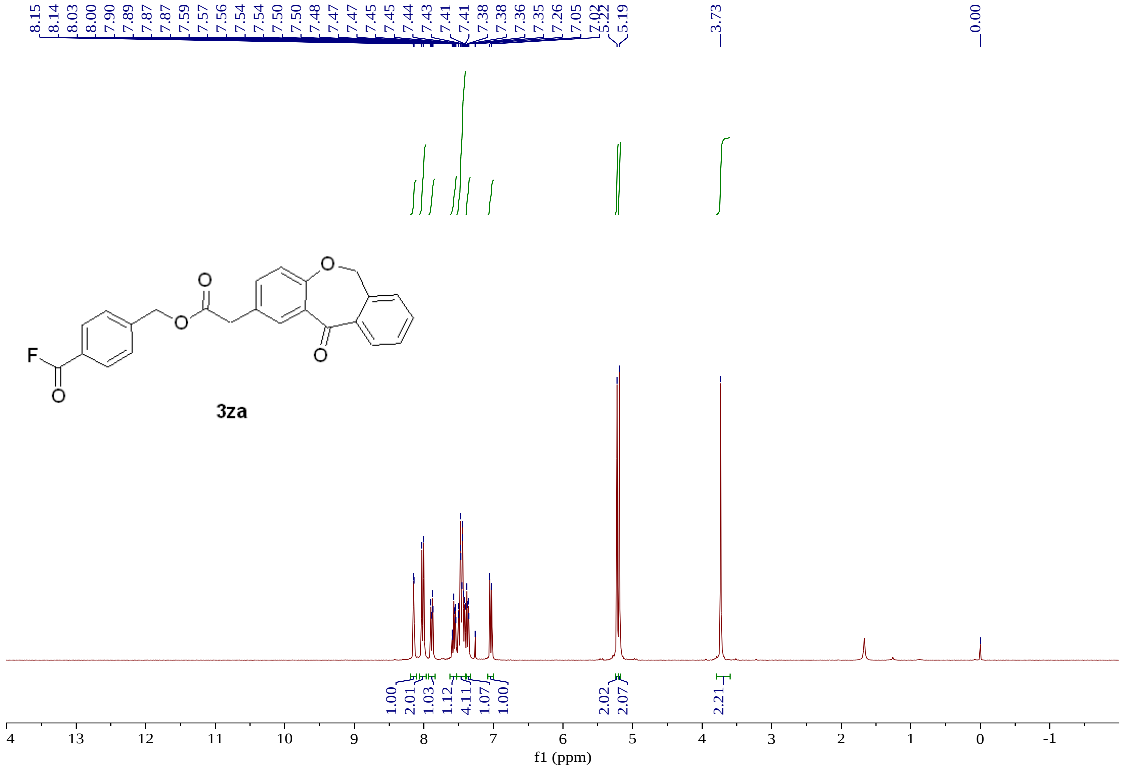


**Supplementary Figure 104.** $^{1}$H NMR Spectra of **3za.**

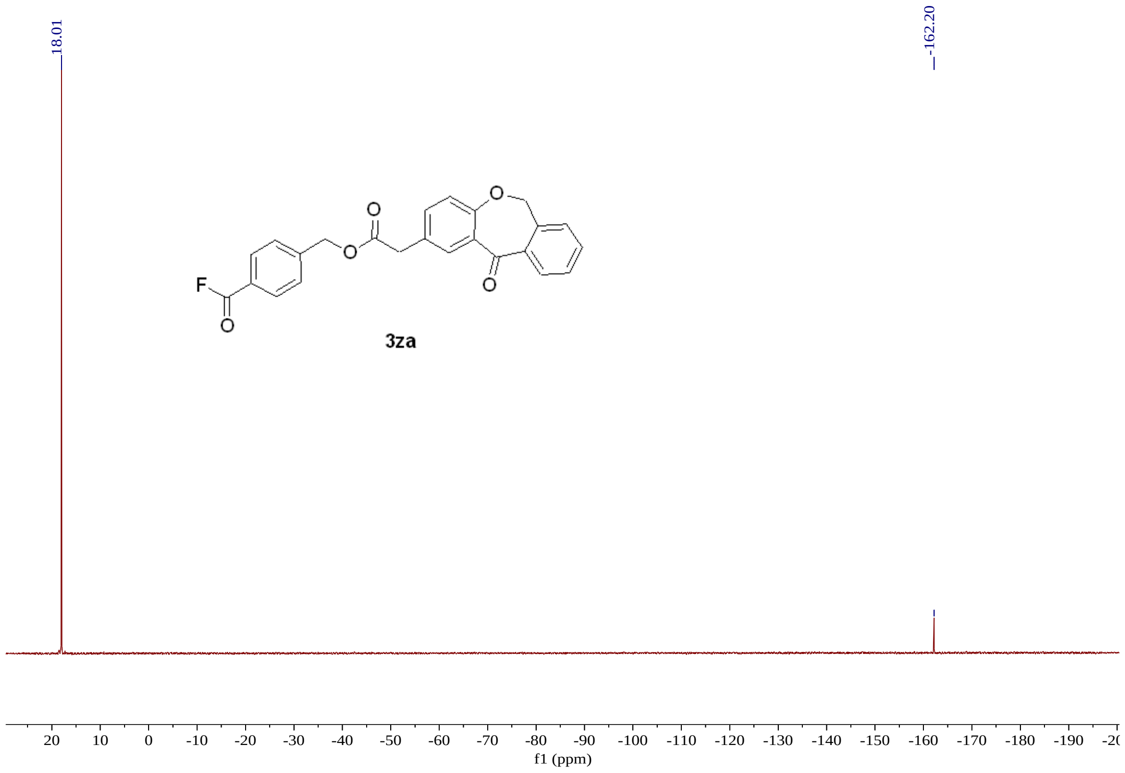


**Supplementary Figure 105.** $^{19}$F NMR Spectra of **3za.**

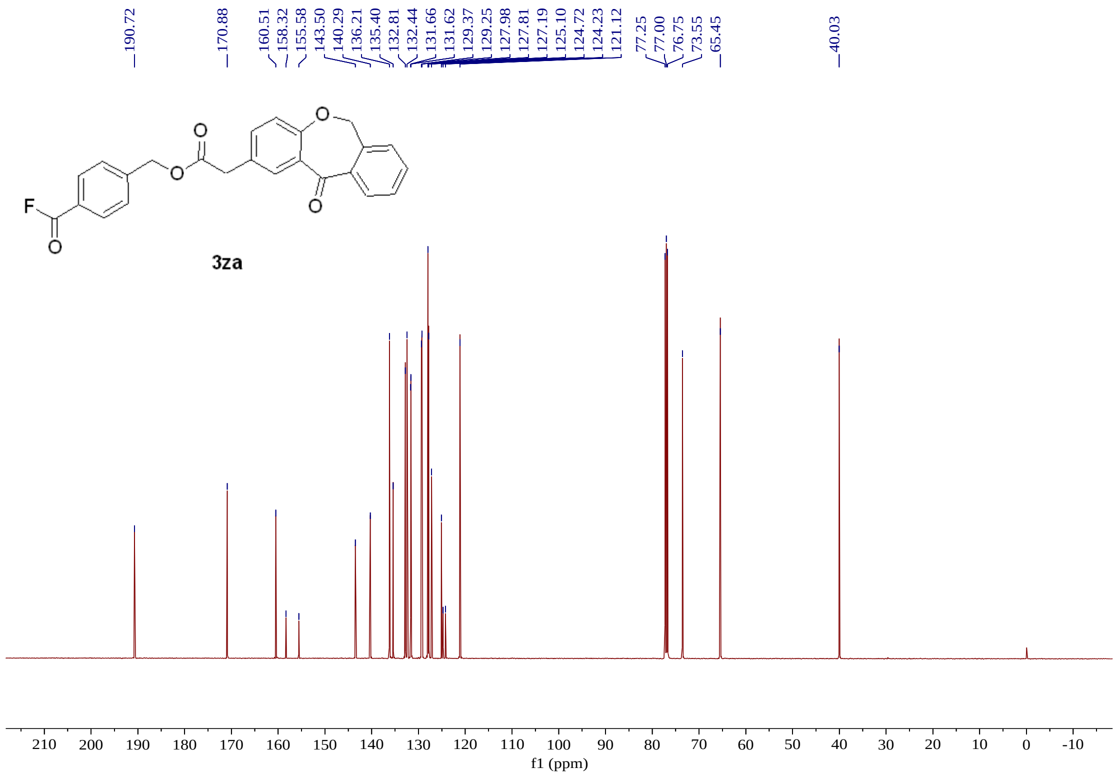


**Supplementary Figure 106.** $^{13}$C NMR Spectra of **3za.**

**(All-rac)-4-(((2,5,7,8-Tetramethyl-2-(4,8,12-trimethyltridecyl)chroman-6-yl)oxy)methyl)benzoyl fluoride (3zb)**

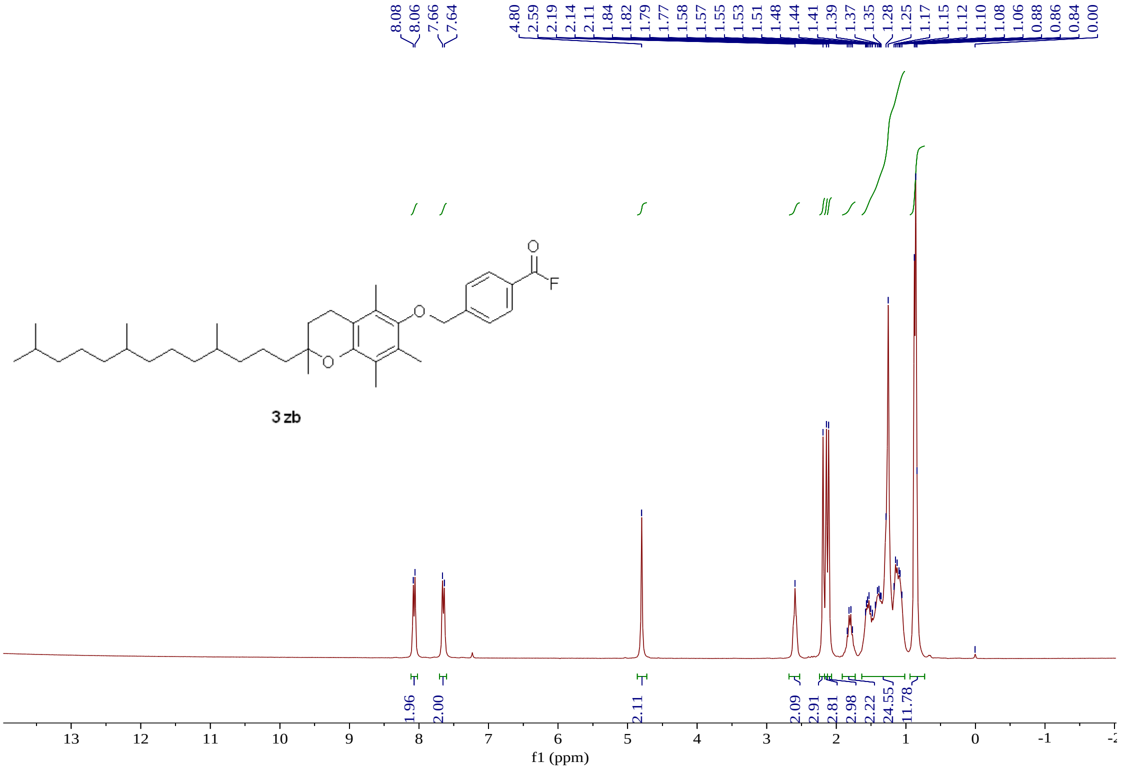


**Supplementary Figure 107.** $^{1}$H NMR Spectra of **3zb.**

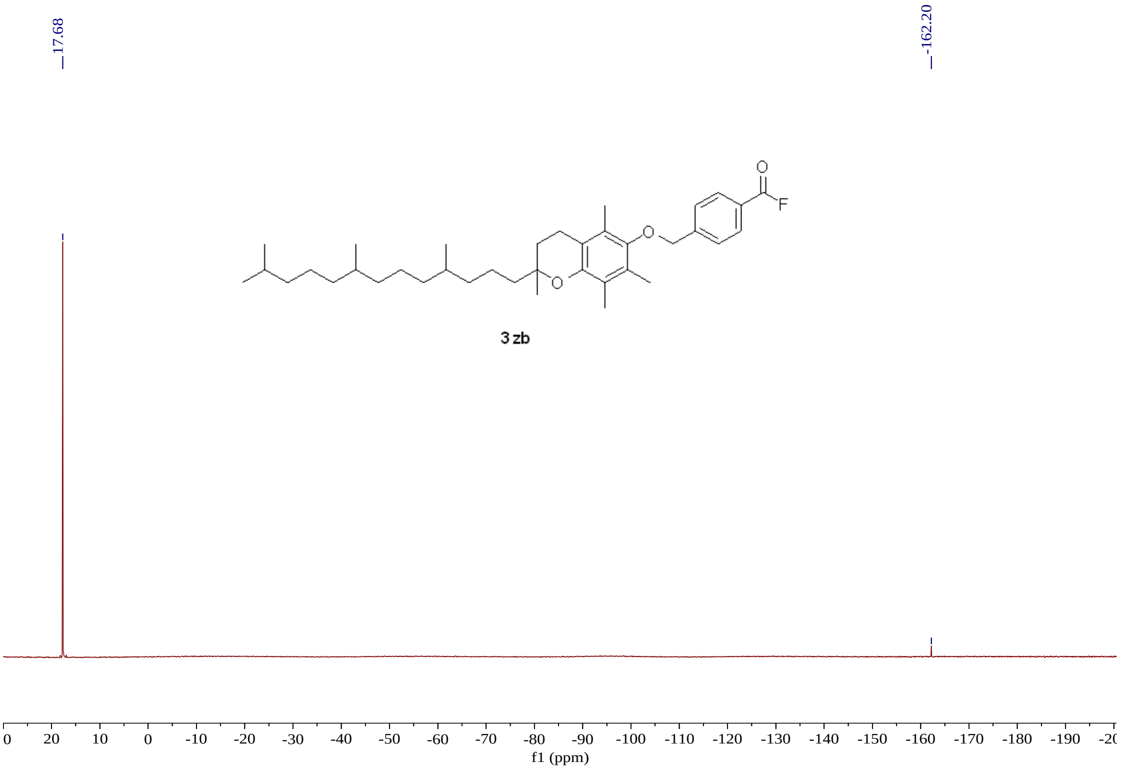


**Supplementary Figure 108.** $^{19}$F NMR Spectra of **3zb.**

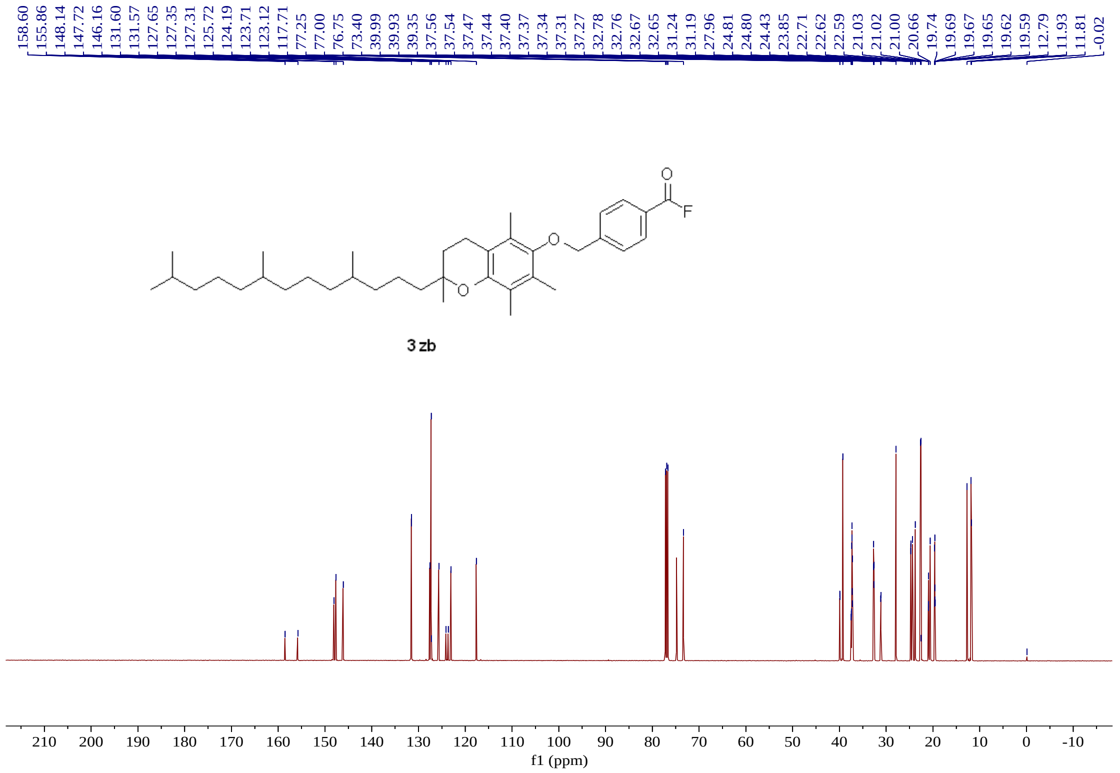


**Supplementary Figure 109.** $^{13}$C NMR Spectra of **3zb.**

**(8*R*,9*S*,10*R*,13*S*,14*S*,17*R*)-10,13-Dimethyl-3-oxo-2,3,6,7,8,9,10,11,12,13,14,15,16,17-tetradecahydro-1*H*-cyclopenta[*a*]phenanthren-17-yl 4-(fluorocarbonyl)benzoate (3zc)**

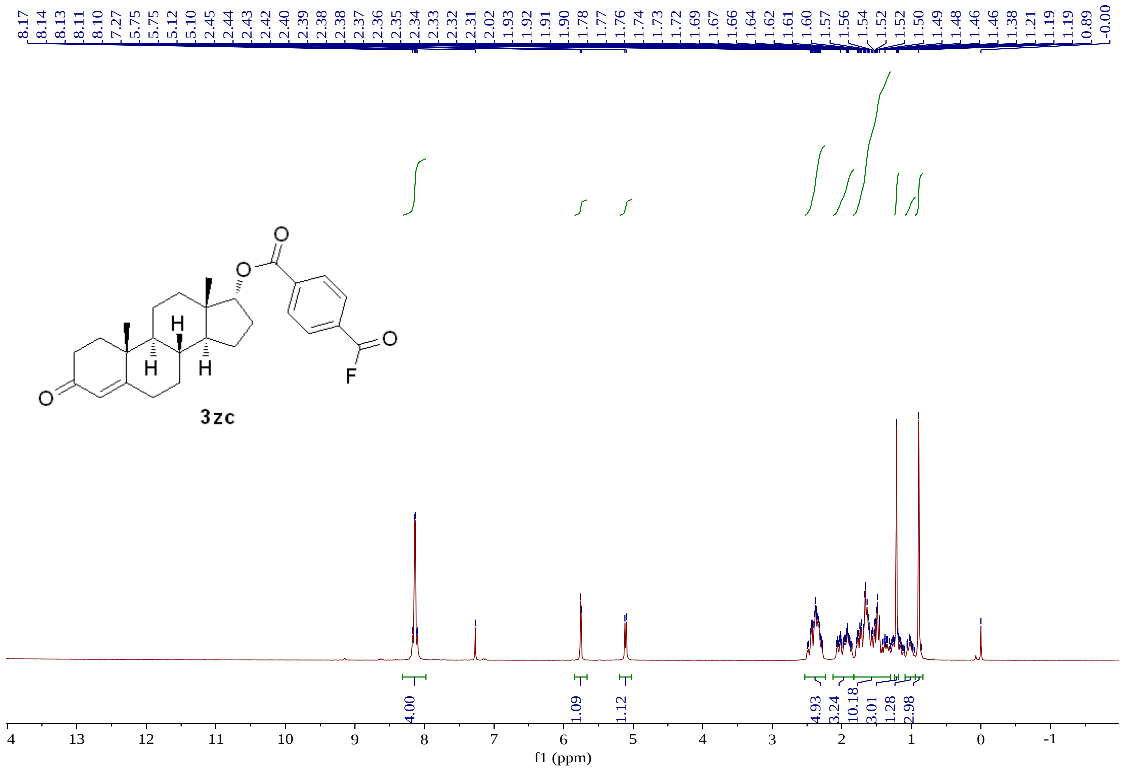


**Supplementary Figure 110.** $^{1}$H NMR Spectra of **3zc.**

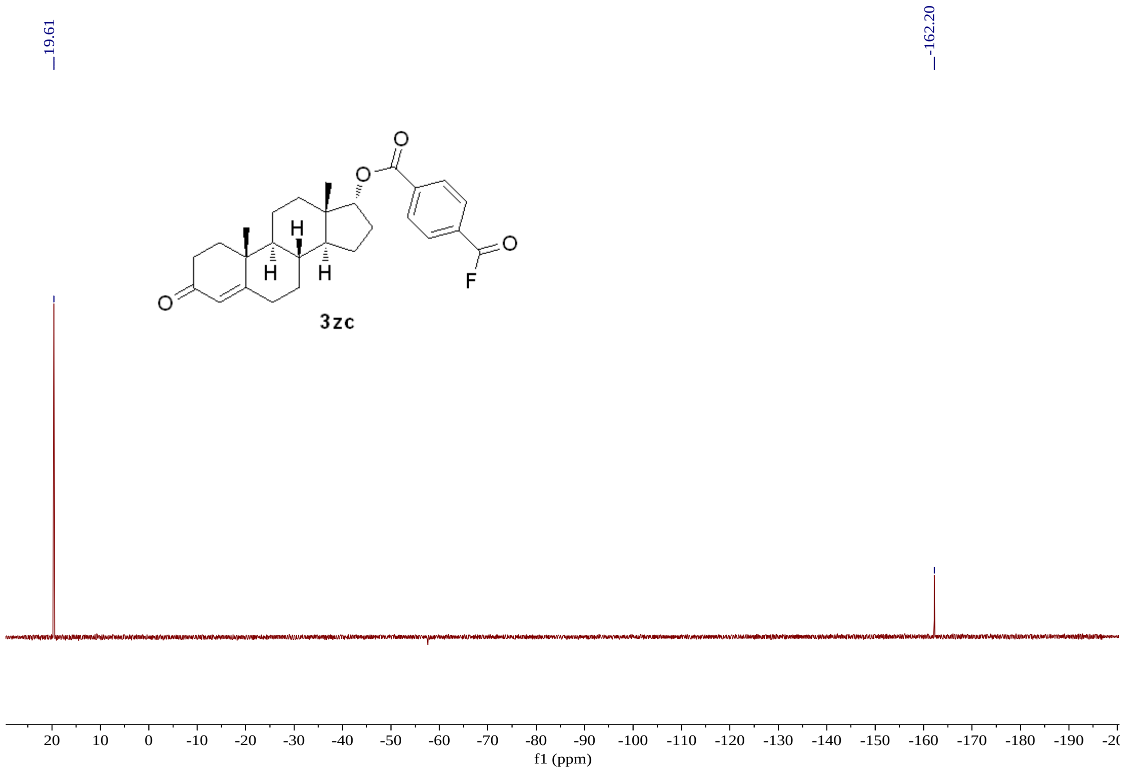


**Supplementary Figure 111.** $^{19}$F NMR Spectra of **3zc.**

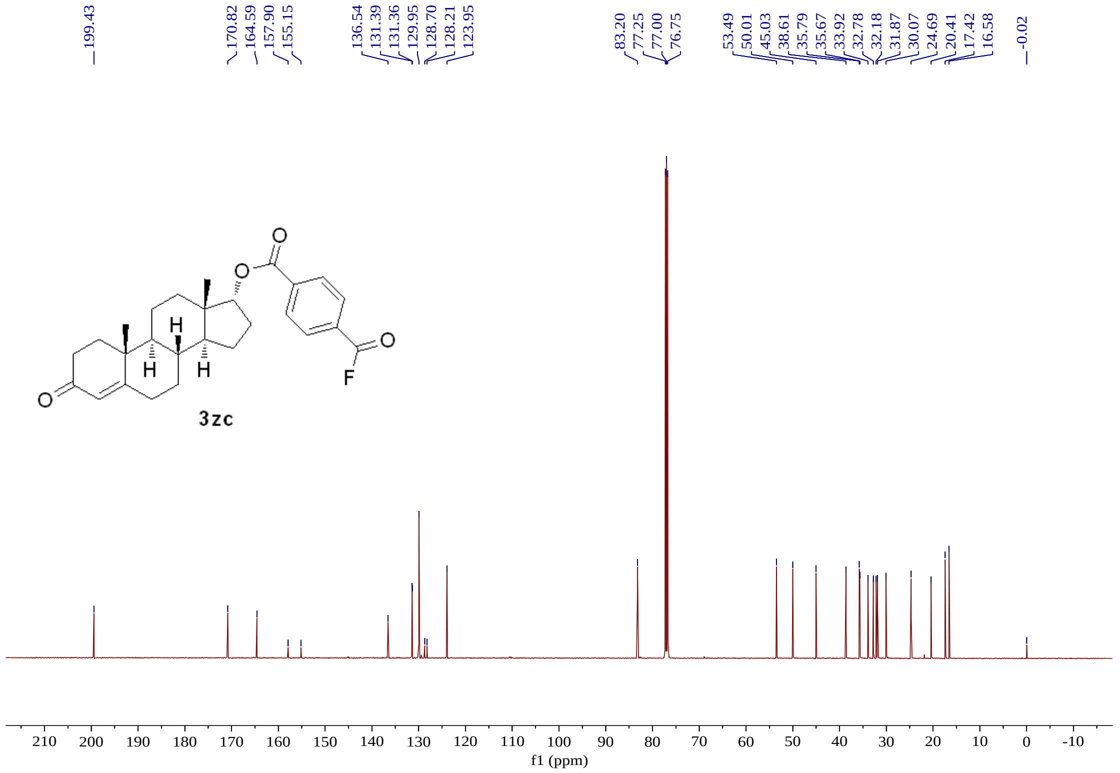


**Supplementary Figure 112.** $^{13}$C NMR Spectra of **3zc.**

**2-Fluoro-5-nitropyridine (4).**

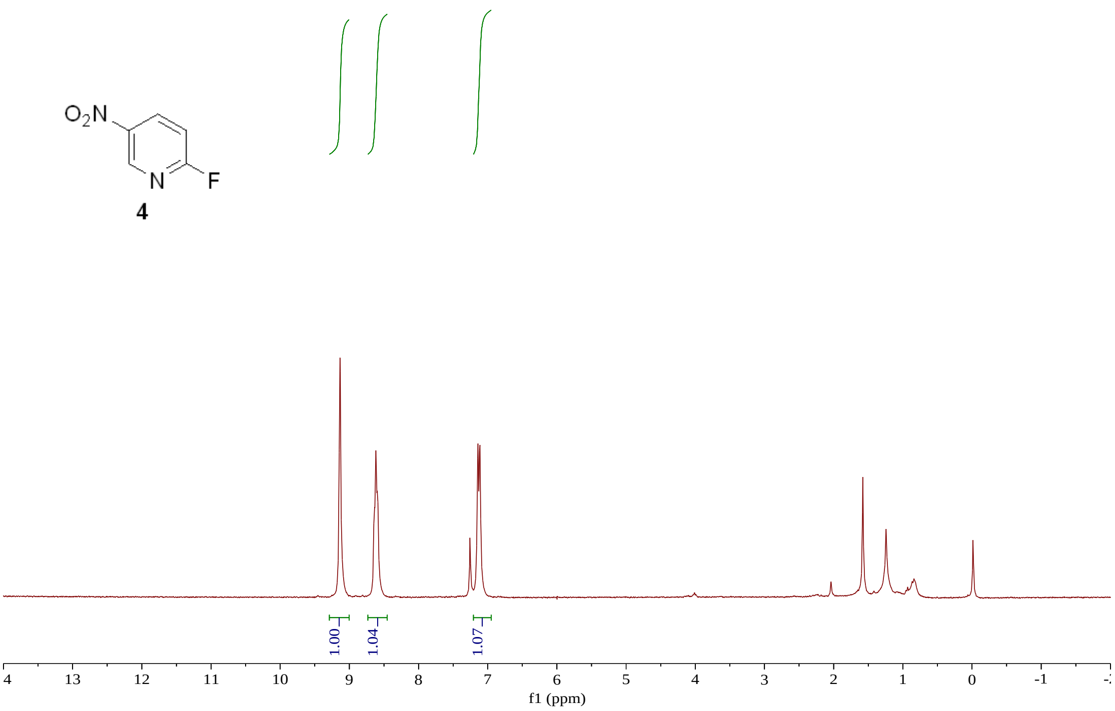


**Supplementary Figure 113.** $^1$H NMR Spectra of **4.**

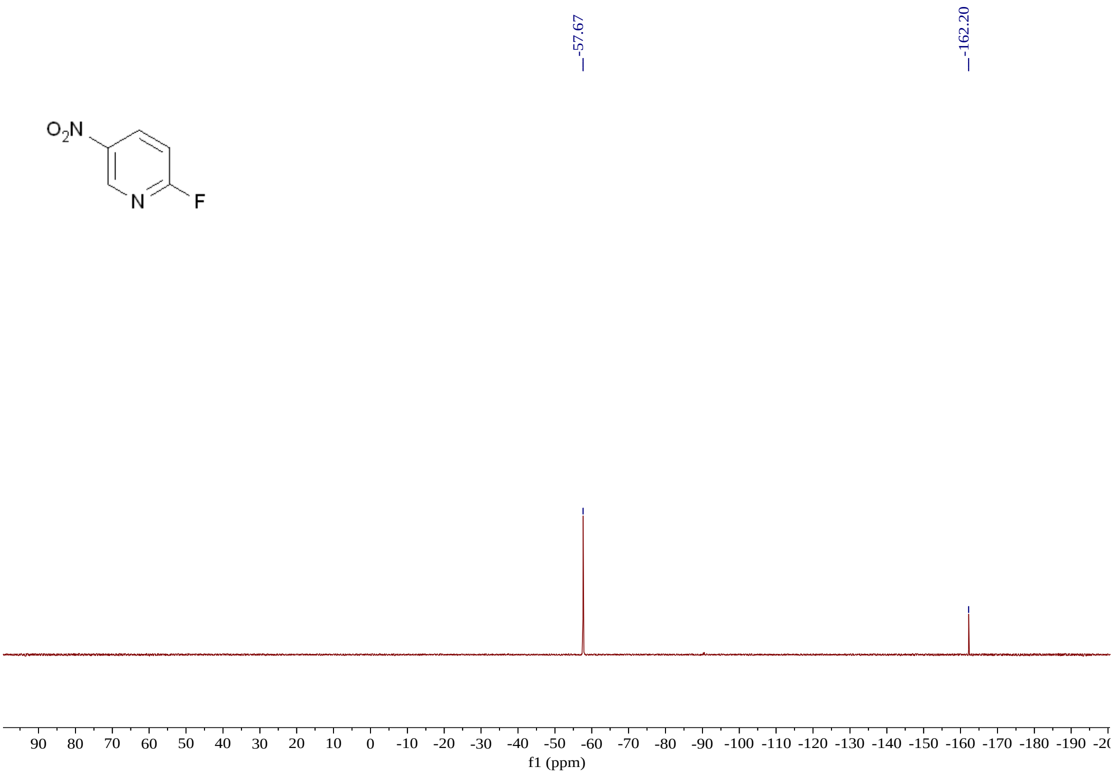


**Supplementary Figure 114.** $^{19}$F NMR Spectra of **4.**

**_N_-phenyl-[1,1'-biphenyl]-4-carboxamide (7a)**

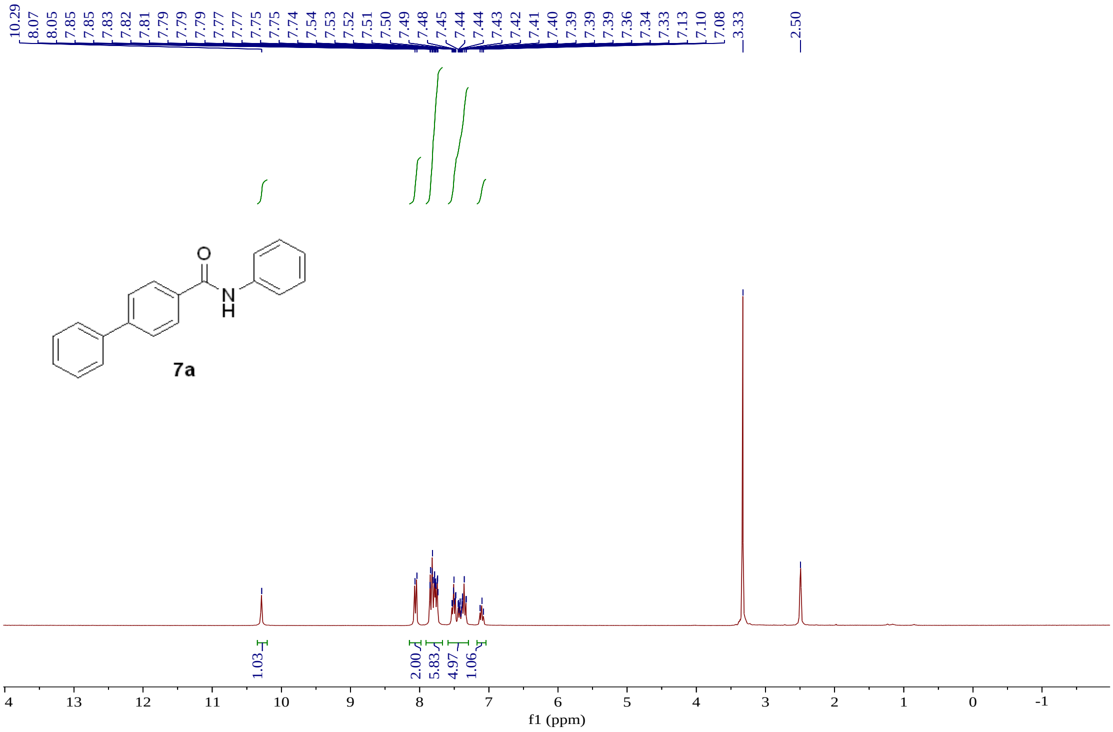


**Supplementary Figure 115.** $^{1}$H NMR Spectra of **7a.**

**4-Nitro-_N_-phenylbenzamide (7zd)**

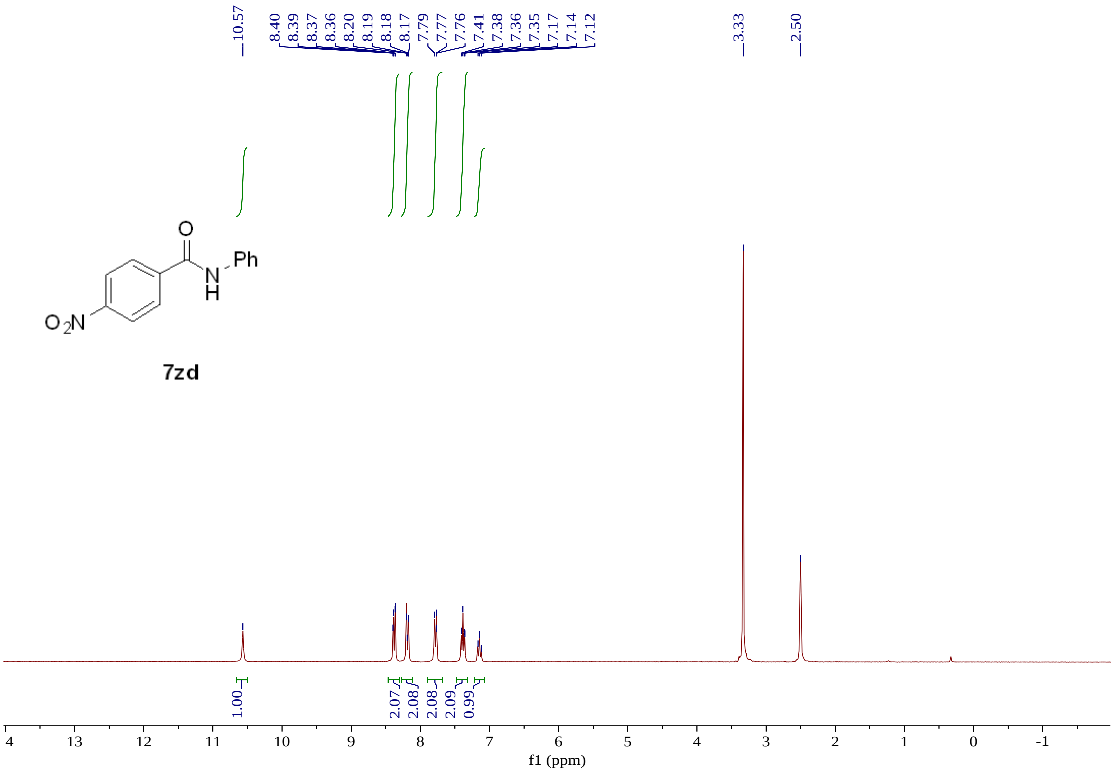


**Supplementary Figure 116.** $^{1}$H NMR Spectra of **7zd.**

**4-Cyano-*N*-phenylbenzamide (7ze)**

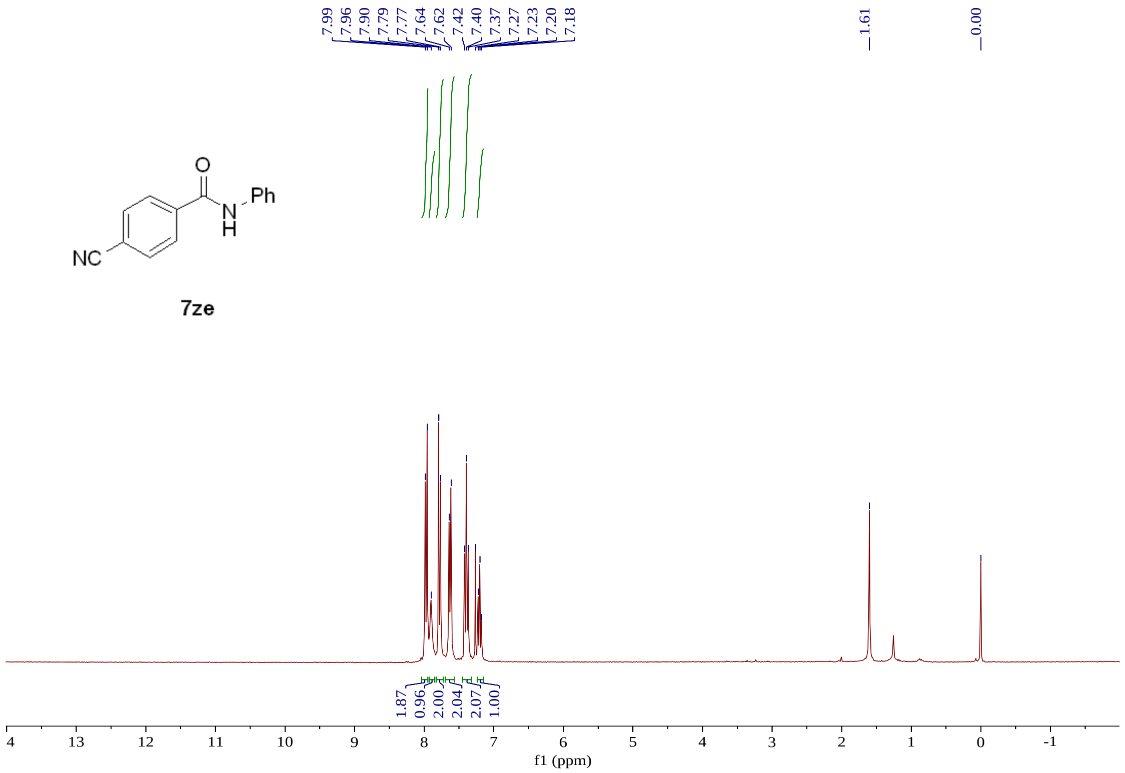


**Supplementary Figure 117.** $^{1}$H NMR Spectra of **7ze.**

***N*-Phenylpicolinamide (7zf)**

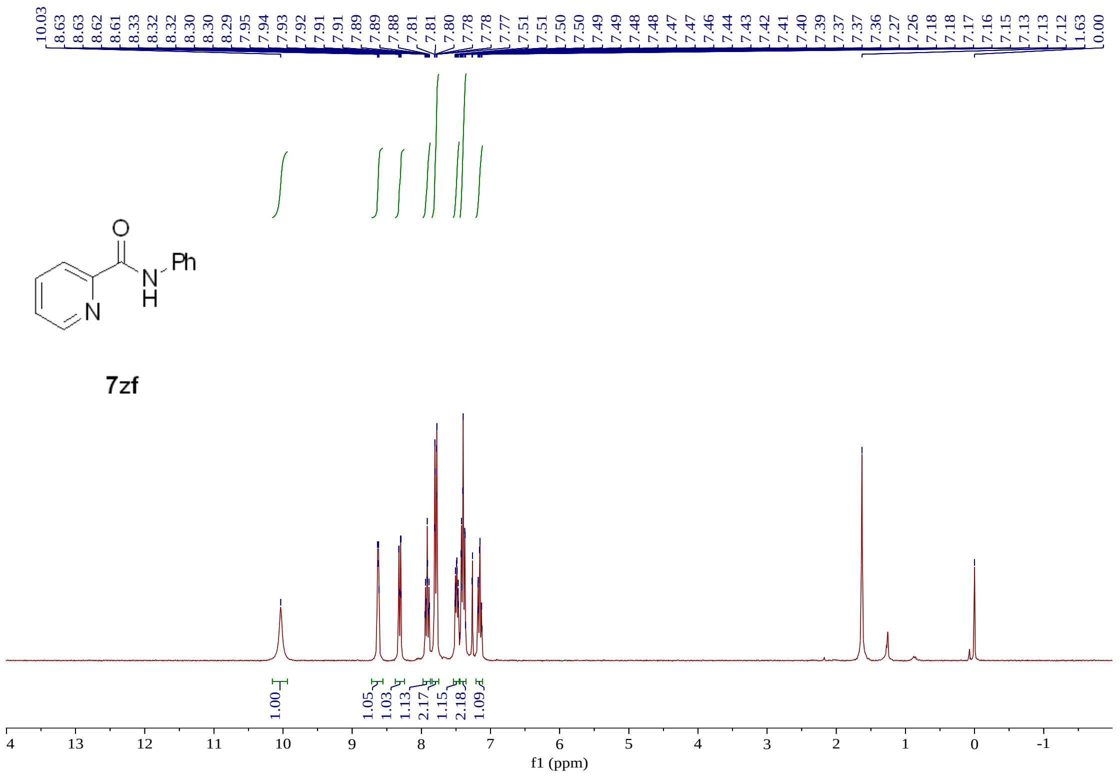


**Supplementary Figure 118.** $^{1}$H NMR Spectra of **7zf.**

***N*-Phenylnicotinamide (7zg)**

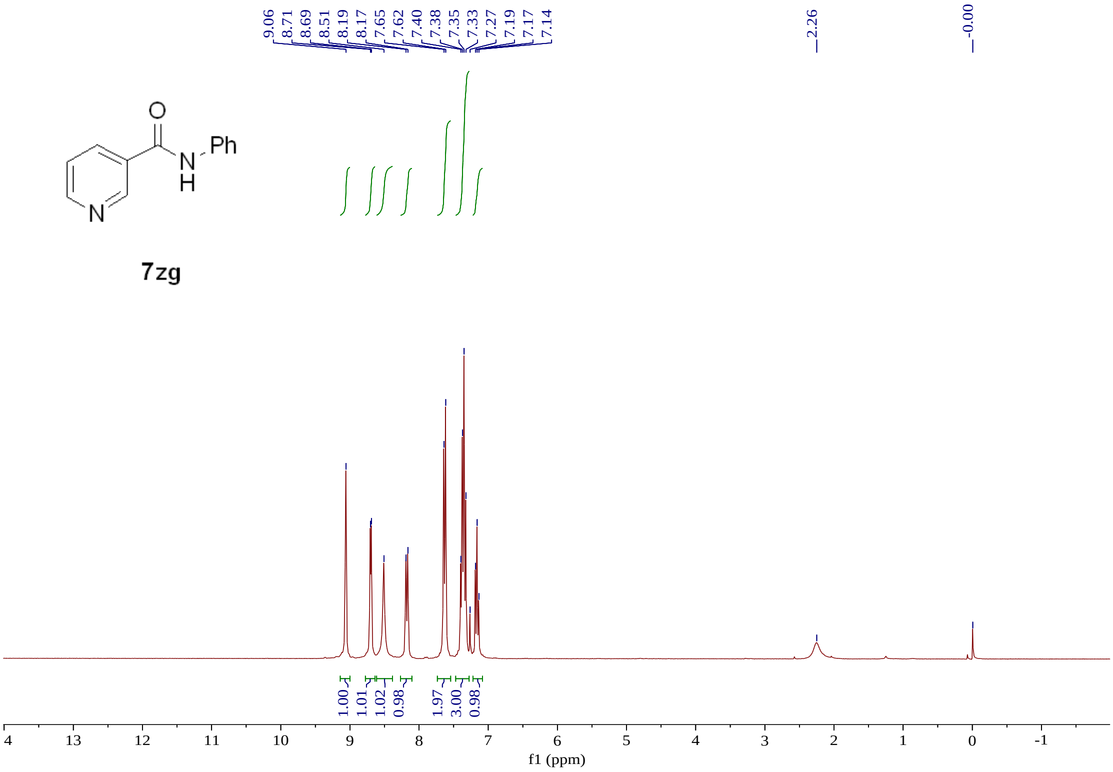


**Supplementary Figure 119.** $^{1}$H NMR Spectra of **7zg.**

***N*-Phenylisonicotinamide (7zh)**

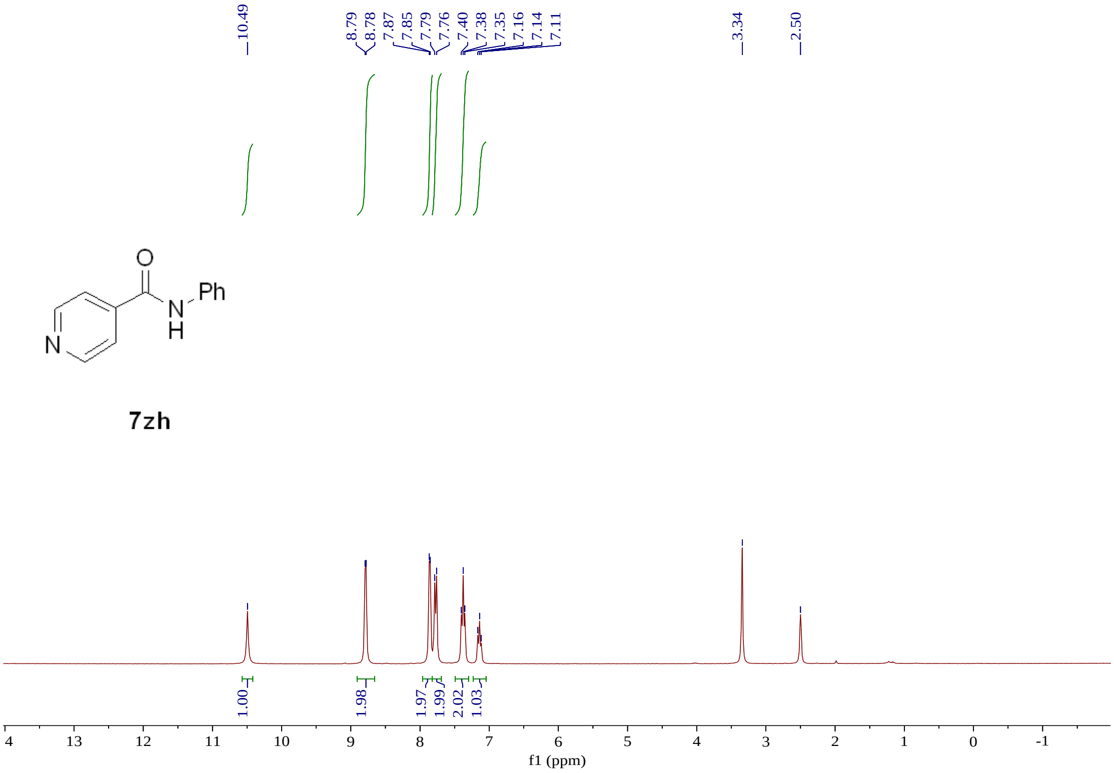


**Supplementary Figure 120.** $^{1}$H NMR Spectra of **7zh.**

**(1*R*)-((2*S*,4*S*,5*R*)-5-Ethylquinuclidin-2-yl)(6-methoxyquinolin-4-yl)methyl 4-(phenylcarbamoyl)benzoate (7zi)**

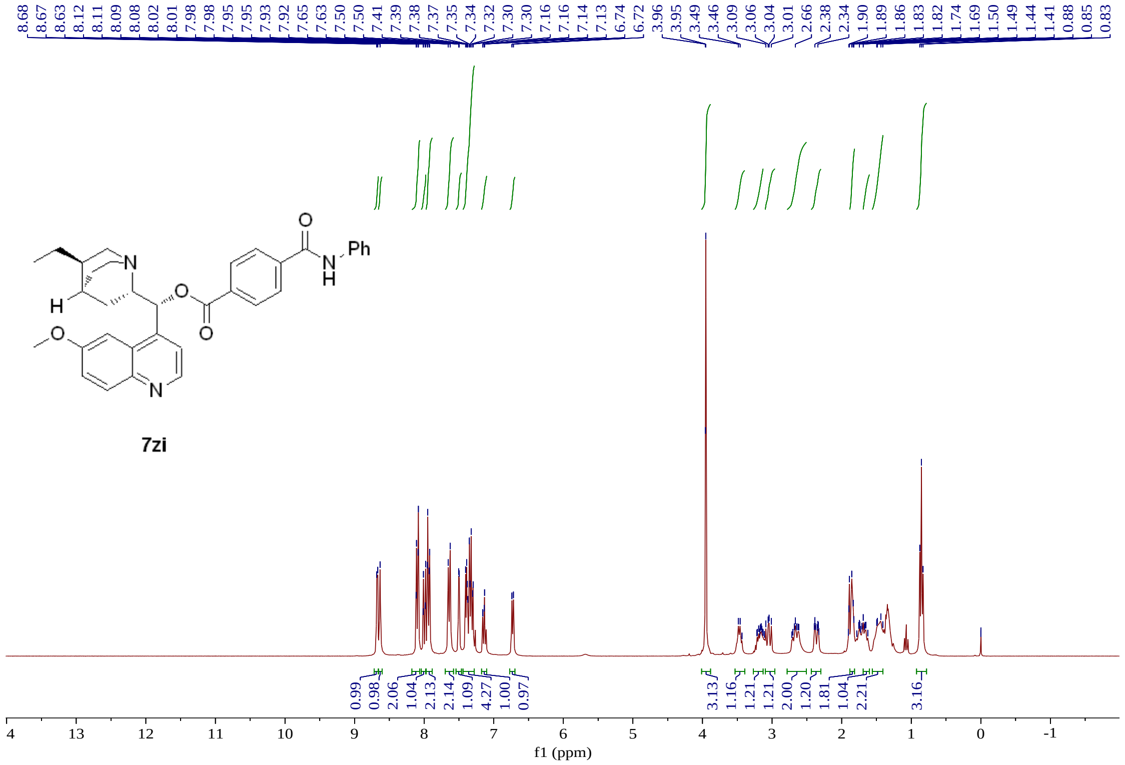

**Supplementary Figure 121.** $^{1}$H NMR Spectra of **7zi.**

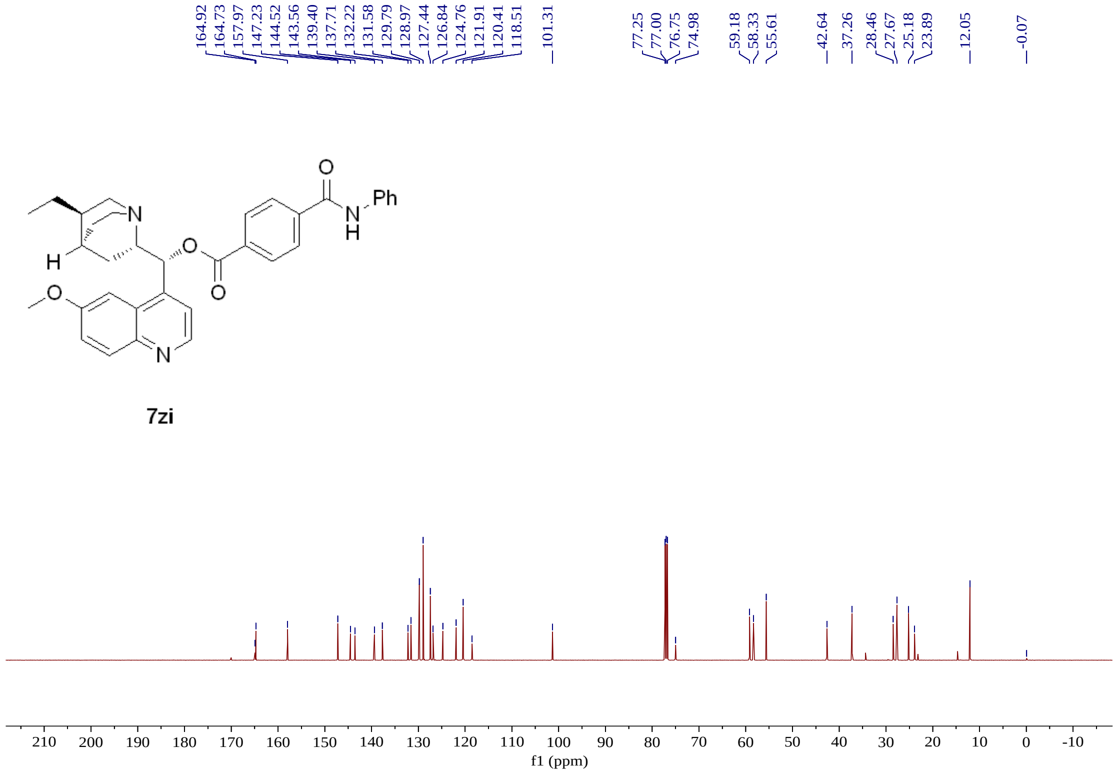

**Supplementary Figure 122.** $^{13}$C NMR Spectra of **7zi.**

**2-(Diethylamino)ethyl 4-(phenylcarbamoyl)benzoate (7zj)**

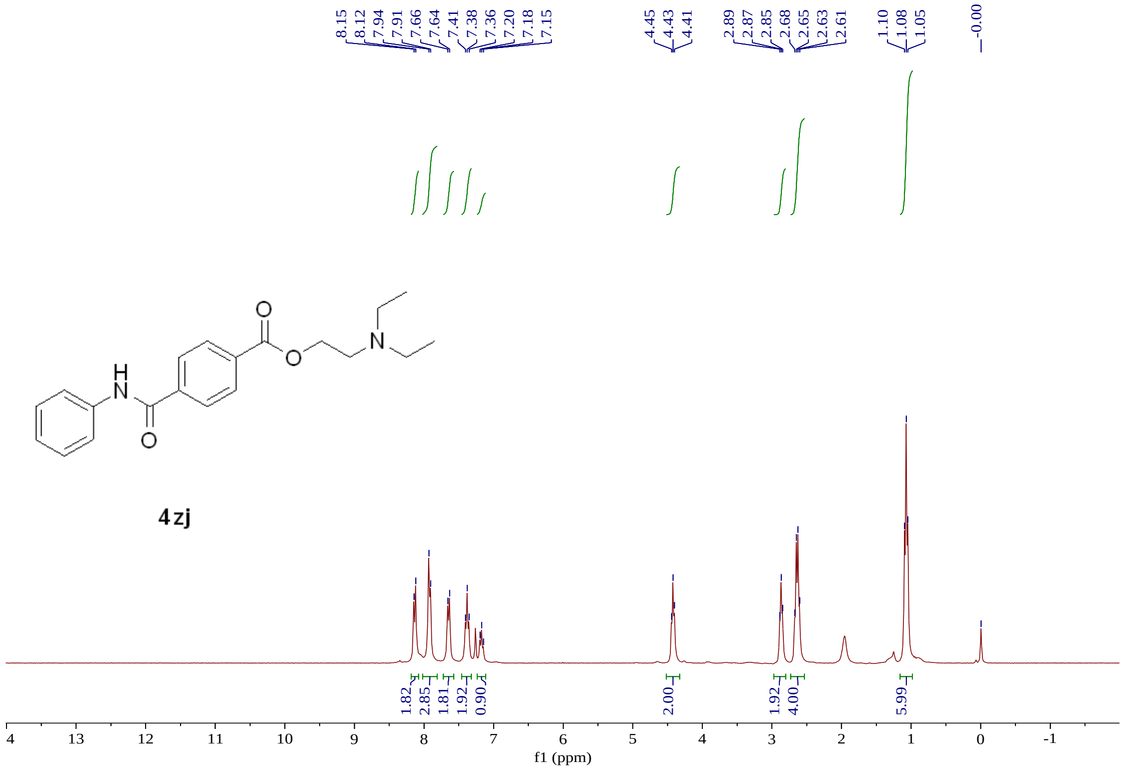

**Supplementary Figure 123.** $^{1}H$ NMR Spectra of **7zj.**

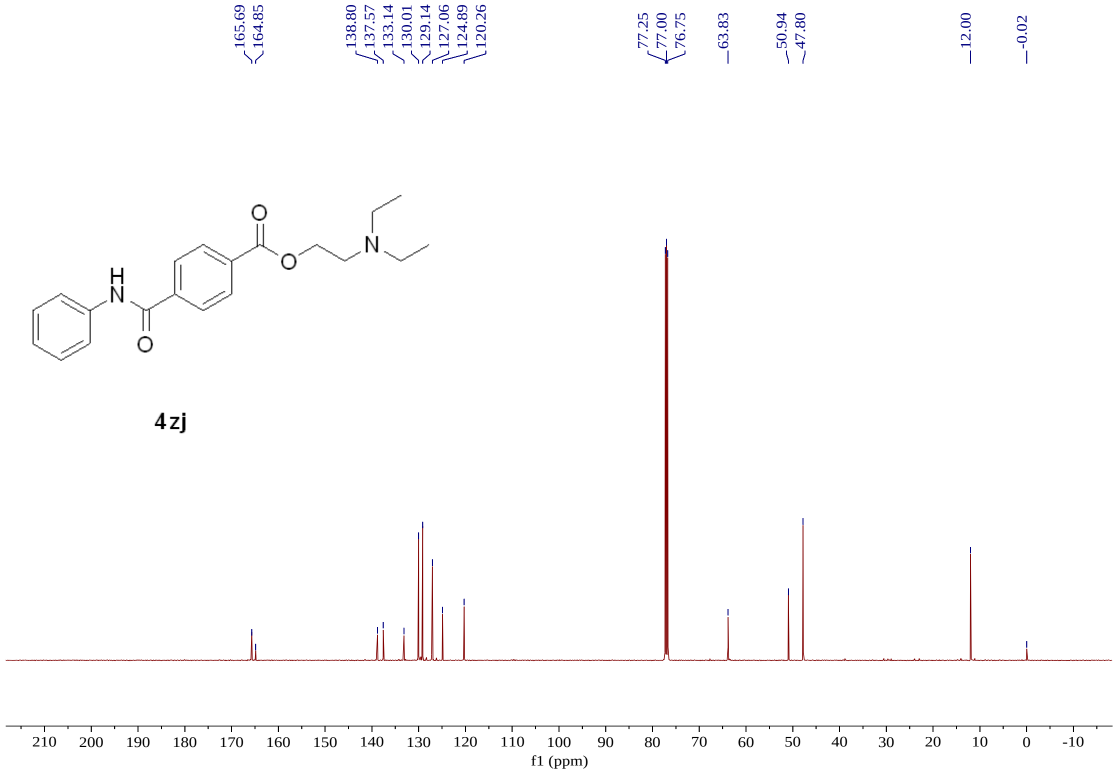

**Supplementary Figure 124.** $^{13}C$ NMR Spectra of **7zj.**

**Phenyl [1,1'-biphenyl]-4-carboxylate (8a)**

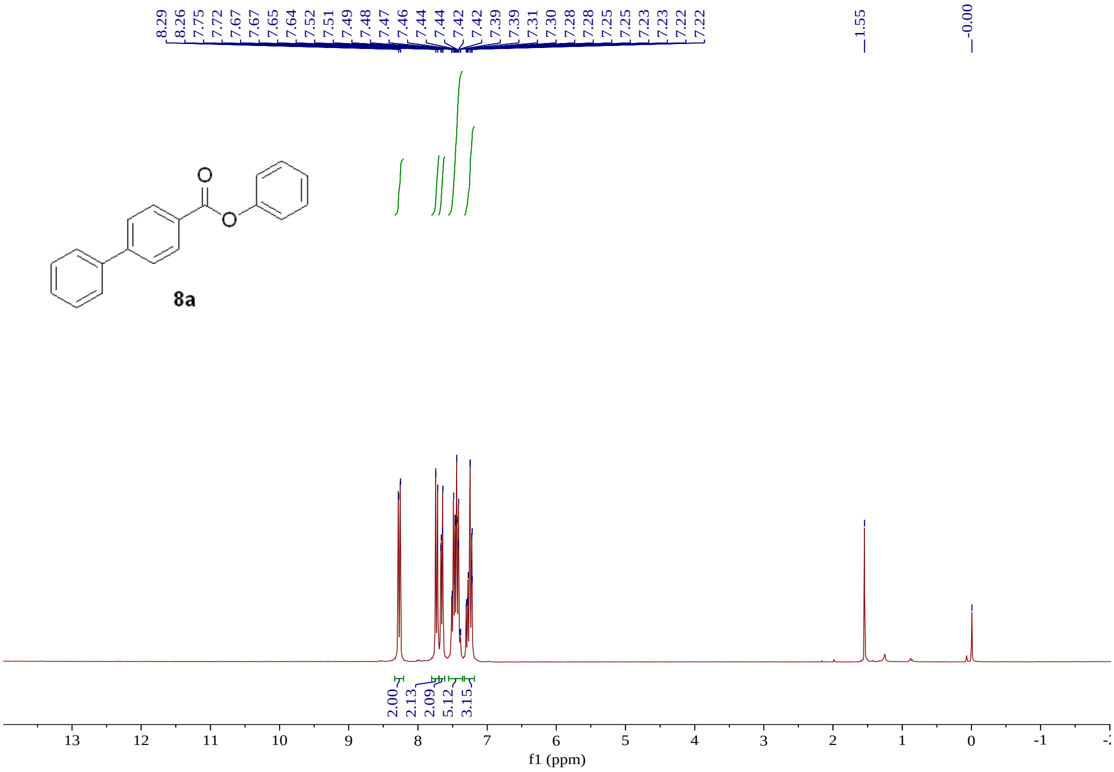


**Supplementary Figure 125.** $^{1}$H NMR Spectra of **8a.**

***S*-*p*-Tolyl [1,1'-biphenyl]-4-carbothioate (9a)**

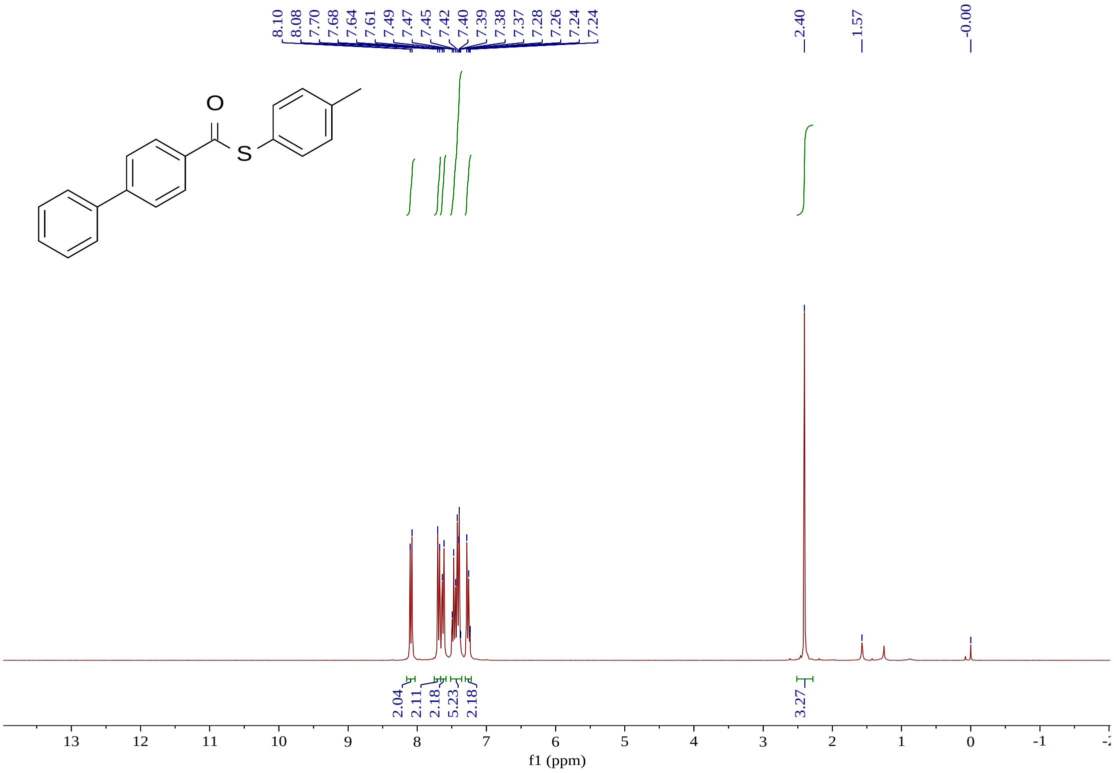


**Supplementary Figure 126.** $^{1}$H NMR Spectra of **9a.**

**[1,1'-Biphenyl]-4-yl(phenyl)methanone (10a)**

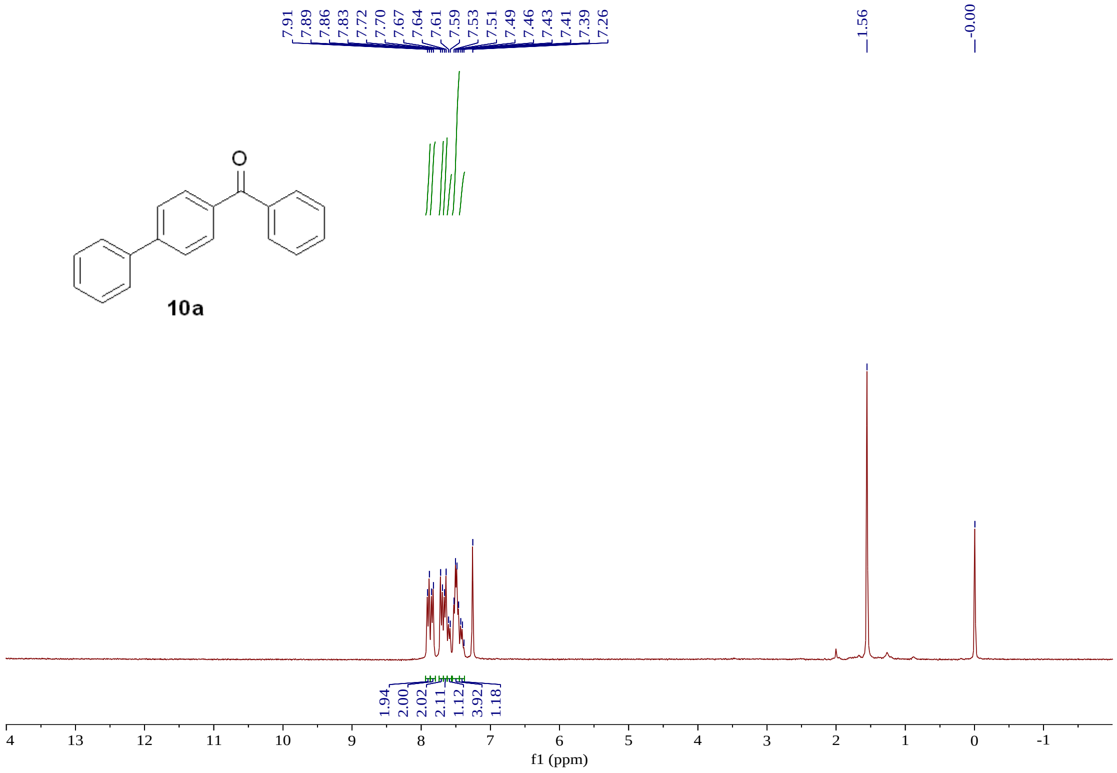


**Supplementary Figure 127.** ¹H NMR Spectra of **10a.**

**[1,1'-Biphenyl]-4-ylmethanol (11a)**

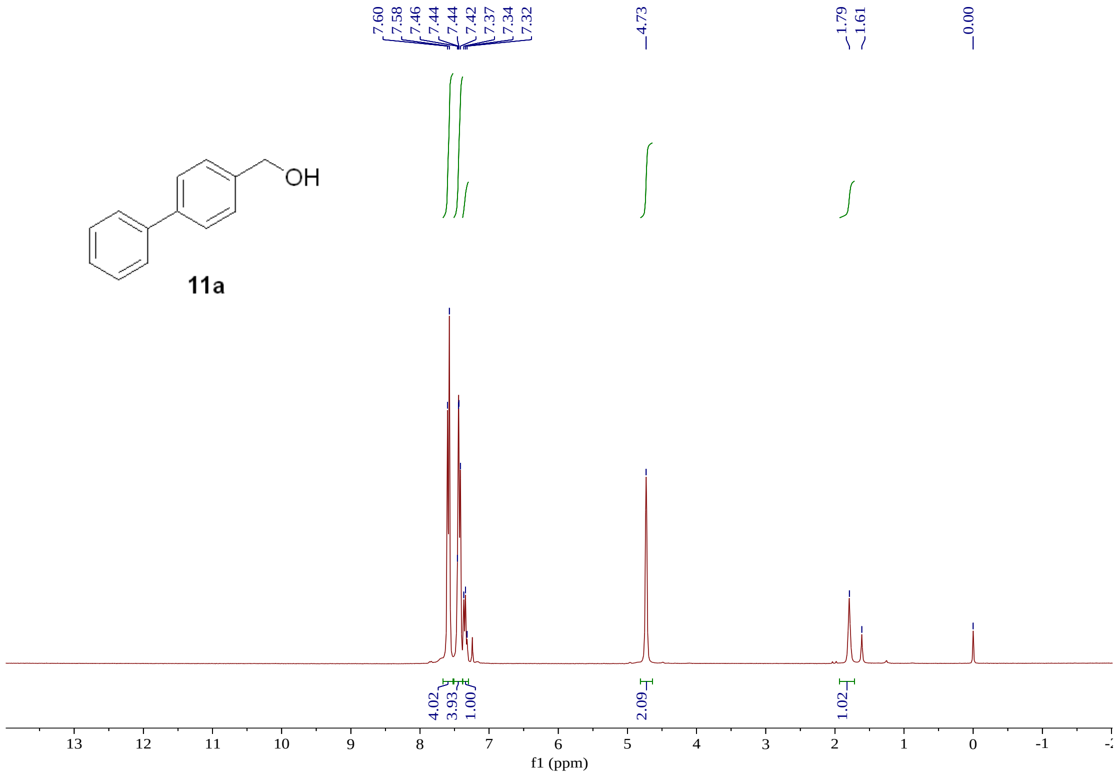


**Supplementary Figure 128.** ¹H NMR Spectra of **11a.**

**[1,1'-Biphenyl]-4-carboxylic acid (12a)**

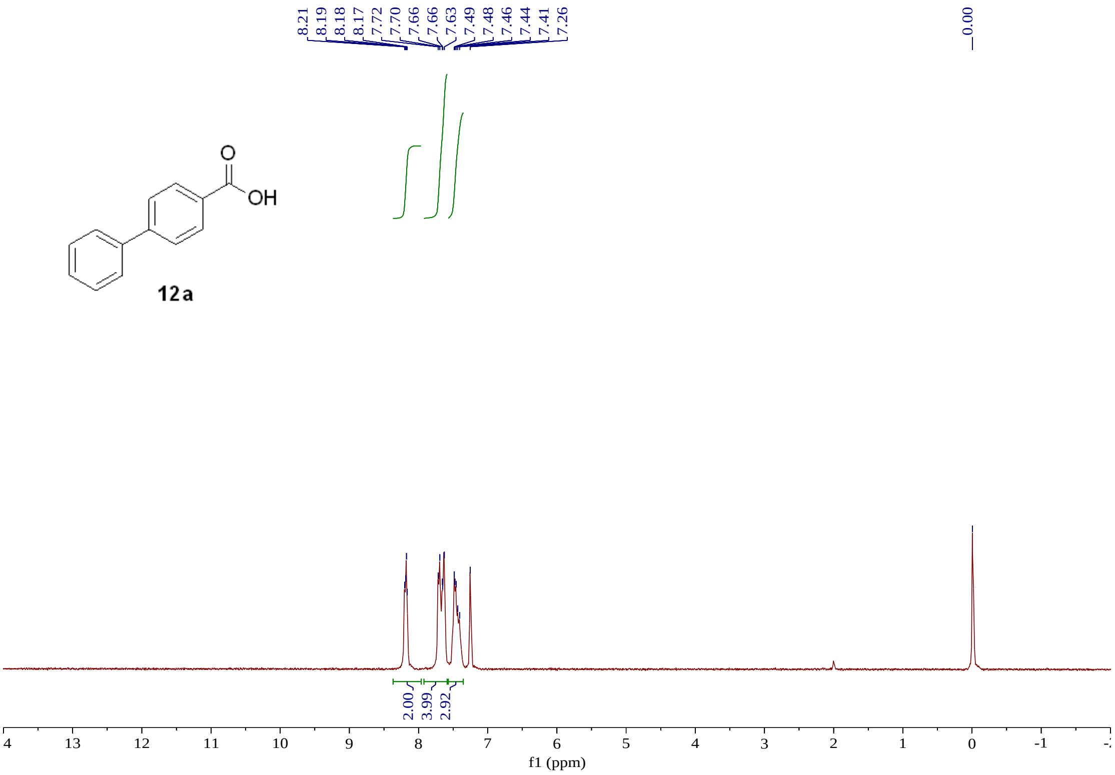


**Supplementary Figure 129.** $^{1}$H NMR Spectra of **12a.**

**[1,1'-Biphenyl]-4-carbaldehyde (13a)**

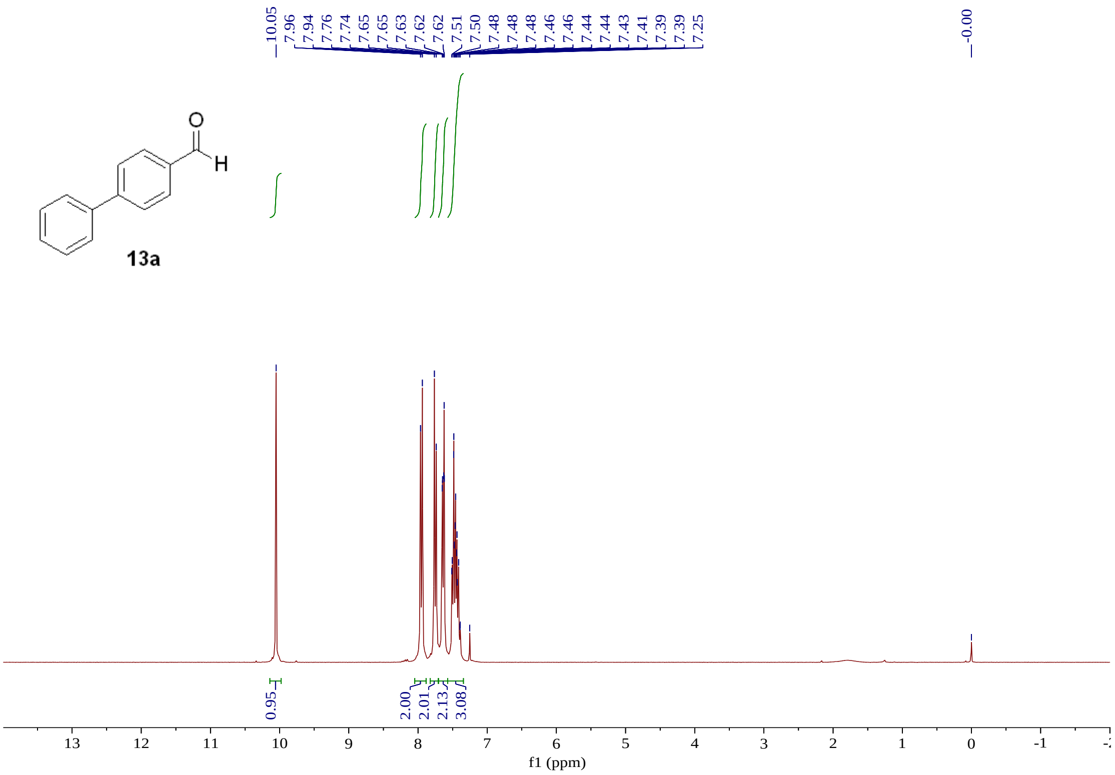


**Supplementary Figure 130.** $^{1}$H NMR Spectra of **13a.**

**1,1'-Biphenyl (14a)**

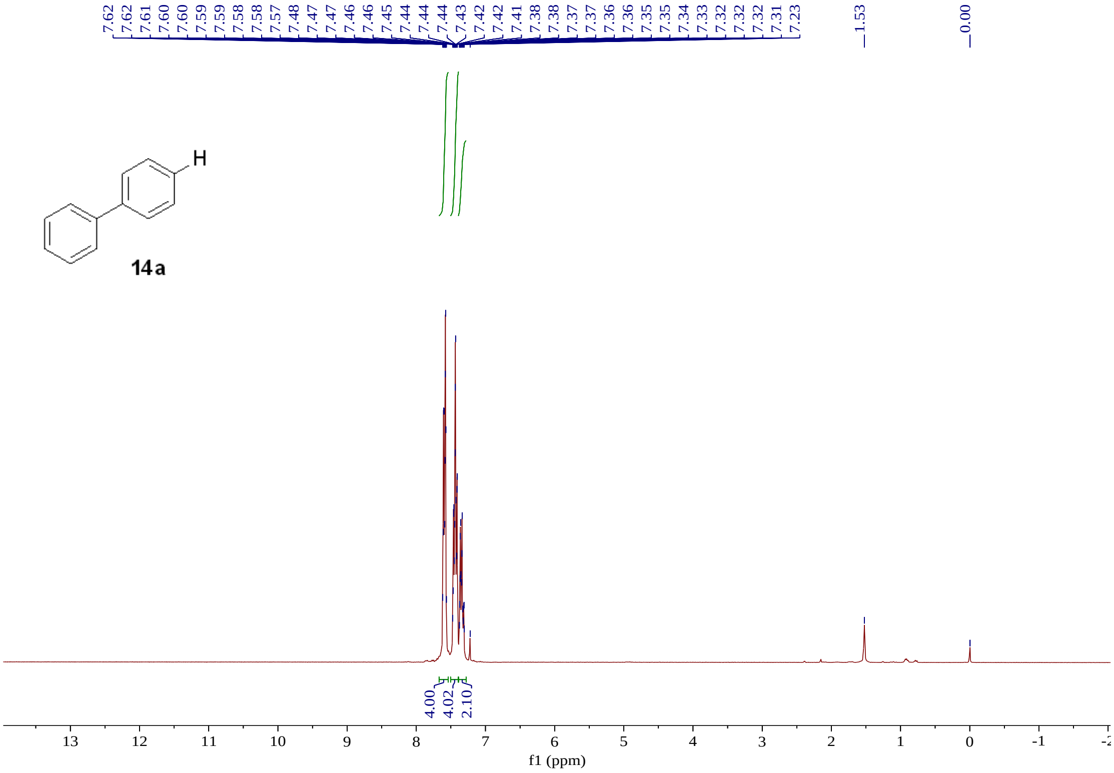


**Supplementary Figure 131.** $^{1}$H NMR Spectra of **14a.**
